# Fox Hunting in Wild Apples: Searching for Novel Genes in *Malus Sieversii*

**DOI:** 10.3390/ijms21249516

**Published:** 2020-12-14

**Authors:** Michael Wisniewski, Timothy Artlip, Jia Liu, Jing Ma, Erik Burchard, John Norelli, Christopher Dardick

**Affiliations:** 1 USDA-ARS-Appalachian Fruit Research Station, Kearneysville, WV 25430, USA; jing.ma@usda.gov (J.M.); erik.burchard@usda.gov (E.B.); Jnorelli60@gmail.com (J.N.); chris.dardick@usda.gov (C.D.); 2 Chongqing Key Laboratory of Economic Plant Biotechnology, Institute of Special Plants, College of Landscape Architecture and Life Sciences, Chongqing University of Arts and Sciences, Yongchuan, Chongqing 402160, China

**Keywords:** apple, forward over expression, fox, *malus pumila*, *malus sieversii*, *malus* × *domestica*, freezing

## Abstract

*Malus sieversii* is considered the progenitor of modern apple (*Malus pumila*) cultivars and to represent a valuable source of genetic diversity. Despite the importance of *M. sieversii* as a source of disease resistance, stress tolerance, and novel fruit traits, little is known about gene function and diversity in *M. sieversii*. Notably, a publicly annotated genome sequence for this species is not available. In the current study, the FOX (Full-length cDNA OvereXpressing) gene hunting system was used to construct a library of transgenic lines of *Arabidopsis* in which each transgenic line overexpresses a full-length gene obtained from a cDNA library of the PI619283 accession of *M. sieversii*. The cDNA library was constructed from mRNA obtained from bark tissues collected in late fall–early winter, a time at which many abiotic stress-adaptative genes are expressed. Over 4000 apple FOX *Arabidopsis* lines have been established from the pool of transgenic seeds and cDNA inserts corresponding to various Gene Ontology (GO) categories have been identified. A total of 160 inserts appear to be novel, with no or limited homology to *M. pumila*, Arabidopsis, or poplar. Over 1300 lines have also been screened for freezing resistance. The constructed library of transgenic lines provides a valuable genetic resource for exploring gene function and diversity in *Malus sieversii.* Notably, no such library of t-DNA lines currently exists for any *Malus* species.

## 1. Introduction

The domesticated apple (*Malus pumila*) is an important agricultural commodity with an average annual production of 4.57 million metric tons in the United States, with a value approaching four billion US dollars [1]. Globally, over 75 million metric tons were produced in 2018/2019 [2]. Despite the abundance of different apple cultivars, domesticated apples have a rather narrow genetic base, with most cultivars deriving from a relatively small number of founders [3,4]. Fire blight (causative agent: *Erwinia amylovora*) results in over $100 million per year in direct losses and control costs [5], while the postharvest disease blue mold (causative agent: *Penicillium expansum*) causes over $4 million in losses per year in the United States alone [6]. Data from the USDA Risk Management Agency for the years 2007–2017 indicates that apple growers claimed $157,177,390 in insured losses from freeze damage [7]. These impacts will continue and may increase due the global transport of new diseases or pathogenic strains, invasive pests and increased levels of environmental stress (temperature, water) brought on by climate change.

**Figure 1:**
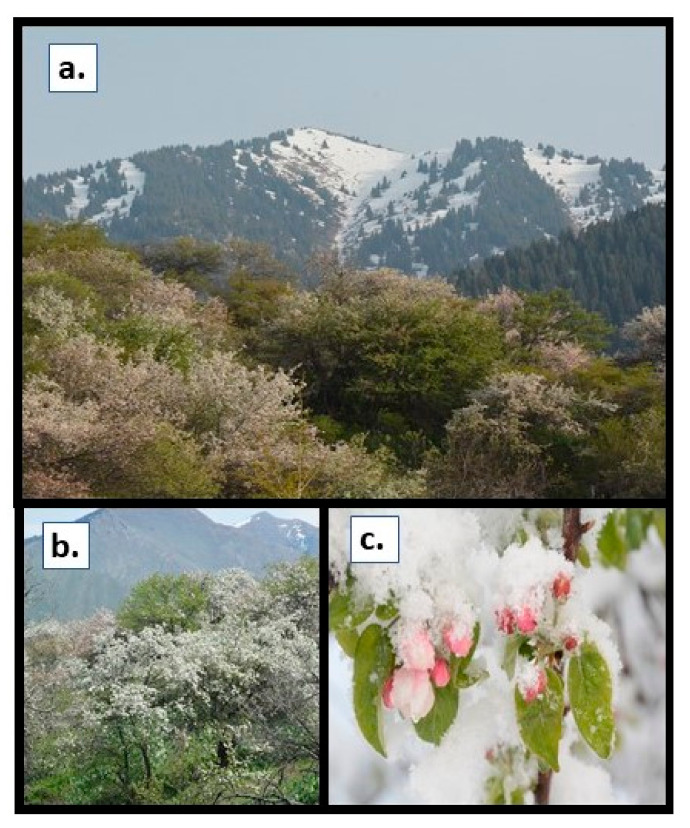
*M. sieversii* during spring, native range. (**a**) Montane habitat of *Malus sieversii* in Kazakhstan. (**b**) Natural occurrence of *Malus sieversii* on a hillside in Kazakhstan. (**c**) Bloom covered by sudden snow. Photos courtesy of Dr. Gayle Volk, USDA-ARS.

*Malus sieversii*, a progenitor of the domesticated apple, represents a reservoir of genetic diversity [8,9]. Native to Central Asian montane forests, *M. sieversii* trees exhibit considerable phenotypic variability [10]. In order to capture and preserve this diversity, budwood and seeds were collected from wild trees growing in Kazakhstan (Figure 1) and planted at the USDA-ARS Apple Germplasm Repository in Geneva, NY, with special emphasis on trees that appeared to have disease and/or drought tolerance [6,10,11]. For example, budwood and seeds of PI613981, collected from a tree growing at a xeric site, were propagated in the USDA-ARS repository and used to establish a mapping population (GMAL 4593) for identifying genetic markers for a variety of segregating traits (scab resistance, bloom time, fire blight resistance, etc.).

Introgression of desirable traits in domesticated crops has been aided by greater availability of genome sequences and Marker Assisted Selection (MAS). The absence of a readily available genome sequence for *M. sieversii,* however, has made trait introgression using MAS difficult. The two *M.* × *domestica* (formally recognized as *M. pumila*) genomes available at the Genome Database for Rosaceae (GDR, [12,13,14,15]) are replete with gene models that have no known function or no known homology to model species such as *Arabidopsis*. Thus, only a few avenues exist for exploring genetic diversity in *M. sieversii*. One possible approach relies on the utilization of insertional events into model plant species, such as poplar, as a means of identifying functional genes. T-DNA and transposon tagging has been described by Fladung et al. [16], while Groover et al. [17] employed a similar tactic using gene and enhancer-trap tagging. The application of these approaches is problematic for *M. sieversii* as they rely on creating transgenic plants in the same parent species. Transformation systems for *M. pumila* rootstocks and cultivars do exist; however, rates of transformation and regeneration are so low that they preclude apple as a candidate for high-throughput gene discovery systems [18,19,20,21,22,23]. Furthermore, tissue culture conditions for the propagation and genetic transformation of *M. sieversii* have not been established. The ability to generate a sufficient number of transformants to capture genetic diversity in *M. pumila* or *M. sieversii* would also require considerable space, effort, and time [24]. Lastly, it is unknown how susceptible *M. sieversii* is to vitrification (hyperhydricity) in culture, which can greatly reduce the number of viable regenerants or grossly distort regenerant morphology.

The Full-length cDNA OvereXpressing (FOX) gene hunting system [25,26,27] is an efficient system for studying gene function and diversity in both genetically characterized and uncharacterized plant species. This gain-of-function approach requires the creation of a normalized, full-length cDNA library from tissue(s) and/or stage of development of interest, followed by mass transformation of the obtained cDNAs into a model system plant, such as *Arabidopsis thaliana* or rice (*Oryza sativa*). Progeny can then be screened for abnormal phenotypes or specific traits such as abiotic stress tolerance (low/high temperature, salinity, hyperosmotic conditions), architecture, disease resistance, etc. The FOX hunting system has been used to identify and study functional genes from rice [28,29,30,31], lotus (*Lotus corniculatus*) root cultures [32], wheat transcription factors [33], *Brassica napus* [34], and the halophyte *Ipomoea pes-caprae* [35].

Genes identified using the FOX hunting system have potentially important functions in agricultural crops. Nakamura et al. [29] reported that approximately 16.6% of the 5462 independent transformed *Arabidopsis* lines with rice genes had dominant altered phenotypes. Of particular interest were three super-dwarf lines harboring a novel *gibberellin 2-oxidase* gene. Kondou et al. [30] screened a similar number of *Arabidopsis* lines containing individual rice genes and reported a wide variety of aboveground morphological phenotypes (leaves, height, growth habit, and pigment) along with various root phenotypes. Himuro et al. [32] screened a lotus root FOX-hunting *Arabidopsis* library for what they termed “superroots” for potential use in a breeding program in lotus. High throughput screening for abiotic stress resistance, particularly for high salinity soil conditions, allowed Zhang et al. [35] to identify and evaluate 38 genes from *Ipomoea pes-caprae.* While the FOX hunting system relies on heterologous expression which may not allow the identification of crop-specific phenotypes, it provides the opportunity to explore gene function and diversity in recalcitrant systems like apples [25].

In the current study, cDNA from an accession of *M. sieversii* (PI613981) was used to create a library of apple FOX Arabidopsis overexpression lines. PI613981 was initially obtained from a xeric site and collected as a potential source for drought resistance or tolerance. A GMAL4593 mapping population (*M. sieversii* “PI613981” × “Royal Gala”) was previously used to identify quality trait loci (QTLs) for resistance to the postharvest pathogen, *Penicillium expansum*, the causal agent of blue mold [6]. In that study, *M. sieversii* was found to be the main contributor to blue mold resistance and the mapping population was used to identify a genetic marker for blue mold resistance in apples. Currently, over 4000 *M. sieversii* FOX lines have been established, with nearly 2900 unique inserts. Among the genes present in the library of transformed plants, numerous genes known to be associated with abiotic stress signaling, dormancy, and growth and development were identified.

## 2. Results

To date, over 4000 independent T2 lines have been entered into a local database, with 2888 inserts annotated by BLASTN comparison to the “Golden Delicious” Doubled Haploid (GDDH) genome (GDR) and the Non-Redundant National Center for Biotechnology Information (NCBI) mRNA database (Supplementary Materials Tables S1 and S2). One hundred eighty-three lines were found to have inserts from vectors, microbial sources, or human genes. Eight hundred thirty-four lines had low-quality reads (i.e., E-values ≥ 1 × 10^−50^), with matches frequently listed as “protein of unknown function”. A further 75 lines were deemed questionable, with weak E-values (0.0 ≥ × ≥ 1 × 10^−50^) with matches to *Malus/Pyrus* (pear) gene models or GDDH matches coupled with NCBI matches to vector sequences. At the time of this manuscript’s preparation, 35 lines with weak E-values matching *Malus* in both the GDDH and NCBI databases were noted but deemed inconclusive in terms of identity. Of these lines, eight were annotated as unknown, domain of unknown function or uncharacterized.

**Figure 2:**
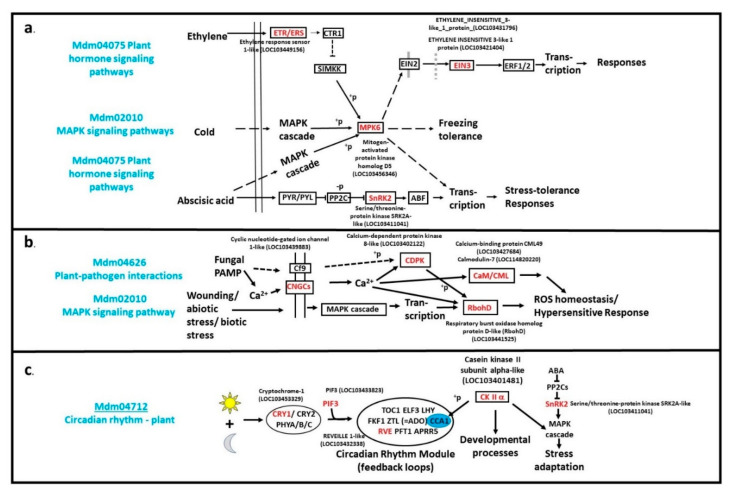
KEGG GO pathways of selected apple Full-length cDNA OvereXpressing (FOX) Arabidopsis mutant lines. Pathways reflect known genes from *Malus* × *domestica* but are not necessarily specific to *M*. × *domestica*. Gene names in red indicate apple FOX inserts. (**a**) Plant hormone signaling pathways and MAPK/cold signaling pathway. (**b**) Plant pathogen and MAPK signaling pathways. (**c**) Plant circadian rhythm pathways. Adapted from KEGG [36,37].

Nonduplicated transcripts were present in the majority of the lines, with 1500 of the 1800 high-quality inserts being unique, while 300 were redundant. An 83% level of nonduplication can be attributed to the fact that the cDNA library was normalized to minimize overrepresentation of abundant transcripts. A BLAST analysis of the unique inserts was conducted using the NCBI database, and the resulting LOC/GeneID numbers were used to query the KEGG database [36,37]. Currently, 115 GO categories are represented in the obtained lines (Supplementary Materials Table S3). The metabolic pathways category alone contained nearly 180 different cDNA inserts, with numerous representatives in biosynthesis of secondary metabolites (85 inserts), carbon metabolism (33 inserts), and biosynthesis of amino acids (28 inserts), suggesting that apple bark cells remain metabolically active at this winter time-point. It is possible that *M. sieversii* bark was responding to various environmental cues at the time of sampling (Figure 2). Inserts for various plant growth regulator signal transduction pathways were present in the library, including those associated with ethylene and abscisic acid (ABA) signaling pathways (Figure 2a). Some of the cDNA inserts could be classified in more than one pathway. For example, a Mitogen Activated Protein Kinase (MAPK) was categorized as a component of multiple signal transduction pathways for ethylene, abiotic stress responses (drought, cold), and biotic stress responses, as well as in the KEGG MAPK GO category (Figure 2a). Abiotic stresses generally induce Reactive Oxygen Species (ROS) accumulation and the need to mitigate potential oxidative stress. ROS is also a component of biotic stress responses and components of both of these ROS responses were also identified (Figure 2b). Casein Kinase II α, identified as an insert in one of the lines, is a component of the circadian rhythm KEGG GO category, but also participates in other pathways not recognized by KEGG, such as regulation of the ABA signal transduction pathway (Figure 2c).

A more in-depth analysis was subsequently conducted of abiotic stress-related inserts and those that could potentially be involved in dormancy, since cold acclimation and dormancy feature overlapping regulons in temperate woody perennials [38]. Results indicated that functional abiotic stress genes, such as heat shock proteins, members of the glutathione pathway/ROS detoxification pathways, and the Late Embryogenesis Abundant (LEA) family (including dehydrins), were among the cDNA inserts present in the FOX library (Supplementary Materials Table S4). Inserts encoding biotic stress-related genes were also found, including pathogenesis-related (PR) proteins, numerous disease resistance proteins (TIR-NBS-LRR class), leucine-rich receptor-like kinases, and MAPK signaling components (Supplementary Materials Table S5). A few genes associated with dormancy were identified (Supplementary Materials Table S6), including a Dormancy Associated MADS-box (DAM) gene, MdDAM1/MdDAMa, along with a vernalization 5/VIN3-like gene. Many of the inserts broadly associated with dormancy processes are associated with epigenetic mechanisms, such as methyltransferases, chromatin remodeling factor CHD3, a histone acetylase, and two additional histone deacetylases.

**Figure 3:**
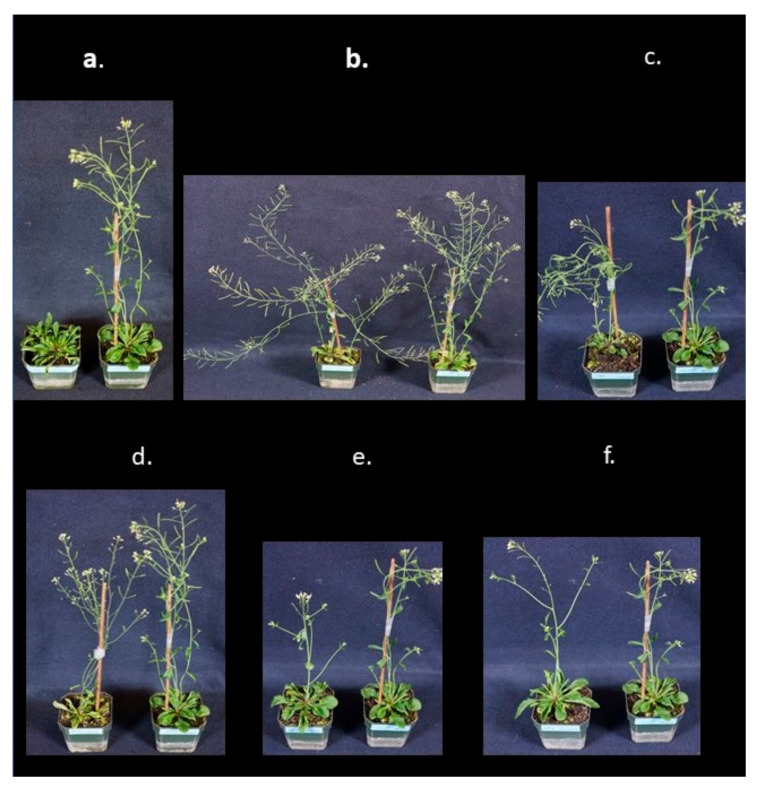
Morphological phenotypes of apple FOX *Arabidopsis* transgenic lines. C0 is nontransformed Arabidopsis ecotype Columbia 0 (C0). (**a**) Line 145, left, with delayed growth and flowering; C0, right. (**b**) Line 167, left, with thick stem, wide branch angles and long branches; C0, right. (**c**) Line 186, left, with spindly stem and small leaves; C0, right. (**d**) Line 216, left, with spindly stem and tiny leaves; C0, right. (**e**) Line 236, left, with short, thick stem; C0, right. (**f**) Line 263, left, with thick, tall stem and few flowers; C0, right.

A number of altered morphological phenotypes, relative to wild-type plants, were observed among the FOX hunting lines. These included alterations in stems, leaves, flowering habits, and seeds. The types of stem phenotypes included variations in thickness (normal, thick, thin, spindly), height (normal, short, tall), stem architecture (number of primary bolts and lateral branches), and branching (normal, few, many). Assessment of leaf types was limited to size (normal, small, large) and number (normal, few, many). Recording of flowering phenotypes was also restricted to time of flowering (normal, early, delayed) and number of flowers (normal, few, many). The number of seeds produced was typically proportional to the number of flowers produced; however, in some cases no seeds were produced, suggesting either a dominant lethal or a lethal insertion event. Lastly, variations in growth habit phenotypes (normal, upright, prostrate, compact, weepy) were also observed. Examples of various phenotypes are shown in Figure 3. The cDNA inserts associated with these lines are summarized in Table 1. These inserts were unique within the database at the time this manuscript was written and additional examples are being sought to confirm whether the morphologies are due to the inserted cDNAs or the insertion position of the cDNAs.

**Figure 4:**
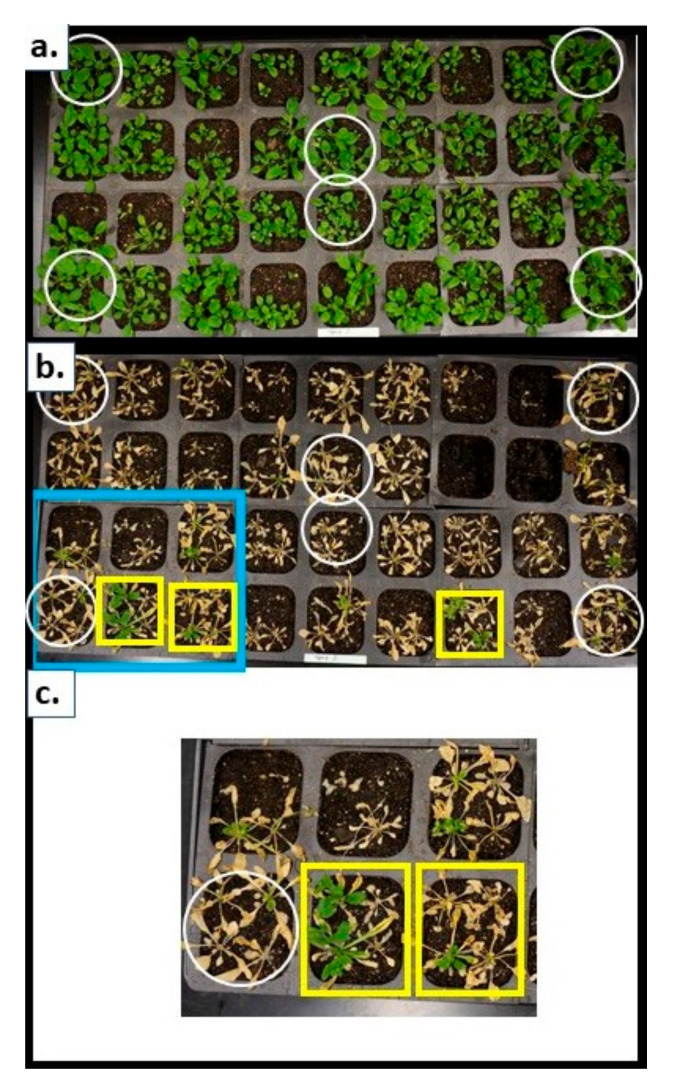
Screening apple FOX Arabidopsis lines for freezing tolerance. Three-week old seedlings were subjected to the following freezing program: −1 °C/h to −6 °C, 2 h soak at −6 °C, +1 C/h to 0 °C, soak for 4 h, +2 °C/h to 5 °C, hold. The cell packs were then removed and returned to standard growth conditions. The plants were then scored as living/dead one week later. (**a**) Prior to freezing. (**b**) One-week after freezing. (**c**) Close up of cell pack outlined in blue in panel (**b**) with surviving apple FOX Arabidopsis seedlings. White circles: untransformed C0 controls. Yellow squares: individual cells/ transgenic lines with potentially surviving apple FOX Arabidopsis seedlings.

There were two main objectives in constructing the *M. sieversii* FOX Arabidopsis library. One was to provide a comprehensive library of transgenic lines carrying individual cDNA clones of *M. sieversii* for the purpose of studying gene function and diversity in *M. sieversii* during cold acclimation and dormancy. The second was to use the library to empirically screen for gene inserts that have potential economic importance in commercial apple cultivars. In regard to the latter, over 1300 lines were subjected to a stringent freezing tolerance screen consisting of a gradual temperature decrease (−2 °C/h) from 0 °C, followed by a one-h exposure to −6 °C, and a gradual increase (+2 °C/h) to +10 °C; plants were sprayed with Ice Nucleation Active (INA) bacteria at 0 °C to ensure freezing initiation at warm sub-zero temperatures [39]. Plants were scored as living or dead one week after their return to normal growth conditions (22 °C, 16-h light period, 50–100 µmole photons m^−2^ s^−1^ PAR; Figure 4). Preliminary tests indicated that −6 °C was required to kill untransformed C0 plants using the designed freezing tolerance assay. The lowest temperature (−6 °C) was established in a series of steps so that the freezing protocol would be physiologically relevant. Flats containing the plants were first allowed to equilibrate to 0 °C so that latent heat from the potting soil would not unduly influence the temperature of the plants. The cooling rate of −2 °C/h was a rate that could be expected in a natural environment [40]. While exposure to −6 °C may seem severe, it is a temperature used in other studies (e.g., [41]).

The assay used in the current report allowed up to 60 FOX lines and 12 untransformed C0 plants to be screened per assay. Two to three assays were conducted each week and over 1300 lines were assessed. Four plants of each line (thirty lines total) and twenty-four untransformed control plants were typically used in each individual screen, thus allowing greater confidence in positive or negative results. A 50% survival rate was used as the threshold for further evaluation, with some lines exhibiting 75% survival; however, no lines exhibited 100% survival. A total of 8 out of 1300 tested lines met the 50% survival threshold and underwent expanded testing (Table 1). The FL-cDNAs in these lines included a dehydrin, a β-amylase7, a putative thiol-disulphide oxidoreductase/uncharacterized YuxK-like protein, a chloroplastic DegP protease, an insert with no known homology, a member of the protein kinase superfamily that matched a NTF6-like mitogen-activated protein kinase (MAPK) homolog, and a gene for casein kinase II α1 (Table 2). These lines were subjected to expanded testing, with 150 FOX plants and 30 C0 plants per test. The results were equivocal at best, with the percent survivability of the FOX lines comparable to that of the C0 plants (Table 2). At worst, all the FOX plants were killed along with the C0 plants. These data suggest that the acceptance criteria used in the high-throughput tests should be a minimum of 75% survivability and that the number of plants should be increased to five per cell.

## 3. Discussion

The primary objective of this study was to construct a FOX hunting library of transformed lines of *Arabidopis* plants, each line containing a full-length sequence of a cDNA obtained from winter-acclimated bark tissues of *M. sieversii* (PI 613981). This library could then be used to study gene function and diversity in this apple progenitor species. *M. sieversii*, is reported to be the main progenitor of the domesticated apple (*M. pumila*) along with *M. sylvestris* (L.) Mill., *M. orientalis* Ulglitzk., and *M. prunifolia* (Wild.), the latter three representing minor contributors [14,15,42,43].

*M. sieversii* naturally occurs in environments that vary in temperature, water availability, and elevation. Therefore, it is considered to be an important reservoir of genetic diversity for many biotic and abiotic stress tolerance traits [10,44,45]. Some *M. sieversiii* genotypes have also been recognized for their fruit-quality traits [9,10]. Jurick et al. [46] identified postharvest disease resistance to both blue mold (*Penicillium expansum*) and *Colletotrichum acumatum* and Norelli et al. [6] used the GMAL4593 (PI 613981 × “Royal Gala”) mapping population to identify a major QTL (contributed by the *M. sieversii* parent) for blue mold resistance. *M. sieversii* as a rootstock has been reported to maintain the relative growth rate of the scion genotype during an imposed water deficit [47], and Bassett et al. [48] and Bassett [49] suggested that xeric tolerance could be potentially attributed to superior water uptake and/or specific biochemical adaptations. These studies reinforce the premise that *M. sieversii* represents an important source of valuable biotic and abiotic stress-tolerance traits that may be useful in long-range apple breeding programs.

The lack of a publicly available genome database for *M. sieversii* and reliable high-throughput transformation methods represent a hurdle for studying gene function and diversity in apples. Therefore, the construction of a FOX Arabidopsis seed library of full-length *M. sieversii* cDNAs from winter-acclimated bark represents a valuable tool for researchers in apple biology and for the identification of genes underlying economically important traits. Although introgression of valuable traits from *M. sieversii* into *M. pumila* may require an extended period of breeding due to the genetic drag of unwanted genes from *M. sieversii,* the use of improved genetic maps, marker-assisted selection, and newly developed rapid-cycle breeding technologies should greatly assist in overcoming this problem [50].

Approximately 42,000 to 45,000 genes are estimated for the doubled haploid and diploid “Golden Delicious” genomes [15] and the ability to readily create interspecific hybrids between *M. sieversii* and *M.* × *domestica* [43], and their similar genome sizes [51,52], suggest that a similar number of protein coding and noncoding regions exist between them. In the present study, approximately 4300 lines have currently been sequenced. After discounting bad reads, lines with contaminant inserts, and questionable or low-quality reads, 1500 high quality lines containing 1300 different FL-cDNAs have been established in *Arabdiopsis* and seed banks for each of these lines have been created, while further development of several thousand more lines from the uncharacterized seed lot derived from transformed plants is in progress. Notably, the characterized and noncharacterized portions of the library are believed to be significantly enriched in genes related to abiotic and biotic stress tolerance based on cDNA characterization of winter bark tissues [53,54].

A majority of the apple FOX inserts in the present study could be assigned to GO metabolic pathways (Supplementary Materials Table S3). Our analysis suggests that apple bark tissues are metabolically active during endo- and ecodormancy, even with maximum and minimum air temperatures of 0.56 ± 2.4 °C and −6.30 ± 4.70 °C, respectively, recorded for the seven days prior to when tissues were harvested. Notably, air temperatures and bark tissues can be much higher if they are directly exposed to the sun. Low or freezing temperatures expose plants to several overlapping abiotic stresses including ice formation, dehydration, and excess ROS production [55]. This was readily apparent thanks to the number of abiotic-stress-related genes that have been thus far identified in our library. These include inserts encoding proteins associated with ROS detoxification, cold/drought tolerance/resistance, and components of the ABA and ethylene signal transduction pathways (Supplementary Materials Table S4). These inserts are similar to the transcriptome analysis of eco- and endodormant poplar bark reported by Park et al. [56], and also noted by Artlip et al. [57], in endodormant apple bark. The identification of dehydrin, LEA and Heat Shock Protein (HSP)/chaperone genes in our library also reflected an elevated level of stress tolerance at the time of sampling, as these proteins are known to be associated with abiotic stress tolerance [58,59,60]. These results confirm the value of the created library for studying gene function and diversity in *M. sieversii* through screening the entire library or subcloning specific genes that have already been identified and used to generate independent seed lots.

The variety and number of inserts encoding proteins associated with biotic stress defense also suggests that apple bark tissues actively express disease resistance genes even when they are endo- or ecodormant (Supplementary Materials Table S5). Wisniewski et al. [54] also reported the presence of a high number of defense-related Expressed Sequence Tags (ESTs) in cold-acclimated bark tissues of apple, as did Artlip et al. [57]. While some of these encoded proteins may also function as bark storage proteins (BSPs; [61]), a majority of the inserts were identified as biotic stress-related encode receptors for fungal and bacterial pathogens. *M. sieversii* is also noted for its disease resistance in its natural range and this was a key factor in prioritizing its collection [9,10].

*Malus sieversii* was endodormant at the time of collection. This is reflected in the presence of dormancy-related FL-cDNAs in the apple FOX Arabidopsis lines (Supplementary Materials Table S6). This includes *MdDAM1*/*MdDAMa*, which has elevated expression levels in apple bark and buds during late autumn, winter, and early spring [62]. *MdDAM1/MdDAMa* plays a major role in apple bud dormancy [63], and it is likely that reduced temporal expression of of the gene in *M. sieversii* in its natural range could reduce its ability to withstand harsh winters. A comparison of the promoter elements of *MdDAM1/MdDAMa* from *M. sieversii* to low chill-variety apples may allow further understanding of dormancy in apples.

Phenotypic variation in apple FOX Arabidopsis lines was observed, with some of the apple FOX Arabidopsis lines differing noticeably from wild-type (“Columbia” ecotype 0 or C0) controls. Alterations in stem growth (thickness, height), leaves (size, number), flowering (early, late, none, few, many), and growth habit were commonly seen in this study (Figure 4) and were quite similar to those reported by Ichikawa et al. [25] and Kondou et al. [30]. Altered phenotypes will be further explored in T3 and T4 generations of plants and by independent transformation studies in Arabidopsis using the FL-cDNA of interest to determine if the phenotype is stably present. Development of several independent lines overexpressing a gene is essential to rule out the possibility that the resulting phenotypes are the result of an insertional event that disrupts a coding gene or promoter, rather than the result of the overexpression of the transgene. Importantly, such duplicated lines may already exist in the constructed library and become apparent as the library is further characterized.

In the current study, overexpression of a dehydrin, a family of genes associated with dehydrative stress tolerance [58], did not confer enhanced freezing tolerance in subsequent testing. The dehydrin, termed *MdDHN8* in the *Malus* × *domestica* literature, is a homolog to the Arabidopsis *COR47* gene, which is strongly cold inducible (e.g., [62]); however, not all dehydrins are explicitly linked to freezing tolerance [58] or they may require the expression of more than one dehydrin gene to effect freezing tolerance. Puhaikenen et al. [64] reported that overexpressing two native Arabidopsis dehydrin genes in Arabidopsis was required to achieve an approximately 2 °C improvement in freezing tolerance. Therefore, further analysis will be required to determine the function of *MdDHN8* in *Malus sieversii*.

## 4. Materials and Methods

### 4.1. Selection of Plant Material

*Malus sieversii* accession PI613981 was originally chosen due to its potential for abiotic stress resistance. The original collection site was described as follows: elevation 910 m; xerophytic; very stony soil, dry; rainfall less than 300 mm; germplasm sample for drought resistance [65]. Budwood of this accession was established at the USDA-ARS-PGRU, Geneva, NY [11].

### 4.2. Library Construction

**Figure 5:**
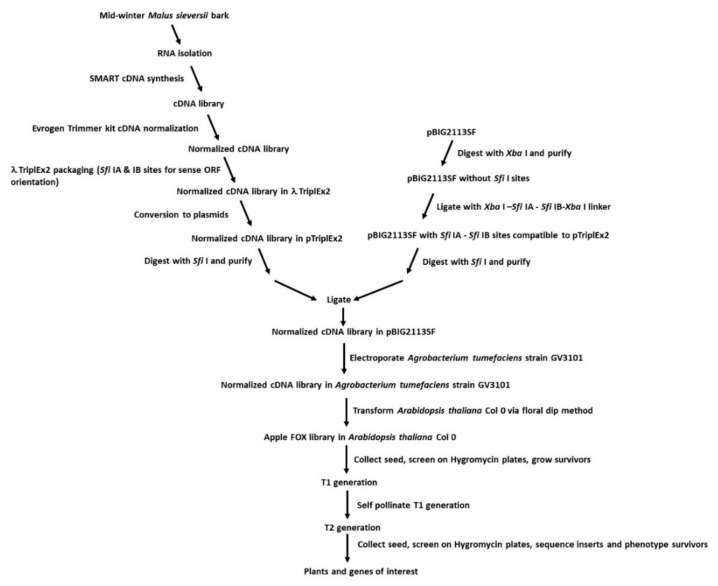
Schematic of *M. sieversii* FL-cDNA, FL-cDNA expression, and FOX Arabidopsis library creation.

Winter-acclimated, current-year bark tissue (phloem, cambium, and epidermis) from one-year-old shoots was destructively sampled (scraped off). Winter-acclimated bark tissue was chosen as possibly unique cold-tolerance or dormancy-related genes should have been abundant. The tissues were flash frozen in liquid N_2_, lyophilized, and stored at −20 °C until use. RNA was isolated using the PureLink™ Plant RNA Reagent (Thermo Fisher Scientific, Waltham, MA, USA). A SMART cDNA library was prepared (Clontech/Takara, Mountain View, CA, USA) and the library normalized per Bogdanova et al. [66], using the Evrogen Trimmer cDNA Normalization kit (Evrogen, Moscow, Russia). The normalized cDNA library was packaged into pTriplEx2 (Clontech/Takara) and converted into pTriplEx2 following the manufacturer’s protocol. pBIG2113SF (RIKEN, Wako, Saitama, Japan) was digested with *Xba* I to remove the portion of the Multiple Cloning Site (MCS) containing the *Sfi* I sites and purified. The vector was then ligated to a linker containing *Xba* I-*Sfi* IA-*Sfi* IB-*Xba* I sites such that the *Sfi* IA and IB sites matched those of the pTriplEx2 plasmid. The pTriplEx2 library and modified pBIG2113SF plasmid were then digested with *Sfi* I, purified as necessary and ligated together, creating a cDNA library in the modified pBIG2113SF, with all Open Reading Frames (ORFs) in the correct orientation for sense-transcription. *Agrobacterium tumefaciens* strain GV3101 was then transformed by electroporation with the pBIG2113SF cDNA library to create an *Agrobacterium tumefaciens* library representative of the original cDNA library (Figure 5).

### 4.3. Arabidopsis Transformation and Screening

504 pots (10.2 cm × 10.2 cm) of *Arabidopsis thaliana* ecotype Columbia 0 (Col 0 or C0) were grown under standard conditions (22 °C (constant), 16 h day/8 h night, with 50–100 µmole photons m^−2^ s^−1^ PAR (F-40 GroLux fluorescent bulbs)) until flower production started [67]. The Arabidopsis plants were then mass transformed with the pBIG2113SF cDNA library, following Clough and Bent [68]. Seed was subsequently collected per Rivero-Lepinka et al. [67] and screened essentially as described by Clough and Bent [68] with Hygromycin (15 mg L^−1^) as the selective agent. Surviving plants were transferred to autoclaved Metromix 360 soil (SunGro). Subsequent rounds of screening (T2 and T3) were performed as described above.

**Figure 6:**
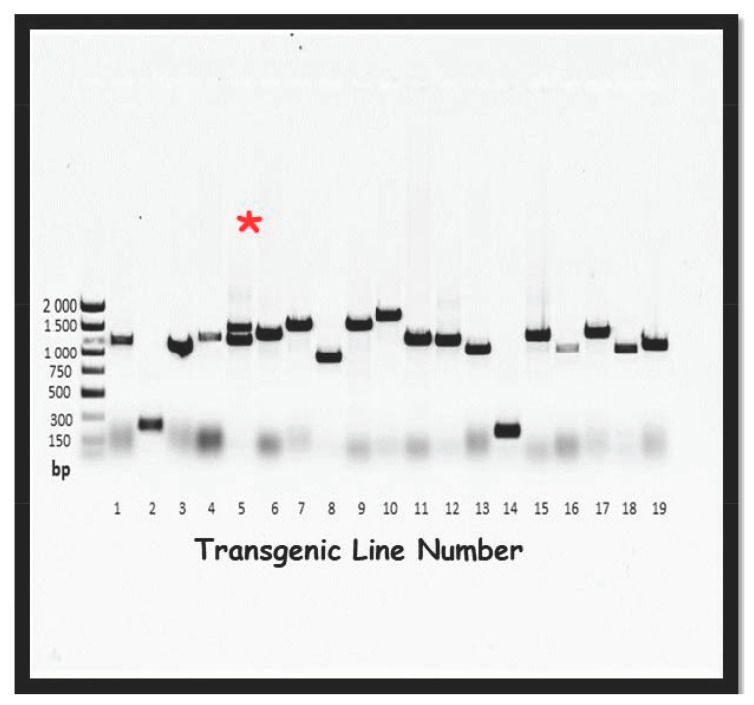
PCR amplification of FOX hunting FL-cDNAs from *Malus sieversii* (PI 613981) inserted into *Arabidopsis*. Note the distribution in the size of the inserts and that some lines have more than one insert. Line 5 is identified as an example of a line with multiple inserts (*).

While each independent line was given a consecutive number in the database, gaps in the available lines exist for several reasons. Some lines were assigned numbers but then subsequently found to have multiple inserts and have since been excluded from further propagation and evaluation (Figure 6). Some gaps were due to lines that had an insert that was fatal in the T2 generation. The majority of gaps, however, were due to sequence data indicating that the lines contained undesirable inserts (vector, microbial, or human), and were subsequently discarded.

### 4.4. Morphological Data

Photographs were taken of T2 plants alongside untransformed C0 plants. Traits including stems (thickness, height, number of stems), leaves (size and number), branching, flowering (timing and number), seed production, habit (upright, compact, leggy, prostrate, weeping) and general comments were recorded.

### 4.5. Insert Sequence Data and Bioinformatics Analyses

Genomic DNA was isolated from approximately 80 mg of leaf tissue using the Quick DNA for Plant/Seed kit (Zymo Research). The DNA was incubated with OneTaq Hot Start (New England Biolabs), in accordance with the manufacturer’s instructions, using the pBIG2113SF R1 primer (5′GGCAACAGGATTCAATCTTAAG 3′), followed by reaction purification using the ZR-96 DNA Clean & Concentrator-5 Deep Well kit (Zymo Research) in a 96-well format. The reactions were submitted to and sequenced by Macrogen (Rockville, MD, USA) in accordance with their specifications. An initial PCR quality check for insert size or multiple insertional events was performed using the genomic DNA samples prior to sequence reactions. The quality control reactions used the same PCR kit and pBIG2113SF R1 primer along with the pBIG2113SF F1 primer (5′GGAAGTTCATTTATTCGGAGAG 3′), per the manufacturer’s protocol.

Sequence data was analyzed by batch BLAST [69] against a local download of the “Golden Delicious” doubled-haploid GDDH13 v1.1 [15], an mRNA database resident and annotation file (GFF3) in the GDR [12,13,15], running the NCBI Blast Command Line Application for Linux [70]. The NCBI database [71] was also queried and the best matches (e value ≤ 1 × 10^−50^) to each database recorded. Additional BLAST analyses were performed for the *Arabidopsis thaliana* genome located in The Arabidopsis Information Resource (TAIR, [72]) on a case-by-case basis.

### 4.6. Freezing Tests

T2 lines were screened on selective media as above, and survivors transferred to autoclaved Metromix 360 soil (SunGro) in 6 cm × 5.4 cm × 5.9 cm “Cell Packs” (insert style 606, Greenhouse MegaStore) and grown at 22 °C (constant), 16 h day/8 h night, with 50–100 µmole photons m^−2^ s^−1^ PAR (F-40 GroLux fluorescent bulbs). Nontransformed C0 plants were simultaneously grown in the same manner and occupied 6 of the 36 cells. The plants were thinned to four or five plants per cell and grown for approximately three weeks or until just prior to bolting. The cell packs were placed in an environmental chamber (Tenney Environmental Model T20S-1.5, Williamsport, PA, USA) at 0 °C. Thermocouples were inserted approximately 2 cm into the soil of six different cells, and the temperatures recorded (Omega RDXL12SD) (Supplementary Materials Figure S1). Once the soil equilibrated to 0 °C, a slurry of INA bacteria was sprayed onto the plants to ensure freezing would occur [39]. The following freezing program was then enacted: −1 °C/h to −6 °C, 2 h soak at −6 °C, +1 C/h to 0 °C, soak for 4 h, +2 °C/h to + 5 °C, hold. The cell packs were then removed and returned to standard growth conditions. The plants were then scored as living/dead one week later, with photographs taken. Upwards of 72 plants were assessed per freezing test (60 transgenic lines and 12 untransformed C0 plants). Lines that survived these stringent conditions were allowed to flower, seed-collected, and the lines retested.

## 5. Conclusions

In the current study, a novel resource for the analysis of gene function and diversity in *M. sieversii*, a wild apple species recognized as a progenitor of the domestic apple (*M. pumila*), was created using the FOX hunting system [25]. *M. sieversii* represents an important source of genetic diversity and an important reservoir for disease and stress resistance genes, as well as fruit quality traits (taste and aroma). The *M. sieversii* FOX library provides a novel resource that can be used to study apple gene function and genetic diversity, and for the ad hoc identification of physiologically relevant genes through high-throughput screening. This resource is freely available upon request through a Materials Transfer Agreement with the USDA-ARS. The FL-cDNA library and apple FOX seed bank also provide opportunities for the genetic improvement of domesticated apple cultivars through gene editing or more traditional gene transformation approaches. BLAST analysis of the inserts against the doubled haploid “Golden Delicious” genome or NCBI have revealed numerous genes involved with metabolism, abiotic and biotic stress resistance, and dormancy. The resource provides a foundation for developing a better understanding of economically important traits in apple.

## Figures and Tables

**Table 1:** GDDH accessions and gene descriptions of lines depicted in Figure 3.

FOX ID	GDDH Accession	GDDH Description
145	MD01G1079300	P-loop_containing NTP_hydrolases_superfamily_
167	MD09G1209600	RING-H2_finger_C1A
186	MD15G1163400	RING/U-box_superfamily_protein
216	MD10G1078600	mediator_of_RNA_polymerase_II_transcription_subunit_32
236	MD00G1140600	HIT_zinc_finger
263	MD11G1241900	Aluminium_induced_protein_with_YGL_and_LRDR_motifs

GDDH, “Golden Delicious” doubled haploid apple genome [13,15].

**Table 2:** Annotated list of apple FOX Arabidopsis lines that survived the initial freezing tests.

FOX ID	% Control	% Line	GDDH Accession	GDDH Description
124	30	44.7	MD07G1138200	beta-amylase_7
159	26	36	MD13G1087000	Putative_thiol-disulphide_oxidoreductase_DCC
185	6.66	5.33	MD17G1034100	DegP_protease_1
697	0	9.33	MD17G1116400	unknown
797	0	0	MD11G1120700	Protein_kinase_superfamily_protein
133	3.33	12	MD15G1293600	casein_kinase_alpha_1
134	13.33	26.76	MD15G1293600	casein_kinase_alpha_1
1847	0	1.3	MD15G1003900	cold-regulated_47

## Supplementary Materials

Table S1. Apple FOX Arabidopsis lines with sequenced inserts arranged in numerical order. Gaps in the order represent lines that were found to have multiple inserts or were lethal. Table S2. Apple FOX Arabidopsis lines with sequenced inserts arranged by “Golden Delicious” Doubled Haploid (GDDH) description. Similar GDDH descriptions are grouped together. Different GDDH accessions may be found in the same group. Not included are poor quality reads or reads apparently matching non-Rosaceae sequences (such as bacteria or human genomes). Table S3. KEGG GO pathways found in the sequenced apple FOX Arabidopsis lines. Table S4. Apple FOX Arabidopsis lines with sequenced inserts related to abiotic stress responses. Table S5. Apple FOX Arabidopsis lines with sequenced inserts related to biotic stress responses. Table S6. Apple FOX Arabidopsis lines with sequenced inserts related to dormancy. Figure S1. Thermocouple inserts to monitor cell-pack soil temperature. (a) Wide view. (b) Close-up. Supplementary Materials can be found at https://www.mdpi.com/1422-0067/21/24/9516/s1.
