# Supplementary material for: Fox Hunting in Wild Apples: Searching for Novel Genes in Malus Sieversii
Source: Int J Mol Sci. 2020 Dec 14;21(24):9516. doi: 10.3390/ijms21249516 (PMC7765095; doi:10.3390/ijms21249516)
Supplement: Supplementary file 1 [file ijms-21-09516-s001.zip › Supplemental Table 3.docx]

| Pathway # Pathway Description Number of genes in pathway |
| --- |
| [mdm01100](https://www.kegg.jp/kegg-bin/show_pathway?157443610656263/mdm01100.args) Metabolic pathways - Malus domestica (apple) ([182](javascript:display('mdm01100'))) |
|  |
| [mdm:103400579](https://www.kegg.jp/dbget-bin/www_bget?mdm:103400579) 3-hydroxyisobutyryl-CoA hydrolase-like protein 3, mitochondrial |
| [mdm:103400922](https://www.kegg.jp/dbget-bin/www_bget?mdm:103400922) MF-60; aldehyde dehydrogenase family 7 member A1 |
| [mdm:103401040](https://www.kegg.jp/dbget-bin/www_bget?mdm:103401040) probable prolyl 4-hydroxylase 10 |
| [mdm:103401300](https://www.kegg.jp/dbget-bin/www_bget?mdm:103401300) methylthioribose kinase-like |
| [mdm:103401356](https://www.kegg.jp/dbget-bin/www_bget?mdm:103401356) putative glucose-6-phosphate 1-epimerase |
| [mdm:103401538](https://www.kegg.jp/dbget-bin/www_bget?mdm:103401538) uncharacterized protein LOC103401538 |
| [mdm:103401714](https://www.kegg.jp/dbget-bin/www_bget?mdm:103401714) aldehyde dehydrogenase family 3 member H1-like isoform X2 |
| [mdm:103401914](https://www.kegg.jp/dbget-bin/www_bget?mdm:103401914) beta-amylase 7 isoform X1 |
| [mdm:103402280](https://www.kegg.jp/dbget-bin/www_bget?mdm:103402280) probable N-acetyl-gamma-glutamyl-phosphate reductase, chloroplastic |
| [mdm:103402343](https://www.kegg.jp/dbget-bin/www_bget?mdm:103402343) cytochrome b-c1 complex subunit 6-like |
| [mdm:103402794](https://www.kegg.jp/dbget-bin/www_bget?mdm:103402794) S-adenosylmethionine decarboxylase proenzyme-like |
| [mdm:103403012](https://www.kegg.jp/dbget-bin/www_bget?mdm:103403012) carbonic anhydrase 2-like |
| [mdm:103403033](https://www.kegg.jp/dbget-bin/www_bget?mdm:103403033) ATP-citrate synthase alpha chain protein 1 |
| [mdm:103403075](https://www.kegg.jp/dbget-bin/www_bget?mdm:103403075) glutamate--glyoxylate aminotransferase 2 isoform X1 |
| [mdm:103403337](https://www.kegg.jp/dbget-bin/www_bget?mdm:103403337) vinorine synthase |
| [mdm:103403391](https://www.kegg.jp/dbget-bin/www_bget?mdm:103403391) FAD synthetase 2, chloroplastic-like isoform X2 |
| [mdm:103403601](https://www.kegg.jp/dbget-bin/www_bget?mdm:103403601) 3-isopropylmalate dehydratase large subunit, chloroplastic |
| [mdm:103404032](https://www.kegg.jp/dbget-bin/www_bget?mdm:103404032) adenylyl-sulfate kinase 3 isoform X1 |
| [mdm:103404202](https://www.kegg.jp/dbget-bin/www_bget?mdm:103404202) uridine kinase-like protein 1, chloroplastic |
| [mdm:103404212](https://www.kegg.jp/dbget-bin/www_bget?mdm:103404212) ribulose-phosphate 3-epimerase, cytoplasmic isoform-like |
| [mdm:103404221](https://www.kegg.jp/dbget-bin/www_bget?mdm:103404221) thymidylate kinase isoform X1 |
| [mdm:103404264](https://www.kegg.jp/dbget-bin/www_bget?mdm:103404264) putative glucose-6-phosphate 1-epimerase isoform X1 |
| [mdm:103404478](https://www.kegg.jp/dbget-bin/www_bget?mdm:103404478) chorismate mutase 1, chloroplastic |
| [mdm:103404567](https://www.kegg.jp/dbget-bin/www_bget?mdm:103404567) sirohydrochlorin ferrochelatase, chloroplastic |
| [mdm:103405001](https://www.kegg.jp/dbget-bin/www_bget?mdm:103405001) chlorophyll a-b binding protein 151, chloroplastic-like |
| [mdm:103405614](https://www.kegg.jp/dbget-bin/www_bget?mdm:103405614) 4-alpha-glucanotransferase, chloroplastic/amyloplastic |
| [mdm:103405636](https://www.kegg.jp/dbget-bin/www_bget?mdm:103405636) tyrosine aminotransferase-like |
| [mdm:103406415](https://www.kegg.jp/dbget-bin/www_bget?mdm:103406415) phosphoinositide phospholipase C 2-like |
| [mdm:103407292](https://www.kegg.jp/dbget-bin/www_bget?mdm:103407292) primary amine oxidase-like |
| [mdm:103408835](https://www.kegg.jp/dbget-bin/www_bget?mdm:103408835) PAP-specific phosphatase HAL2-like |
| [mdm:103408973](https://www.kegg.jp/dbget-bin/www_bget?mdm:103408973) pyruvate dehydrogenase E1 component subunit beta-3, chloroplastic-like |
| [mdm:103409471](https://www.kegg.jp/dbget-bin/www_bget?mdm:103409471) 5'-adenylylsulfate reductase 3, chloroplastic-like |
| [mdm:103409732](https://www.kegg.jp/dbget-bin/www_bget?mdm:103409732) betaine aldehyde dehydrogenase 1, chloroplastic |
| [mdm:103410608](https://www.kegg.jp/dbget-bin/www_bget?mdm:103410608) L-ascorbate peroxidase 3-like |
| [mdm:103410737](https://www.kegg.jp/dbget-bin/www_bget?mdm:103410737) ATP sulfurylase 1, chloroplastic |
| [mdm:103411515](https://www.kegg.jp/dbget-bin/www_bget?mdm:103411515) long chain acyl-CoA synthetase 6, peroxisomal-like isoform X1 |
| [mdm:103411553](https://www.kegg.jp/dbget-bin/www_bget?mdm:103411553) LOW QUALITY PROTEIN: cycloartenol synthase-like |
| [mdm:103413165](https://www.kegg.jp/dbget-bin/www_bget?mdm:103413165) uncharacterized protein LOC103413165 |
| [mdm:103414581](https://www.kegg.jp/dbget-bin/www_bget?mdm:103414581) NADH dehydrogenase [ubiquinone] iron-sulfur protein 8-A, mitochondrial |
| [mdm:103414619](https://www.kegg.jp/dbget-bin/www_bget?mdm:103414619) phosphoinositide phosphatase SAC8-like |
| [mdm:103414953](https://www.kegg.jp/dbget-bin/www_bget?mdm:103414953) digalactosyldiacylglycerol synthase 1, chloroplastic-like |
| [mdm:103415187](https://www.kegg.jp/dbget-bin/www_bget?mdm:103415187) probable pectate lyase 18 |
| [mdm:103415799](https://www.kegg.jp/dbget-bin/www_bget?mdm:103415799) dCTP pyrophosphatase 1-like |
| [mdm:103416043](https://www.kegg.jp/dbget-bin/www_bget?mdm:103416043) histidinol-phosphate aminotransferase, chloroplastic-like |
| [mdm:103416907](https://www.kegg.jp/dbget-bin/www_bget?mdm:103416907) cycloartenol synthase 2 |
| [mdm:103416934](https://www.kegg.jp/dbget-bin/www_bget?mdm:103416934) cytochrome b-c1 complex subunit Rieske-4, mitochondrial-like |
| [mdm:103416945](https://www.kegg.jp/dbget-bin/www_bget?mdm:103416945) cytochrome c1-2, heme protein, mitochondrial |
| [mdm:103417231](https://www.kegg.jp/dbget-bin/www_bget?mdm:103417231) V-type proton ATPase subunit D-like |
| [mdm:103418639](https://www.kegg.jp/dbget-bin/www_bget?mdm:103418639) 6-phosphofructo-2-kinase/fructose-2, 6-bisphosphatase |
| [mdm:103418786](https://www.kegg.jp/dbget-bin/www_bget?mdm:103418786) uncharacterized protein LOC103418786 |
| [mdm:103419429](https://www.kegg.jp/dbget-bin/www_bget?mdm:103419429) uridine kinase-like protein 3 |
| [mdm:103419872](https://www.kegg.jp/dbget-bin/www_bget?mdm:103419872) putative glucose-6-phosphate 1-epimerase |
| [mdm:103420099](https://www.kegg.jp/dbget-bin/www_bget?mdm:103420099) cinnamyl alcohol dehydrogenase 1 |
| [mdm:103420331](https://www.kegg.jp/dbget-bin/www_bget?mdm:103420331) delta-1-pyrroline-5-carboxylate dehydrogenase 12A1, mitochondrial-like |
| [mdm:103420449](https://www.kegg.jp/dbget-bin/www_bget?mdm:103420449) ATP synthase subunit O, mitochondrial-like |
| [mdm:103420529](https://www.kegg.jp/dbget-bin/www_bget?mdm:103420529) (+)-neomenthol dehydrogenase-like |
| [mdm:103420733](https://www.kegg.jp/dbget-bin/www_bget?mdm:103420733) 4-hydroxy-3-methylbut-2-enyl diphosphate reductase, chloroplastic |
| [mdm:103422058](https://www.kegg.jp/dbget-bin/www_bget?mdm:103422058) peroxidase 12-like |
| [mdm:103422468](https://www.kegg.jp/dbget-bin/www_bget?mdm:103422468) peroxidase 42-like |
| [mdm:103422491](https://www.kegg.jp/dbget-bin/www_bget?mdm:103422491) inositol-phosphate phosphatase-like |
| [mdm:103422717](https://www.kegg.jp/dbget-bin/www_bget?mdm:103422717) LOW QUALITY PROTEIN: tropinone reductase homolog At2g29330-like |
| [mdm:103423287](https://www.kegg.jp/dbget-bin/www_bget?mdm:103423287) pyrroline-5-carboxylate reductase-like |
| [mdm:103423404](https://www.kegg.jp/dbget-bin/www_bget?mdm:103423404) glutathione S-transferase L3-like |
| [mdm:103424543](https://www.kegg.jp/dbget-bin/www_bget?mdm:103424543) malate dehydrogenase, chloroplastic-like |
| [mdm:103425450](https://www.kegg.jp/dbget-bin/www_bget?mdm:103425450) protein PHYLLO, chloroplastic isoform X1 |
| [mdm:103425825](https://www.kegg.jp/dbget-bin/www_bget?mdm:103425825) mannose-1-phosphate guanyltransferase alpha-like |
| [mdm:103426071](https://www.kegg.jp/dbget-bin/www_bget?mdm:103426071) probable GTP diphosphokinase RSH2, chloroplastic |
| [mdm:103426493](https://www.kegg.jp/dbget-bin/www_bget?mdm:103426493) acidic endochitinase-like |
| [mdm:103428701](https://www.kegg.jp/dbget-bin/www_bget?mdm:103428701) probable polygalacturonase At1g80170 |
| [mdm:103429189](https://www.kegg.jp/dbget-bin/www_bget?mdm:103429189) glucose-6-phosphate 1-dehydrogenase, chloroplastic-like |
| [mdm:103429192](https://www.kegg.jp/dbget-bin/www_bget?mdm:103429192) acetyl-CoA acetyltransferase, cytosolic 1 |
| [mdm:103429307](https://www.kegg.jp/dbget-bin/www_bget?mdm:103429307) serine racemase isoform X1 |
| [mdm:103429705](https://www.kegg.jp/dbget-bin/www_bget?mdm:103429705) adenosine kinase 2 |
| [mdm:103430865](https://www.kegg.jp/dbget-bin/www_bget?mdm:103430865) cyanate hydratase |
| [mdm:103431468](https://www.kegg.jp/dbget-bin/www_bget?mdm:103431468) beta-1,4-mannosyl-glycoprotein 4-beta-N-acetylglucosaminyltransferase |
| [mdm:103432577](https://www.kegg.jp/dbget-bin/www_bget?mdm:103432577) thiamine pyrophosphokinase 1-like isoform X1 |
| [mdm:103432613](https://www.kegg.jp/dbget-bin/www_bget?mdm:103432613) putative GTP diphosphokinase RSH1, chloroplastic |
| [mdm:103433463](https://www.kegg.jp/dbget-bin/www_bget?mdm:103433463) DHAR; glutathione S-transferase DHAR2-like |
| [mdm:103433738](https://www.kegg.jp/dbget-bin/www_bget?mdm:103433738) cationic peroxidase 1-like |
| [mdm:103434138](https://www.kegg.jp/dbget-bin/www_bget?mdm:103434138) cysteine synthase-like isoform X1 |
| [mdm:103434186](https://www.kegg.jp/dbget-bin/www_bget?mdm:103434186) phosphoribosylaminoimidazole carboxylase, chloroplastic-like isoform X1 |
| [mdm:103434248](https://www.kegg.jp/dbget-bin/www_bget?mdm:103434248) probable UDP-arabinopyranose mutase 5 isoform X1 |
| [mdm:103434495](https://www.kegg.jp/dbget-bin/www_bget?mdm:103434495) ATP synthase gamma chain, chloroplastic-like |
| [mdm:103434574](https://www.kegg.jp/dbget-bin/www_bget?mdm:103434574) beta-hexosaminidase 1-like |
| [mdm:103434638](https://www.kegg.jp/dbget-bin/www_bget?mdm:103434638) anthocyanidin reductase |
| [mdm:103435112](https://www.kegg.jp/dbget-bin/www_bget?mdm:103435112) acetolactate synthase small subunit 1, chloroplastic-like |
| [mdm:103435398](https://www.kegg.jp/dbget-bin/www_bget?mdm:103435398) thiosulfate/3-mercaptopyruvate sulfurtransferase 1, mitochondrial-like |
| [mdm:103436074](https://www.kegg.jp/dbget-bin/www_bget?mdm:103436074) NADH dehydrogenase [ubiquinone] flavoprotein 2, mitochondrial-like |
| [mdm:103436360](https://www.kegg.jp/dbget-bin/www_bget?mdm:103436360) phospho-2-dehydro-3-deoxyheptonate aldolase 1, chloroplastic-like |
| [mdm:103436439](https://www.kegg.jp/dbget-bin/www_bget?mdm:103436439) D-3-phosphoglycerate dehydrogenase 1, chloroplastic-like |
| [mdm:103436467](https://www.kegg.jp/dbget-bin/www_bget?mdm:103436467) LL-diaminopimelate aminotransferase, chloroplastic-like |
| [mdm:103436482](https://www.kegg.jp/dbget-bin/www_bget?mdm:103436482) methylcrotonoyl-CoA carboxylase beta chain, mitochondrial |
| [mdm:103436725](https://www.kegg.jp/dbget-bin/www_bget?mdm:103436725) cysteine synthase-like |
| [mdm:103437260](https://www.kegg.jp/dbget-bin/www_bget?mdm:103437260) phospholipase D alpha 1 |
| [mdm:103437705](https://www.kegg.jp/dbget-bin/www_bget?mdm:103437705) anthranilate phosphoribosyltransferase, chloroplastic |
| [mdm:103437782](https://www.kegg.jp/dbget-bin/www_bget?mdm:103437782) 5-formyltetrahydrofolate cyclo-ligase, mitochondrial-like |
| [mdm:103438028](https://www.kegg.jp/dbget-bin/www_bget?mdm:103438028) uncharacterized protein LOC103438028 |
| [mdm:103438425](https://www.kegg.jp/dbget-bin/www_bget?mdm:103438425) polyamine oxidase 1 |
| [mdm:103438582](https://www.kegg.jp/dbget-bin/www_bget?mdm:103438582) hydroxyacylglutathione hydrolase cytoplasmic |
| [mdm:103438644](https://www.kegg.jp/dbget-bin/www_bget?mdm:103438644) histone-lysine N-methyltransferase, H3 lysine-9 specific SUVH1-like |
| [mdm:103438670](https://www.kegg.jp/dbget-bin/www_bget?mdm:103438670) LOW QUALITY PROTEIN: inositol polyphosphate multikinase beta-like |
| [mdm:103439436](https://www.kegg.jp/dbget-bin/www_bget?mdm:103439436) granule-bound starch synthase 1, chloroplastic/amyloplastic-like |
| [mdm:103439769](https://www.kegg.jp/dbget-bin/www_bget?mdm:103439769) probable prolyl 4-hydroxylase 12 isoform X1 |
| [mdm:103440430](https://www.kegg.jp/dbget-bin/www_bget?mdm:103440430) glucose-6-phosphate isomerase, cytosolic |
| [mdm:103440661](https://www.kegg.jp/dbget-bin/www_bget?mdm:103440661) LOW QUALITY PROTEIN: dolichyl-diphosphooligosaccharide--protein glycosyltransferase subunit 1B-like |
| [mdm:103441092](https://www.kegg.jp/dbget-bin/www_bget?mdm:103441092) long chain base biosynthesis protein 1-like isoform X1 |
| [mdm:103441178](https://www.kegg.jp/dbget-bin/www_bget?mdm:103441178) aspartate aminotransferase, cytoplasmic |
| [mdm:103441205](https://www.kegg.jp/dbget-bin/www_bget?mdm:103441205) probable fructokinase-6, chloroplastic |
| [mdm:103441211](https://www.kegg.jp/dbget-bin/www_bget?mdm:103441211) gamma-glutamylcyclotransferase 2-1-like |
| [mdm:103441571](https://www.kegg.jp/dbget-bin/www_bget?mdm:103441571) ent-kaurene oxidase, chloroplastic-like |
| [mdm:103441898](https://www.kegg.jp/dbget-bin/www_bget?mdm:103441898) phosphatidylinositol 4-phosphate 5-kinase 8-like isoform X1 |
| [mdm:103442089](https://www.kegg.jp/dbget-bin/www_bget?mdm:103442089) V-type proton ATPase subunit C |
| [mdm:103442605](https://www.kegg.jp/dbget-bin/www_bget?mdm:103442605) aconitate hydratase, cytoplasmic |
| [mdm:103442704](https://www.kegg.jp/dbget-bin/www_bget?mdm:103442704) histone-lysine N-methyltransferase, H3 lysine-9 specific SUVH4 |
| [mdm:103442796](https://www.kegg.jp/dbget-bin/www_bget?mdm:103442796) V-type proton ATPase subunit d2 |
| [mdm:103443458](https://www.kegg.jp/dbget-bin/www_bget?mdm:103443458) copper methylamine oxidase-like |
| [mdm:103443512](https://www.kegg.jp/dbget-bin/www_bget?mdm:103443512) polyketide synthase 5-like |
| [mdm:103443644](https://www.kegg.jp/dbget-bin/www_bget?mdm:103443644) phosphoinositide phosphatase SAC2-like isoform X1 |
| [mdm:103444197](https://www.kegg.jp/dbget-bin/www_bget?mdm:103444197) glutathione S-transferase DHAR3, chloroplastic |
| [mdm:103444873](https://www.kegg.jp/dbget-bin/www_bget?mdm:103444873) uncharacterized protein LOC103444873 isoform X1 |
| [mdm:103444921](https://www.kegg.jp/dbget-bin/www_bget?mdm:103444921) inositol-tetrakisphosphate 1-kinase 1-like |
| [mdm:103445180](https://www.kegg.jp/dbget-bin/www_bget?mdm:103445180) LOW QUALITY PROTEIN: LL-diaminopimelate aminotransferase, chloroplastic-like |
| [mdm:103445713](https://www.kegg.jp/dbget-bin/www_bget?mdm:103445713) tryptophan synthase alpha chain-like |
| [mdm:103445750](https://www.kegg.jp/dbget-bin/www_bget?mdm:103445750) peroxisomal (S)-2-hydroxy-acid oxidase-like |
| [mdm:103445864](https://www.kegg.jp/dbget-bin/www_bget?mdm:103445864) thiosulfate/3-mercaptopyruvate sulfurtransferase 1, mitochondrial-like |
| [mdm:103446060](https://www.kegg.jp/dbget-bin/www_bget?mdm:103446060) digalactosyldiacylglycerol synthase 2, chloroplastic isoform X2 |
| [mdm:103446228](https://www.kegg.jp/dbget-bin/www_bget?mdm:103446228) glycerol-3-phosphate acyltransferase 9 |
| [mdm:103446341](https://www.kegg.jp/dbget-bin/www_bget?mdm:103446341) magnesium-chelatase subunit ChlI, chloroplastic |
| [mdm:103446625](https://www.kegg.jp/dbget-bin/www_bget?mdm:103446625) probable phospholipid hydroperoxide glutathione peroxidase |
| [mdm:103446628](https://www.kegg.jp/dbget-bin/www_bget?mdm:103446628) probable phospholipid hydroperoxide glutathione peroxidase |
| [mdm:103446691](https://www.kegg.jp/dbget-bin/www_bget?mdm:103446691) beta-glucosidase 46-like |
| [mdm:103447094](https://www.kegg.jp/dbget-bin/www_bget?mdm:103447094) NADP-dependent glyceraldehyde-3-phosphate dehydrogenase isoform X2 |
| [mdm:103447435](https://www.kegg.jp/dbget-bin/www_bget?mdm:103447435) LOW QUALITY PROTEIN: phosphomethylpyrimidine synthase, chloroplastic |
| [mdm:103448063](https://www.kegg.jp/dbget-bin/www_bget?mdm:103448063) lactoylglutathione lyase isoform X1 |
| [mdm:103448267](https://www.kegg.jp/dbget-bin/www_bget?mdm:103448267) caffeoyl-CoA O-methyltransferase |
| [mdm:103448970](https://www.kegg.jp/dbget-bin/www_bget?mdm:103448970) methylcrotonoyl-CoA carboxylase subunit alpha, mitochondrial |
| [mdm:103449161](https://www.kegg.jp/dbget-bin/www_bget?mdm:103449161) putative GTP diphosphokinase RSH1, chloroplastic |
| [mdm:103449400](https://www.kegg.jp/dbget-bin/www_bget?mdm:103449400) fructose-bisphosphate aldolase 6, cytosolic |
| [mdm:103449888](https://www.kegg.jp/dbget-bin/www_bget?mdm:103449888) hexokinase-1-like |
| [mdm:103450322](https://www.kegg.jp/dbget-bin/www_bget?mdm:103450322) 4-coumarate--CoA ligase-like 5 |
| [mdm:103450546](https://www.kegg.jp/dbget-bin/www_bget?mdm:103450546) V-type proton ATPase 16 kDa proteolipid subunit |
| [mdm:103450812](https://www.kegg.jp/dbget-bin/www_bget?mdm:103450812) probable L-cysteine desulfhydrase, chloroplastic |
| [mdm:103450933](https://www.kegg.jp/dbget-bin/www_bget?mdm:103450933) diaminopimelate epimerase, chloroplastic-like isoform X1 |
| [mdm:103451064](https://www.kegg.jp/dbget-bin/www_bget?mdm:103451064) probable low-specificity L-threonine aldolase 1 |
| [mdm:103451755](https://www.kegg.jp/dbget-bin/www_bget?mdm:103451755) probable 6-phosphogluconolactonase 1 |
| [mdm:103451863](https://www.kegg.jp/dbget-bin/www_bget?mdm:103451863) AMY10; alpha-amylase 3, chloroplastic-like |
| [mdm:103451886](https://www.kegg.jp/dbget-bin/www_bget?mdm:103451886) glutathione S-transferase U17-like |
| [mdm:103452038](https://www.kegg.jp/dbget-bin/www_bget?mdm:103452038) copper methylamine oxidase-like |
| [mdm:103452041](https://www.kegg.jp/dbget-bin/www_bget?mdm:103452041) glutamate--glyoxylate aminotransferase 2-like |
| [mdm:103452065](https://www.kegg.jp/dbget-bin/www_bget?mdm:103452065) glutamate dehydrogenase 1-like |
| [mdm:103452551](https://www.kegg.jp/dbget-bin/www_bget?mdm:103452551) methionine gamma-lyase-like |
| [mdm:103452636](https://www.kegg.jp/dbget-bin/www_bget?mdm:103452636) ATP-citrate synthase alpha chain protein 2 isoform X1 |
| [mdm:103452756](https://www.kegg.jp/dbget-bin/www_bget?mdm:103452756) adenylosuccinate synthetase 2, chloroplastic-like |
| [mdm:103453083](https://www.kegg.jp/dbget-bin/www_bget?mdm:103453083) 3-dehydrosphinganine reductase TSC10A |
| [mdm:103453644](https://www.kegg.jp/dbget-bin/www_bget?mdm:103453644) LOW QUALITY PROTEIN: biotin carboxylase 1, chloroplastic |
| [mdm:103453790](https://www.kegg.jp/dbget-bin/www_bget?mdm:103453790) fructose-bisphosphate aldolase 1, chloroplastic |
| [mdm:103453924](https://www.kegg.jp/dbget-bin/www_bget?mdm:103453924) enolase |
| [mdm:103454483](https://www.kegg.jp/dbget-bin/www_bget?mdm:103454483) S-formylglutathione hydrolase isoform X1 |
| [mdm:103454693](https://www.kegg.jp/dbget-bin/www_bget?mdm:103454693) L-2-hydroxyglutarate dehydrogenase, mitochondrial-like isoform X1 |
| [mdm:103454765](https://www.kegg.jp/dbget-bin/www_bget?mdm:103454765) shikimate kinase, chloroplastic-like |
| [mdm:103455216](https://www.kegg.jp/dbget-bin/www_bget?mdm:103455216) pantothenate kinase 2 |
| [mdm:103455413](https://www.kegg.jp/dbget-bin/www_bget?mdm:103455413) flavonoid 3'-monooxygenase |
| [mdm:103455658](https://www.kegg.jp/dbget-bin/www_bget?mdm:103455658) putative 3,4-dihydroxy-2-butanone kinase |
| [mdm:103455699](https://www.kegg.jp/dbget-bin/www_bget?mdm:103455699) succinate--CoA ligase [ADP-forming] subunit alpha-2, mitochondrial-like |
| [mdm:103455898](https://www.kegg.jp/dbget-bin/www_bget?mdm:103455898) probable prolyl 4-hydroxylase 10 |
| [mdm:103456183](https://www.kegg.jp/dbget-bin/www_bget?mdm:103456183) calmodulin-lysine N-methyltransferase isoform X2 |
| [mdm:103456287](https://www.kegg.jp/dbget-bin/www_bget?mdm:103456287) magnesium-chelatase subunit ChlH, chloroplastic |
| [mdm:114819149](https://www.kegg.jp/dbget-bin/www_bget?mdm:114819149) xanthoxin dehydrogenase-like |
| [mdm:114820473](https://www.kegg.jp/dbget-bin/www_bget?mdm:114820473) isoamylase 1, chloroplastic |
| [mdm:114822544](https://www.kegg.jp/dbget-bin/www_bget?mdm:114822544) NADP-dependent malic enzyme-like isoform X1 |
| [mdm:114822925](https://www.kegg.jp/dbget-bin/www_bget?mdm:114822925) NADH dehydrogenase [ubiquinone] 1 beta subcomplex subunit 9-like |
| [mdm:114823197](https://www.kegg.jp/dbget-bin/www_bget?mdm:114823197) fructose-bisphosphate aldolase 1, chloroplastic-like |
| [mdm:114824344](https://www.kegg.jp/dbget-bin/www_bget?mdm:114824344) LOW QUALITY PROTEIN: glutamate dehydrogenase 2-like |
| [mdm:114824453](https://www.kegg.jp/dbget-bin/www_bget?mdm:114824453) phosphoglycolate phosphatase 1B, chloroplastic-like |
| [mdm:114824777](https://www.kegg.jp/dbget-bin/www_bget?mdm:114824777) squalene synthase-like |
| [mdm:114825363](https://www.kegg.jp/dbget-bin/www_bget?mdm:114825363) glyceraldehyde-3-phosphate dehydrogenase, cytosolic-like |
| [mdm:114825652](https://www.kegg.jp/dbget-bin/www_bget?mdm:114825652) puromycin-sensitive aminopeptidase-like isoform X1 |
| [mdm:114826512](https://www.kegg.jp/dbget-bin/www_bget?mdm:114826512) glutamyl-tRNA(Gln) amidotransferase subunit C, chloroplastic/mitochondrial |
| [mdm:114826950](https://www.kegg.jp/dbget-bin/www_bget?mdm:114826950) dihydrolipoyllysine-residue succinyltransferase component of 2-oxoglutarate dehydrogenase complex 1, mitochondrial-like |
| [mdm:114826994](https://www.kegg.jp/dbget-bin/www_bget?mdm:114826994) peroxidase 17-like |
| [mdm:114827525](https://www.kegg.jp/dbget-bin/www_bget?mdm:114827525) putative 1-phosphatidylinositol-3-phosphate 5-kinase FAB1D |
| [mdm:114827740](https://www.kegg.jp/dbget-bin/www_bget?mdm:114827740) lecithin-cholesterol acyltransferase-like 4 |
| [mdm01110](https://www.kegg.jp/kegg-bin/show_pathway?157443610656263/mdm01110.args) Biosynthesis of secondary metabolites - Malus domestica (apple) ([86](javascript:display('mdm01110'))) |
|  |
| [mdm:103400922](https://www.kegg.jp/dbget-bin/www_bget?mdm:103400922) MF-60; aldehyde dehydrogenase family 7 member A1 |
| [mdm:103401356](https://www.kegg.jp/dbget-bin/www_bget?mdm:103401356) putative glucose-6-phosphate 1-epimerase |
| [mdm:103401375](https://www.kegg.jp/dbget-bin/www_bget?mdm:103401375) red chlorophyll catabolite reductase, chloroplastic |
| [mdm:103401714](https://www.kegg.jp/dbget-bin/www_bget?mdm:103401714) aldehyde dehydrogenase family 3 member H1-like isoform X2 |
| [mdm:103402280](https://www.kegg.jp/dbget-bin/www_bget?mdm:103402280) probable N-acetyl-gamma-glutamyl-phosphate reductase, chloroplastic |
| [mdm:103403033](https://www.kegg.jp/dbget-bin/www_bget?mdm:103403033) ATP-citrate synthase alpha chain protein 1 |
| [mdm:103403075](https://www.kegg.jp/dbget-bin/www_bget?mdm:103403075) glutamate--glyoxylate aminotransferase 2 isoform X1 |
| [mdm:103403337](https://www.kegg.jp/dbget-bin/www_bget?mdm:103403337) vinorine synthase |
| [mdm:103403391](https://www.kegg.jp/dbget-bin/www_bget?mdm:103403391) FAD synthetase 2, chloroplastic-like isoform X2 |
| [mdm:103403601](https://www.kegg.jp/dbget-bin/www_bget?mdm:103403601) 3-isopropylmalate dehydratase large subunit, chloroplastic |
| [mdm:103404212](https://www.kegg.jp/dbget-bin/www_bget?mdm:103404212) ribulose-phosphate 3-epimerase, cytoplasmic isoform-like |
| [mdm:103404264](https://www.kegg.jp/dbget-bin/www_bget?mdm:103404264) putative glucose-6-phosphate 1-epimerase isoform X1 |
| [mdm:103404478](https://www.kegg.jp/dbget-bin/www_bget?mdm:103404478) chorismate mutase 1, chloroplastic |
| [mdm:103404567](https://www.kegg.jp/dbget-bin/www_bget?mdm:103404567) sirohydrochlorin ferrochelatase, chloroplastic |
| [mdm:103405614](https://www.kegg.jp/dbget-bin/www_bget?mdm:103405614) 4-alpha-glucanotransferase, chloroplastic/amyloplastic |
| [mdm:103405636](https://www.kegg.jp/dbget-bin/www_bget?mdm:103405636) tyrosine aminotransferase-like |
| [mdm:103407292](https://www.kegg.jp/dbget-bin/www_bget?mdm:103407292) primary amine oxidase-like |
| [mdm:103408973](https://www.kegg.jp/dbget-bin/www_bget?mdm:103408973) pyruvate dehydrogenase E1 component subunit beta-3, chloroplastic-like |
| [mdm:103411553](https://www.kegg.jp/dbget-bin/www_bget?mdm:103411553) LOW QUALITY PROTEIN: cycloartenol synthase-like |
| [mdm:103416043](https://www.kegg.jp/dbget-bin/www_bget?mdm:103416043) histidinol-phosphate aminotransferase, chloroplastic-like |
| [mdm:103416907](https://www.kegg.jp/dbget-bin/www_bget?mdm:103416907) cycloartenol synthase 2 |
| [mdm:103419872](https://www.kegg.jp/dbget-bin/www_bget?mdm:103419872) putative glucose-6-phosphate 1-epimerase |
| [mdm:103420099](https://www.kegg.jp/dbget-bin/www_bget?mdm:103420099) cinnamyl alcohol dehydrogenase 1 |
| [mdm:103420733](https://www.kegg.jp/dbget-bin/www_bget?mdm:103420733) 4-hydroxy-3-methylbut-2-enyl diphosphate reductase, chloroplastic |
| [mdm:103422058](https://www.kegg.jp/dbget-bin/www_bget?mdm:103422058) peroxidase 12-like |
| [mdm:103422468](https://www.kegg.jp/dbget-bin/www_bget?mdm:103422468) peroxidase 42-like |
| [mdm:103422491](https://www.kegg.jp/dbget-bin/www_bget?mdm:103422491) inositol-phosphate phosphatase-like |
| [mdm:103422717](https://www.kegg.jp/dbget-bin/www_bget?mdm:103422717) LOW QUALITY PROTEIN: tropinone reductase homolog At2g29330-like |
| [mdm:103423287](https://www.kegg.jp/dbget-bin/www_bget?mdm:103423287) pyrroline-5-carboxylate reductase-like |
| [mdm:103424543](https://www.kegg.jp/dbget-bin/www_bget?mdm:103424543) malate dehydrogenase, chloroplastic-like |
| [mdm:103425450](https://www.kegg.jp/dbget-bin/www_bget?mdm:103425450) protein PHYLLO, chloroplastic isoform X1 |
| [mdm:103425825](https://www.kegg.jp/dbget-bin/www_bget?mdm:103425825) mannose-1-phosphate guanyltransferase alpha-like |
| [mdm:103429189](https://www.kegg.jp/dbget-bin/www_bget?mdm:103429189) glucose-6-phosphate 1-dehydrogenase, chloroplastic-like |
| [mdm:103429192](https://www.kegg.jp/dbget-bin/www_bget?mdm:103429192) acetyl-CoA acetyltransferase, cytosolic 1 |
| [mdm:103433738](https://www.kegg.jp/dbget-bin/www_bget?mdm:103433738) cationic peroxidase 1-like |
| [mdm:103434138](https://www.kegg.jp/dbget-bin/www_bget?mdm:103434138) cysteine synthase-like isoform X1 |
| [mdm:103434186](https://www.kegg.jp/dbget-bin/www_bget?mdm:103434186) phosphoribosylaminoimidazole carboxylase, chloroplastic-like isoform X1 |
| [mdm:103434638](https://www.kegg.jp/dbget-bin/www_bget?mdm:103434638) anthocyanidin reductase |
| [mdm:103435112](https://www.kegg.jp/dbget-bin/www_bget?mdm:103435112) acetolactate synthase small subunit 1, chloroplastic-like |
| [mdm:103436360](https://www.kegg.jp/dbget-bin/www_bget?mdm:103436360) phospho-2-dehydro-3-deoxyheptonate aldolase 1, chloroplastic-like |
| [mdm:103436467](https://www.kegg.jp/dbget-bin/www_bget?mdm:103436467) LL-diaminopimelate aminotransferase, chloroplastic-like |
| [mdm:103436725](https://www.kegg.jp/dbget-bin/www_bget?mdm:103436725) cysteine synthase-like |
| [mdm:103437260](https://www.kegg.jp/dbget-bin/www_bget?mdm:103437260) phospholipase D alpha 1 |
| [mdm:103437348](https://www.kegg.jp/dbget-bin/www_bget?mdm:103437348) salutaridine reductase-like isoform X2 |
| [mdm:103437705](https://www.kegg.jp/dbget-bin/www_bget?mdm:103437705) anthranilate phosphoribosyltransferase, chloroplastic |
| [mdm:103438384](https://www.kegg.jp/dbget-bin/www_bget?mdm:103438384) UDP-glucose flavonoid 3-O-glucosyltransferase 7-like |
| [mdm:103439436](https://www.kegg.jp/dbget-bin/www_bget?mdm:103439436) granule-bound starch synthase 1, chloroplastic/amyloplastic-like |
| [mdm:103440430](https://www.kegg.jp/dbget-bin/www_bget?mdm:103440430) glucose-6-phosphate isomerase, cytosolic |
| [mdm:103441178](https://www.kegg.jp/dbget-bin/www_bget?mdm:103441178) aspartate aminotransferase, cytoplasmic |
| [mdm:103441571](https://www.kegg.jp/dbget-bin/www_bget?mdm:103441571) ent-kaurene oxidase, chloroplastic-like |
| [mdm:103442605](https://www.kegg.jp/dbget-bin/www_bget?mdm:103442605) aconitate hydratase, cytoplasmic |
| [mdm:103443458](https://www.kegg.jp/dbget-bin/www_bget?mdm:103443458) copper methylamine oxidase-like |
| [mdm:103443512](https://www.kegg.jp/dbget-bin/www_bget?mdm:103443512) polyketide synthase 5-like |
| [mdm:103444873](https://www.kegg.jp/dbget-bin/www_bget?mdm:103444873) uncharacterized protein LOC103444873 isoform X1 |
| [mdm:103445180](https://www.kegg.jp/dbget-bin/www_bget?mdm:103445180) LOW QUALITY PROTEIN: LL-diaminopimelate aminotransferase, chloroplastic-like |
| [mdm:103445713](https://www.kegg.jp/dbget-bin/www_bget?mdm:103445713) tryptophan synthase alpha chain-like |
| [mdm:103445750](https://www.kegg.jp/dbget-bin/www_bget?mdm:103445750) peroxisomal (S)-2-hydroxy-acid oxidase-like |
| [mdm:103446228](https://www.kegg.jp/dbget-bin/www_bget?mdm:103446228) glycerol-3-phosphate acyltransferase 9 |
| [mdm:103446341](https://www.kegg.jp/dbget-bin/www_bget?mdm:103446341) magnesium-chelatase subunit ChlI, chloroplastic |
| [mdm:103446691](https://www.kegg.jp/dbget-bin/www_bget?mdm:103446691) beta-glucosidase 46-like |
| [mdm:103448267](https://www.kegg.jp/dbget-bin/www_bget?mdm:103448267) caffeoyl-CoA O-methyltransferase |
| [mdm:103449400](https://www.kegg.jp/dbget-bin/www_bget?mdm:103449400) fructose-bisphosphate aldolase 6, cytosolic |
| [mdm:103449888](https://www.kegg.jp/dbget-bin/www_bget?mdm:103449888) hexokinase-1-like |
| [mdm:103450322](https://www.kegg.jp/dbget-bin/www_bget?mdm:103450322) 4-coumarate--CoA ligase-like 5 |
| [mdm:103450933](https://www.kegg.jp/dbget-bin/www_bget?mdm:103450933) diaminopimelate epimerase, chloroplastic-like isoform X1 |
| [mdm:103451064](https://www.kegg.jp/dbget-bin/www_bget?mdm:103451064) probable low-specificity L-threonine aldolase 1 |
| [mdm:103451755](https://www.kegg.jp/dbget-bin/www_bget?mdm:103451755) probable 6-phosphogluconolactonase 1 |
| [mdm:103452038](https://www.kegg.jp/dbget-bin/www_bget?mdm:103452038) copper methylamine oxidase-like |
| [mdm:103452041](https://www.kegg.jp/dbget-bin/www_bget?mdm:103452041) glutamate--glyoxylate aminotransferase 2-like |
| [mdm:103452636](https://www.kegg.jp/dbget-bin/www_bget?mdm:103452636) ATP-citrate synthase alpha chain protein 2 isoform X1 |
| [mdm:103453644](https://www.kegg.jp/dbget-bin/www_bget?mdm:103453644) LOW QUALITY PROTEIN: biotin carboxylase 1, chloroplastic |
| [mdm:103453790](https://www.kegg.jp/dbget-bin/www_bget?mdm:103453790) fructose-bisphosphate aldolase 1, chloroplastic |
| [mdm:103453924](https://www.kegg.jp/dbget-bin/www_bget?mdm:103453924) enolase |
| [mdm:103454765](https://www.kegg.jp/dbget-bin/www_bget?mdm:103454765) shikimate kinase, chloroplastic-like |
| [mdm:103455413](https://www.kegg.jp/dbget-bin/www_bget?mdm:103455413) flavonoid 3'-monooxygenase |
| [mdm:103455699](https://www.kegg.jp/dbget-bin/www_bget?mdm:103455699) succinate--CoA ligase [ADP-forming] subunit alpha-2, mitochondrial-like |
| [mdm:103456287](https://www.kegg.jp/dbget-bin/www_bget?mdm:103456287) magnesium-chelatase subunit ChlH, chloroplastic |
| [mdm:114819149](https://www.kegg.jp/dbget-bin/www_bget?mdm:114819149) xanthoxin dehydrogenase-like |
| [mdm:114820473](https://www.kegg.jp/dbget-bin/www_bget?mdm:114820473) isoamylase 1, chloroplastic |
| [mdm:114823197](https://www.kegg.jp/dbget-bin/www_bget?mdm:114823197) fructose-bisphosphate aldolase 1, chloroplastic-like |
| [mdm:114824453](https://www.kegg.jp/dbget-bin/www_bget?mdm:114824453) phosphoglycolate phosphatase 1B, chloroplastic-like |
| [mdm:114824777](https://www.kegg.jp/dbget-bin/www_bget?mdm:114824777) squalene synthase-like |
| [mdm:114825363](https://www.kegg.jp/dbget-bin/www_bget?mdm:114825363) glyceraldehyde-3-phosphate dehydrogenase, cytosolic-like |
| [mdm:114826950](https://www.kegg.jp/dbget-bin/www_bget?mdm:114826950) dihydrolipoyllysine-residue succinyltransferase component of 2-oxoglutarate dehydrogenase complex 1, mitochondrial-like |
| [mdm:114826994](https://www.kegg.jp/dbget-bin/www_bget?mdm:114826994) peroxidase 17-like |
| [mdm:114827740](https://www.kegg.jp/dbget-bin/www_bget?mdm:114827740) lecithin-cholesterol acyltransferase-like 4 |
| [mdm01200](https://www.kegg.jp/kegg-bin/show_pathway?157443610656263/mdm01200.args) Carbon metabolism - Malus domestica (apple) ([33](javascript:display('mdm01200'))) |
|  |
| [mdm:103400579](https://www.kegg.jp/dbget-bin/www_bget?mdm:103400579) 3-hydroxyisobutyryl-CoA hydrolase-like protein 3, mitochondrial |
| [mdm:103401538](https://www.kegg.jp/dbget-bin/www_bget?mdm:103401538) uncharacterized protein LOC103401538 |
| [mdm:103403075](https://www.kegg.jp/dbget-bin/www_bget?mdm:103403075) glutamate--glyoxylate aminotransferase 2 isoform X1 |
| [mdm:103404212](https://www.kegg.jp/dbget-bin/www_bget?mdm:103404212) ribulose-phosphate 3-epimerase, cytoplasmic isoform-like |
| [mdm:103408973](https://www.kegg.jp/dbget-bin/www_bget?mdm:103408973) pyruvate dehydrogenase E1 component subunit beta-3, chloroplastic-like |
| [mdm:103424543](https://www.kegg.jp/dbget-bin/www_bget?mdm:103424543) malate dehydrogenase, chloroplastic-like |
| [mdm:103429189](https://www.kegg.jp/dbget-bin/www_bget?mdm:103429189) glucose-6-phosphate 1-dehydrogenase, chloroplastic-like |
| [mdm:103429192](https://www.kegg.jp/dbget-bin/www_bget?mdm:103429192) acetyl-CoA acetyltransferase, cytosolic 1 |
| [mdm:103434138](https://www.kegg.jp/dbget-bin/www_bget?mdm:103434138) cysteine synthase-like isoform X1 |
| [mdm:103436439](https://www.kegg.jp/dbget-bin/www_bget?mdm:103436439) D-3-phosphoglycerate dehydrogenase 1, chloroplastic-like |
| [mdm:103436725](https://www.kegg.jp/dbget-bin/www_bget?mdm:103436725) cysteine synthase-like |
| [mdm:103440430](https://www.kegg.jp/dbget-bin/www_bget?mdm:103440430) glucose-6-phosphate isomerase, cytosolic |
| [mdm:103441178](https://www.kegg.jp/dbget-bin/www_bget?mdm:103441178) aspartate aminotransferase, cytoplasmic |
| [mdm:103442605](https://www.kegg.jp/dbget-bin/www_bget?mdm:103442605) aconitate hydratase, cytoplasmic |
| [mdm:103445750](https://www.kegg.jp/dbget-bin/www_bget?mdm:103445750) peroxisomal (S)-2-hydroxy-acid oxidase-like |
| [mdm:103447094](https://www.kegg.jp/dbget-bin/www_bget?mdm:103447094) NADP-dependent glyceraldehyde-3-phosphate dehydrogenase isoform X2 |
| [mdm:103449400](https://www.kegg.jp/dbget-bin/www_bget?mdm:103449400) fructose-bisphosphate aldolase 6, cytosolic |
| [mdm:103449888](https://www.kegg.jp/dbget-bin/www_bget?mdm:103449888) hexokinase-1-like |
| [mdm:103451755](https://www.kegg.jp/dbget-bin/www_bget?mdm:103451755) probable 6-phosphogluconolactonase 1 |
| [mdm:103452041](https://www.kegg.jp/dbget-bin/www_bget?mdm:103452041) glutamate--glyoxylate aminotransferase 2-like |
| [mdm:103452065](https://www.kegg.jp/dbget-bin/www_bget?mdm:103452065) glutamate dehydrogenase 1-like |
| [mdm:103453644](https://www.kegg.jp/dbget-bin/www_bget?mdm:103453644) LOW QUALITY PROTEIN: biotin carboxylase 1, chloroplastic |
| [mdm:103453790](https://www.kegg.jp/dbget-bin/www_bget?mdm:103453790) fructose-bisphosphate aldolase 1, chloroplastic |
| [mdm:103453924](https://www.kegg.jp/dbget-bin/www_bget?mdm:103453924) enolase |
| [mdm:103454483](https://www.kegg.jp/dbget-bin/www_bget?mdm:103454483) S-formylglutathione hydrolase isoform X1 |
| [mdm:103455658](https://www.kegg.jp/dbget-bin/www_bget?mdm:103455658) putative 3,4-dihydroxy-2-butanone kinase |
| [mdm:103455699](https://www.kegg.jp/dbget-bin/www_bget?mdm:103455699) succinate--CoA ligase [ADP-forming] subunit alpha-2, mitochondrial-like |
| [mdm:114822544](https://www.kegg.jp/dbget-bin/www_bget?mdm:114822544) NADP-dependent malic enzyme-like isoform X1 |
| [mdm:114823197](https://www.kegg.jp/dbget-bin/www_bget?mdm:114823197) fructose-bisphosphate aldolase 1, chloroplastic-like |
| [mdm:114824344](https://www.kegg.jp/dbget-bin/www_bget?mdm:114824344) LOW QUALITY PROTEIN: glutamate dehydrogenase 2-like |
| [mdm:114824453](https://www.kegg.jp/dbget-bin/www_bget?mdm:114824453) phosphoglycolate phosphatase 1B, chloroplastic-like |
| [mdm:114825363](https://www.kegg.jp/dbget-bin/www_bget?mdm:114825363) glyceraldehyde-3-phosphate dehydrogenase, cytosolic-like |
| [mdm:114826950](https://www.kegg.jp/dbget-bin/www_bget?mdm:114826950) dihydrolipoyllysine-residue succinyltransferase component of 2-oxoglutarate dehydrogenase complex 1, mitochondrial-like |
| [mdm01210](https://www.kegg.jp/kegg-bin/show_pathway?157443610656263/mdm01210.args) 2-Oxocarboxylic acid metabolism - Malus domestica (apple) ([7](javascript:display('mdm01210'))) |
|  |
| [mdm:103402280](https://www.kegg.jp/dbget-bin/www_bget?mdm:103402280) probable N-acetyl-gamma-glutamyl-phosphate reductase, chloroplastic |
| [mdm:103403075](https://www.kegg.jp/dbget-bin/www_bget?mdm:103403075) glutamate--glyoxylate aminotransferase 2 isoform X1 |
| [mdm:103403601](https://www.kegg.jp/dbget-bin/www_bget?mdm:103403601) 3-isopropylmalate dehydratase large subunit, chloroplastic |
| [mdm:103435112](https://www.kegg.jp/dbget-bin/www_bget?mdm:103435112) acetolactate synthase small subunit 1, chloroplastic-like |
| [mdm:103441178](https://www.kegg.jp/dbget-bin/www_bget?mdm:103441178) aspartate aminotransferase, cytoplasmic |
| [mdm:103442605](https://www.kegg.jp/dbget-bin/www_bget?mdm:103442605) aconitate hydratase, cytoplasmic |
| [mdm:103452041](https://www.kegg.jp/dbget-bin/www_bget?mdm:103452041) glutamate--glyoxylate aminotransferase 2-like |
| [mdm01212](https://www.kegg.jp/kegg-bin/show_pathway?157443610656263/mdm01212.args) Fatty acid metabolism - Malus domestica (apple) ([4](javascript:display('mdm01212')))  [mdm:103411515](https://www.kegg.jp/dbget-bin/www_bget?mdm:103411515) long chain acyl-CoA synthetase 6, peroxisomal-like isoform X1  [mdm:103418786](https://www.kegg.jp/dbget-bin/www_bget?mdm:103418786) uncharacterized protein LOC103418786 |
| [mdm:103429192](https://www.kegg.jp/dbget-bin/www_bget?mdm:103429192) acetyl-CoA acetyltransferase, cytosolic 1 |
| [mdm:103453644](https://www.kegg.jp/dbget-bin/www_bget?mdm:103453644) LOW QUALITY PROTEIN: biotin carboxylase 1, chloroplastic |
| [mdm01230](https://www.kegg.jp/kegg-bin/show_pathway?157443610656263/mdm01230.args) Biosynthesis of amino acids - Malus domestica (apple) ([28](javascript:display('mdm01230'))) |
|  |
| [mdm:103402280](https://www.kegg.jp/dbget-bin/www_bget?mdm:103402280) probable N-acetyl-gamma-glutamyl-phosphate reductase, chloroplastic |
| [mdm:103403075](https://www.kegg.jp/dbget-bin/www_bget?mdm:103403075) glutamate--glyoxylate aminotransferase 2 isoform X1 |
| [mdm:103403601](https://www.kegg.jp/dbget-bin/www_bget?mdm:103403601) 3-isopropylmalate dehydratase large subunit, chloroplastic |
| [mdm:103404212](https://www.kegg.jp/dbget-bin/www_bget?mdm:103404212) ribulose-phosphate 3-epimerase, cytoplasmic isoform-like |
| [mdm:103404478](https://www.kegg.jp/dbget-bin/www_bget?mdm:103404478) chorismate mutase 1, chloroplastic |
| [mdm:103405636](https://www.kegg.jp/dbget-bin/www_bget?mdm:103405636) tyrosine aminotransferase-like |
| [mdm:103416043](https://www.kegg.jp/dbget-bin/www_bget?mdm:103416043) histidinol-phosphate aminotransferase, chloroplastic-like |
| [mdm:103423287](https://www.kegg.jp/dbget-bin/www_bget?mdm:103423287) pyrroline-5-carboxylate reductase-like |
| [mdm:103434138](https://www.kegg.jp/dbget-bin/www_bget?mdm:103434138) cysteine synthase-like isoform X1 |
| [mdm:103435112](https://www.kegg.jp/dbget-bin/www_bget?mdm:103435112) acetolactate synthase small subunit 1, chloroplastic-like |
| [mdm:103436360](https://www.kegg.jp/dbget-bin/www_bget?mdm:103436360) phospho-2-dehydro-3-deoxyheptonate aldolase 1, chloroplastic-like |
| [mdm:103436439](https://www.kegg.jp/dbget-bin/www_bget?mdm:103436439) D-3-phosphoglycerate dehydrogenase 1, chloroplastic-like |
| [mdm:103436467](https://www.kegg.jp/dbget-bin/www_bget?mdm:103436467) LL-diaminopimelate aminotransferase, chloroplastic-like |
| [mdm:103436725](https://www.kegg.jp/dbget-bin/www_bget?mdm:103436725) cysteine synthase-like |
| [mdm:103437705](https://www.kegg.jp/dbget-bin/www_bget?mdm:103437705) anthranilate phosphoribosyltransferase, chloroplastic |
| [mdm:103441178](https://www.kegg.jp/dbget-bin/www_bget?mdm:103441178) aspartate aminotransferase, cytoplasmic |
| [mdm:103442605](https://www.kegg.jp/dbget-bin/www_bget?mdm:103442605) aconitate hydratase, cytoplasmic |
| [mdm:103445180](https://www.kegg.jp/dbget-bin/www_bget?mdm:103445180) LOW QUALITY PROTEIN: LL-diaminopimelate aminotransferase, chloroplastic-like |
| [mdm:103445713](https://www.kegg.jp/dbget-bin/www_bget?mdm:103445713) tryptophan synthase alpha chain-like |
| [mdm:103449400](https://www.kegg.jp/dbget-bin/www_bget?mdm:103449400) fructose-bisphosphate aldolase 6, cytosolic |
| [mdm:103450933](https://www.kegg.jp/dbget-bin/www_bget?mdm:103450933) diaminopimelate epimerase, chloroplastic-like isoform X1 |
| [mdm:103451064](https://www.kegg.jp/dbget-bin/www_bget?mdm:103451064) probable low-specificity L-threonine aldolase 1 |
| [mdm:103452041](https://www.kegg.jp/dbget-bin/www_bget?mdm:103452041) glutamate--glyoxylate aminotransferase 2-like |
| [mdm:103453790](https://www.kegg.jp/dbget-bin/www_bget?mdm:103453790) fructose-bisphosphate aldolase 1, chloroplastic |
| [mdm:103453924](https://www.kegg.jp/dbget-bin/www_bget?mdm:103453924) enolase |
| [mdm:103454765](https://www.kegg.jp/dbget-bin/www_bget?mdm:103454765) shikimate kinase, chloroplastic-like |
| [mdm:114823197](https://www.kegg.jp/dbget-bin/www_bget?mdm:114823197) fructose-bisphosphate aldolase 1, chloroplastic-like |
| [mdm:114825363](https://www.kegg.jp/dbget-bin/www_bget?mdm:114825363) glyceraldehyde-3-phosphate dehydrogenase, cytosolic-like |
| [mdm00010](https://www.kegg.jp/kegg-bin/show_pathway?157443610656263/mdm00010.args) Glycolysis / Gluconeogenesis - Malus domestica (apple) ([14](javascript:display('mdm00010'))) |
|  |
| [mdm:103400922](https://www.kegg.jp/dbget-bin/www_bget?mdm:103400922) MF-60; aldehyde dehydrogenase family 7 member A1 |
| [mdm:103401356](https://www.kegg.jp/dbget-bin/www_bget?mdm:103401356) putative glucose-6-phosphate 1-epimerase |
| [mdm:103401714](https://www.kegg.jp/dbget-bin/www_bget?mdm:103401714) aldehyde dehydrogenase family 3 member H1-like isoform X2 |
| [mdm:103404264](https://www.kegg.jp/dbget-bin/www_bget?mdm:103404264) putative glucose-6-phosphate 1-epimerase isoform X1 |
| [mdm:103408973](https://www.kegg.jp/dbget-bin/www_bget?mdm:103408973) pyruvate dehydrogenase E1 component subunit beta-3, chloroplastic-like |
| [mdm:103419872](https://www.kegg.jp/dbget-bin/www_bget?mdm:103419872) putative glucose-6-phosphate 1-epimerase |
| [mdm:103440430](https://www.kegg.jp/dbget-bin/www_bget?mdm:103440430) glucose-6-phosphate isomerase, cytosolic |
| [mdm:103447094](https://www.kegg.jp/dbget-bin/www_bget?mdm:103447094) NADP-dependent glyceraldehyde-3-phosphate dehydrogenase isoform X2 |
| [mdm:103449400](https://www.kegg.jp/dbget-bin/www_bget?mdm:103449400) fructose-bisphosphate aldolase 6, cytosolic |
| [mdm:103449888](https://www.kegg.jp/dbget-bin/www_bget?mdm:103449888) hexokinase-1-like |
| [mdm:103453790](https://www.kegg.jp/dbget-bin/www_bget?mdm:103453790) fructose-bisphosphate aldolase 1, chloroplastic |
| [mdm:103453924](https://www.kegg.jp/dbget-bin/www_bget?mdm:103453924) enolase |
| [mdm:114823197](https://www.kegg.jp/dbget-bin/www_bget?mdm:114823197) fructose-bisphosphate aldolase 1, chloroplastic-like |
| [mdm:114825363](https://www.kegg.jp/dbget-bin/www_bget?mdm:114825363) glyceraldehyde-3-phosphate dehydrogenase, cytosolic-like |
| [mdm00020](https://www.kegg.jp/kegg-bin/show_pathway?157443610656263/mdm00020.args) Citrate cycle (TCA cycle) - Malus domestica (apple) ([7](javascript:display('mdm00020'))) |
|  |
| [mdm:103403033](https://www.kegg.jp/dbget-bin/www_bget?mdm:103403033) ATP-citrate synthase alpha chain protein 1 |
| [mdm:103408973](https://www.kegg.jp/dbget-bin/www_bget?mdm:103408973) pyruvate dehydrogenase E1 component subunit beta-3, chloroplastic-like |
| [mdm:103424543](https://www.kegg.jp/dbget-bin/www_bget?mdm:103424543) malate dehydrogenase, chloroplastic-like |
| [mdm:103442605](https://www.kegg.jp/dbget-bin/www_bget?mdm:103442605) aconitate hydratase, cytoplasmic |
| [mdm:103452636](https://www.kegg.jp/dbget-bin/www_bget?mdm:103452636) ATP-citrate synthase alpha chain protein 2 isoform X1 |
| [mdm:103455699](https://www.kegg.jp/dbget-bin/www_bget?mdm:103455699) succinate--CoA ligase [ADP-forming] subunit alpha-2, mitochondrial-like |
| [mdm:114826950](https://www.kegg.jp/dbget-bin/www_bget?mdm:114826950) dihydrolipoyllysine-residue succinyltransferase component of 2-oxoglutarate dehydrogenase complex 1, mitochondrial-like |
| [mdm00030](https://www.kegg.jp/kegg-bin/show_pathway?157443610656263/mdm00030.args) Pentose phosphate pathway - Malus domestica (apple) ([8](javascript:display('mdm00030'))) |
|  |
| [mdm:103404212](https://www.kegg.jp/dbget-bin/www_bget?mdm:103404212) ribulose-phosphate 3-epimerase, cytoplasmic isoform-like |
| [mdm:103429189](https://www.kegg.jp/dbget-bin/www_bget?mdm:103429189) glucose-6-phosphate 1-dehydrogenase, chloroplastic-like |
| [mdm:103440430](https://www.kegg.jp/dbget-bin/www_bget?mdm:103440430) glucose-6-phosphate isomerase, cytosolic |
| [mdm:103447094](https://www.kegg.jp/dbget-bin/www_bget?mdm:103447094) NADP-dependent glyceraldehyde-3-phosphate dehydrogenase isoform X2 |
| [mdm:103449400](https://www.kegg.jp/dbget-bin/www_bget?mdm:103449400) fructose-bisphosphate aldolase 6, cytosolic |
| [mdm:103451755](https://www.kegg.jp/dbget-bin/www_bget?mdm:103451755) probable 6-phosphogluconolactonase 1 |
| [mdm:103453790](https://www.kegg.jp/dbget-bin/www_bget?mdm:103453790) fructose-bisphosphate aldolase 1, chloroplastic |
| [mdm:114823197](https://www.kegg.jp/dbget-bin/www_bget?mdm:114823197) fructose-bisphosphate aldolase 1, chloroplastic-like |
| [mdm00040](https://www.kegg.jp/kegg-bin/show_pathway?157443610656263/mdm00040.args) Pentose and glucuronate interconversions - Malus domestica (apple) ([3](javascript:display('mdm00040'))) |
|  |
| [mdm:103404212](https://www.kegg.jp/dbget-bin/www_bget?mdm:103404212) ribulose-phosphate 3-epimerase, cytoplasmic isoform-like |
| [mdm:103415187](https://www.kegg.jp/dbget-bin/www_bget?mdm:103415187) probable pectate lyase 18 |
| [mdm:103428701](https://www.kegg.jp/dbget-bin/www_bget?mdm:103428701) probable polygalacturonase At1g80170 |
| [mdm00051](https://www.kegg.jp/kegg-bin/show_pathway?157443610656263/mdm00051.args) Fructose and mannose metabolism - Malus domestica (apple) ([8](javascript:display('mdm00051'))) |
|  |
| [mdm:103418639](https://www.kegg.jp/dbget-bin/www_bget?mdm:103418639) 6-phosphofructo-2-kinase/fructose-2, 6-bisphosphatase |
| [mdm:103425825](https://www.kegg.jp/dbget-bin/www_bget?mdm:103425825) mannose-1-phosphate guanyltransferase alpha-like |
| [mdm:103441205](https://www.kegg.jp/dbget-bin/www_bget?mdm:103441205) probable fructokinase-6, chloroplastic |
| [mdm:103449400](https://www.kegg.jp/dbget-bin/www_bget?mdm:103449400) fructose-bisphosphate aldolase 6, cytosolic |
| [mdm:103449888](https://www.kegg.jp/dbget-bin/www_bget?mdm:103449888) hexokinase-1-like |
| [mdm:103453790](https://www.kegg.jp/dbget-bin/www_bget?mdm:103453790) fructose-bisphosphate aldolase 1, chloroplastic |
| [mdm:103455658](https://www.kegg.jp/dbget-bin/www_bget?mdm:103455658) putative 3,4-dihydroxy-2-butanone kinase |
| [mdm:114823197](https://www.kegg.jp/dbget-bin/www_bget?mdm:114823197) fructose-bisphosphate aldolase 1, chloroplastic-like |
| [mdm00052](https://www.kegg.jp/kegg-bin/show_pathway?157443610656263/mdm00052.args) Galactose metabolism - Malus domestica (apple) ([1](javascript:display('mdm00052'))) |
|  |
| [mdm:103449888](https://www.kegg.jp/dbget-bin/www_bget?mdm:103449888) hexokinase-1-like |
| [mdm00053](https://www.kegg.jp/kegg-bin/show_pathway?157443610656263/mdm00053.args) Ascorbate and aldarate metabolism - Malus domestica (apple) ([6](javascript:display('mdm00053'))) |
|  |
| [mdm:103400922](https://www.kegg.jp/dbget-bin/www_bget?mdm:103400922) MF-60; aldehyde dehydrogenase family 7 member A1 |
| [mdm:103401714](https://www.kegg.jp/dbget-bin/www_bget?mdm:103401714) aldehyde dehydrogenase family 3 member H1-like isoform X2 |
| [mdm:103410608](https://www.kegg.jp/dbget-bin/www_bget?mdm:103410608) L-ascorbate peroxidase 3-like |
| [mdm:103422491](https://www.kegg.jp/dbget-bin/www_bget?mdm:103422491) inositol-phosphate phosphatase-like |
| [mdm:103433463](https://www.kegg.jp/dbget-bin/www_bget?mdm:103433463) DHAR; glutathione S-transferase DHAR2-like |
| [mdm:103444197](https://www.kegg.jp/dbget-bin/www_bget?mdm:103444197) glutathione S-transferase DHAR3, chloroplastic |
| [mdm00500](https://www.kegg.jp/kegg-bin/show_pathway?157443610656263/mdm00500.args) Starch and sucrose metabolism - Malus domestica (apple) ([9](javascript:display('mdm00500'))) |
|  |
| [mdm:103401914](https://www.kegg.jp/dbget-bin/www_bget?mdm:103401914) beta-amylase 7 isoform X1 |
| [mdm:103405614](https://www.kegg.jp/dbget-bin/www_bget?mdm:103405614) 4-alpha-glucanotransferase, chloroplastic/amyloplastic |
| [mdm:103439436](https://www.kegg.jp/dbget-bin/www_bget?mdm:103439436) granule-bound starch synthase 1, chloroplastic/amyloplastic-like |
| [mdm:103440430](https://www.kegg.jp/dbget-bin/www_bget?mdm:103440430) glucose-6-phosphate isomerase, cytosolic |
| [mdm:103441205](https://www.kegg.jp/dbget-bin/www_bget?mdm:103441205) probable fructokinase-6, chloroplastic |
| [mdm:103446691](https://www.kegg.jp/dbget-bin/www_bget?mdm:103446691) beta-glucosidase 46-like |
| [mdm:103449888](https://www.kegg.jp/dbget-bin/www_bget?mdm:103449888) hexokinase-1-like |
| [mdm:103451863](https://www.kegg.jp/dbget-bin/www_bget?mdm:103451863) AMY10; alpha-amylase 3, chloroplastic-like |
| [mdm:114820473](https://www.kegg.jp/dbget-bin/www_bget?mdm:114820473) isoamylase 1, chloroplastic |
| [mdm00520](https://www.kegg.jp/kegg-bin/show_pathway?157443610656263/mdm00520.args) Amino sugar and nucleotide sugar metabolism - Malus domestica (apple) ([10](javascript:display('mdm00520'))) |
|  |
| [mdm:103400532](https://www.kegg.jp/dbget-bin/www_bget?mdm:103400532) NADH-cytochrome b5 reductase-like protein |
| [mdm:103413054](https://www.kegg.jp/dbget-bin/www_bget?mdm:103413054) cytochrome b5 domain-containing protein RLF |
| [mdm:103425825](https://www.kegg.jp/dbget-bin/www_bget?mdm:103425825) mannose-1-phosphate guanyltransferase alpha-like |
| [mdm:103426493](https://www.kegg.jp/dbget-bin/www_bget?mdm:103426493) acidic endochitinase-like |
| [mdm:103430924](https://www.kegg.jp/dbget-bin/www_bget?mdm:103430924) NADH--cytochrome b5 reductase 1-like |
| [mdm:103434248](https://www.kegg.jp/dbget-bin/www_bget?mdm:103434248) probable UDP-arabinopyranose mutase 5 isoform X1 |
| [mdm:103434574](https://www.kegg.jp/dbget-bin/www_bget?mdm:103434574) beta-hexosaminidase 1-like |
| [mdm:103440430](https://www.kegg.jp/dbget-bin/www_bget?mdm:103440430) glucose-6-phosphate isomerase, cytosolic |
| [mdm:103441205](https://www.kegg.jp/dbget-bin/www_bget?mdm:103441205) probable fructokinase-6, chloroplastic |
| [mdm:103449888](https://www.kegg.jp/dbget-bin/www_bget?mdm:103449888) hexokinase-1-like |
| [mdm00620](https://www.kegg.jp/kegg-bin/show_pathway?157443610656263/mdm00620.args) Pyruvate metabolism - Malus domestica (apple) ([9](javascript:display('mdm00620'))) |
|  |
| [mdm:103400922](https://www.kegg.jp/dbget-bin/www_bget?mdm:103400922) MF-60; aldehyde dehydrogenase family 7 member A1 |
| [mdm:103401714](https://www.kegg.jp/dbget-bin/www_bget?mdm:103401714) aldehyde dehydrogenase family 3 member H1-like isoform X2 |
| [mdm:103408973](https://www.kegg.jp/dbget-bin/www_bget?mdm:103408973) pyruvate dehydrogenase E1 component subunit beta-3, chloroplastic-like |
| [mdm:103424543](https://www.kegg.jp/dbget-bin/www_bget?mdm:103424543) malate dehydrogenase, chloroplastic-like |
| [mdm:103429192](https://www.kegg.jp/dbget-bin/www_bget?mdm:103429192) acetyl-CoA acetyltransferase, cytosolic 1 |
| [mdm:103438582](https://www.kegg.jp/dbget-bin/www_bget?mdm:103438582) hydroxyacylglutathione hydrolase cytoplasmic |
| [mdm:103448063](https://www.kegg.jp/dbget-bin/www_bget?mdm:103448063) lactoylglutathione lyase isoform X1 |
| [mdm:103453644](https://www.kegg.jp/dbget-bin/www_bget?mdm:103453644) LOW QUALITY PROTEIN: biotin carboxylase 1, chloroplastic |
| [mdm:114822544](https://www.kegg.jp/dbget-bin/www_bget?mdm:114822544) NADP-dependent malic enzyme-like isoform X1 |
| [mdm00630](https://www.kegg.jp/kegg-bin/show_pathway?157443610656263/mdm00630.args) Glyoxylate and dicarboxylate metabolism - Malus domestica (apple) ([8](javascript:display('mdm00630'))) |
|  |
| [mdm:103403075](https://www.kegg.jp/dbget-bin/www_bget?mdm:103403075) glutamate--glyoxylate aminotransferase 2 isoform X1 |
| [mdm:103424543](https://www.kegg.jp/dbget-bin/www_bget?mdm:103424543) malate dehydrogenase, chloroplastic-like |
| [mdm:103429192](https://www.kegg.jp/dbget-bin/www_bget?mdm:103429192) acetyl-CoA acetyltransferase, cytosolic 1 |
| [mdm:103437095](https://www.kegg.jp/dbget-bin/www_bget?mdm:103437095) formyltetrahydrofolate deformylase 1, mitochondrial-like isoform X1 |
| [mdm:103442605](https://www.kegg.jp/dbget-bin/www_bget?mdm:103442605) aconitate hydratase, cytoplasmic |
| [mdm:103445750](https://www.kegg.jp/dbget-bin/www_bget?mdm:103445750) peroxisomal (S)-2-hydroxy-acid oxidase-like |
| [mdm:103452041](https://www.kegg.jp/dbget-bin/www_bget?mdm:103452041) glutamate--glyoxylate aminotransferase 2-like |
| [mdm:114824453](https://www.kegg.jp/dbget-bin/www_bget?mdm:114824453) phosphoglycolate phosphatase 1B, chloroplastic-like |
| [mdm00640](https://www.kegg.jp/kegg-bin/show_pathway?157443610656263/mdm00640.args) Propanoate metabolism - Malus domestica (apple) ([5](javascript:display('mdm00640'))) |
|  |
| [mdm:103400579](https://www.kegg.jp/dbget-bin/www_bget?mdm:103400579) 3-hydroxyisobutyryl-CoA hydrolase-like protein 3, mitochondrial |
| [mdm:103401538](https://www.kegg.jp/dbget-bin/www_bget?mdm:103401538) uncharacterized protein LOC103401538 |
| [mdm:103429192](https://www.kegg.jp/dbget-bin/www_bget?mdm:103429192) acetyl-CoA acetyltransferase, cytosolic 1 |
| [mdm:103453644](https://www.kegg.jp/dbget-bin/www_bget?mdm:103453644) LOW QUALITY PROTEIN: biotin carboxylase 1, chloroplastic |
| [mdm:103455699](https://www.kegg.jp/dbget-bin/www_bget?mdm:103455699) succinate--CoA ligase [ADP-forming] subunit alpha-2, mitochondrial-like |
| [mdm00650](https://www.kegg.jp/kegg-bin/show_pathway?157443610656263/mdm00650.args) Butanoate metabolism - Malus domestica (apple) ([3](javascript:display('mdm00650'))) |
|  |
| [mdm:103429192](https://www.kegg.jp/dbget-bin/www_bget?mdm:103429192) acetyl-CoA acetyltransferase, cytosolic 1 |
| [mdm:103435112](https://www.kegg.jp/dbget-bin/www_bget?mdm:103435112) acetolactate synthase small subunit 1, chloroplastic-like |
| [mdm:103454693](https://www.kegg.jp/dbget-bin/www_bget?mdm:103454693) L-2-hydroxyglutarate dehydrogenase, mitochondrial-like isoform X1 |
| [mdm00660](https://www.kegg.jp/kegg-bin/show_pathway?157443610656263/mdm00660.args) C5-Branched dibasic acid metabolism - Malus domestica (apple) ([2](javascript:display('mdm00660'))) |
|  |
| [mdm:103403601](https://www.kegg.jp/dbget-bin/www_bget?mdm:103403601) 3-isopropylmalate dehydratase large subunit, chloroplastic |
| [mdm:103435112](https://www.kegg.jp/dbget-bin/www_bget?mdm:103435112) acetolactate synthase small subunit 1, chloroplastic-like |
| [mdm00562](https://www.kegg.jp/kegg-bin/show_pathway?157443610656263/mdm00562.args) Inositol phosphate metabolism - Malus domestica (apple) ([9](javascript:display('mdm00562'))) |
|  |
| [mdm:103401538](https://www.kegg.jp/dbget-bin/www_bget?mdm:103401538) uncharacterized protein LOC103401538 |
| [mdm:103406415](https://www.kegg.jp/dbget-bin/www_bget?mdm:103406415) phosphoinositide phospholipase C 2-like |
| [mdm:103414619](https://www.kegg.jp/dbget-bin/www_bget?mdm:103414619) phosphoinositide phosphatase SAC8-like |
| [mdm:103422491](https://www.kegg.jp/dbget-bin/www_bget?mdm:103422491) inositol-phosphate phosphatase-like |
| [mdm:103438670](https://www.kegg.jp/dbget-bin/www_bget?mdm:103438670) LOW QUALITY PROTEIN: inositol polyphosphate multikinase beta-like |
| [mdm:103441898](https://www.kegg.jp/dbget-bin/www_bget?mdm:103441898) phosphatidylinositol 4-phosphate 5-kinase 8-like isoform X1 |
| [mdm:103443644](https://www.kegg.jp/dbget-bin/www_bget?mdm:103443644) phosphoinositide phosphatase SAC2-like isoform X1 |
| [mdm:103444921](https://www.kegg.jp/dbget-bin/www_bget?mdm:103444921) inositol-tetrakisphosphate 1-kinase 1-like |
| [mdm:114827525](https://www.kegg.jp/dbget-bin/www_bget?mdm:114827525) putative 1-phosphatidylinositol-3-phosphate 5-kinase FAB1D |
| [mdm00190](https://www.kegg.jp/kegg-bin/show_pathway?157443610656263/mdm00190.args) Oxidative phosphorylation - Malus domestica (apple) ([13](javascript:display('mdm00190'))) |
|  |
| [mdm:103401124](https://www.kegg.jp/dbget-bin/www_bget?mdm:103401124) soluble inorganic pyrophosphatase 6, chloroplastic-like |
| [mdm:103402343](https://www.kegg.jp/dbget-bin/www_bget?mdm:103402343) cytochrome b-c1 complex subunit 6-like |
| [mdm:103414581](https://www.kegg.jp/dbget-bin/www_bget?mdm:103414581) NADH dehydrogenase [ubiquinone] iron-sulfur protein 8-A, mitochondrial |
| [mdm:103416934](https://www.kegg.jp/dbget-bin/www_bget?mdm:103416934) cytochrome b-c1 complex subunit Rieske-4, mitochondrial-like |
| [mdm:103416945](https://www.kegg.jp/dbget-bin/www_bget?mdm:103416945) cytochrome c1-2, heme protein, mitochondrial |
| [mdm:103417231](https://www.kegg.jp/dbget-bin/www_bget?mdm:103417231) V-type proton ATPase subunit D-like |
| [mdm:103420449](https://www.kegg.jp/dbget-bin/www_bget?mdm:103420449) ATP synthase subunit O, mitochondrial-like |
| [mdm:103434495](https://www.kegg.jp/dbget-bin/www_bget?mdm:103434495) ATP synthase gamma chain, chloroplastic-like |
| [mdm:103436074](https://www.kegg.jp/dbget-bin/www_bget?mdm:103436074) NADH dehydrogenase [ubiquinone] flavoprotein 2, mitochondrial-like |
| [mdm:103442089](https://www.kegg.jp/dbget-bin/www_bget?mdm:103442089) V-type proton ATPase subunit C |
| [mdm:103442796](https://www.kegg.jp/dbget-bin/www_bget?mdm:103442796) V-type proton ATPase subunit d2 |
| [mdm:103450546](https://www.kegg.jp/dbget-bin/www_bget?mdm:103450546) V-type proton ATPase 16 kDa proteolipid subunit |
| [mdm:114822925](https://www.kegg.jp/dbget-bin/www_bget?mdm:114822925) NADH dehydrogenase [ubiquinone] 1 beta subcomplex subunit 9-like |
| [mdm00195](https://www.kegg.jp/kegg-bin/show_pathway?157443610656263/mdm00195.args) Photosynthesis - Malus domestica (apple) ([5](javascript:display('mdm00195'))) |
|  |
| [mdm:103405142](https://www.kegg.jp/dbget-bin/www_bget?mdm:103405142) oxygen-evolving enhancer protein 2, chloroplastic-like |
| [mdm:103434495](https://www.kegg.jp/dbget-bin/www_bget?mdm:103434495) ATP synthase gamma chain, chloroplastic-like |
| [mdm:103434631](https://www.kegg.jp/dbget-bin/www_bget?mdm:103434631) oxygen-evolving enhancer protein 3-2, chloroplastic |
| [mdm:103443793](https://www.kegg.jp/dbget-bin/www_bget?mdm:103443793) photosystem I reaction center subunit VI-2, chloroplastic |
| [mdm:103453352](https://www.kegg.jp/dbget-bin/www_bget?mdm:103453352) ferredoxin, root R-B1 |
| [mdm00196](https://www.kegg.jp/kegg-bin/show_pathway?157443610656263/mdm00196.args) Photosynthesis - antenna proteins - Malus domestica (apple) ([1](javascript:display('mdm00196'))) |
|  |
| [mdm:103405001](https://www.kegg.jp/dbget-bin/www_bget?mdm:103405001) chlorophyll a-b binding protein 151, chloroplastic-like |
| [mdm00710](https://www.kegg.jp/kegg-bin/show_pathway?157443610656263/mdm00710.args) Carbon fixation in photosynthetic organisms - Malus domestica (apple) ([10](javascript:display('mdm00710'))) |
|  |
| [mdm:103403075](https://www.kegg.jp/dbget-bin/www_bget?mdm:103403075) glutamate--glyoxylate aminotransferase 2 isoform X1 |
| [mdm:103404212](https://www.kegg.jp/dbget-bin/www_bget?mdm:103404212) ribulose-phosphate 3-epimerase, cytoplasmic isoform-like |
| [mdm:103424543](https://www.kegg.jp/dbget-bin/www_bget?mdm:103424543) malate dehydrogenase, chloroplastic-like |
| [mdm:103441178](https://www.kegg.jp/dbget-bin/www_bget?mdm:103441178) aspartate aminotransferase, cytoplasmic |
| [mdm:103449400](https://www.kegg.jp/dbget-bin/www_bget?mdm:103449400) fructose-bisphosphate aldolase 6, cytosolic |
| [mdm:103452041](https://www.kegg.jp/dbget-bin/www_bget?mdm:103452041) glutamate--glyoxylate aminotransferase 2-like |
| [mdm:103453790](https://www.kegg.jp/dbget-bin/www_bget?mdm:103453790) fructose-bisphosphate aldolase 1, chloroplastic |
| [mdm:114822544](https://www.kegg.jp/dbget-bin/www_bget?mdm:114822544) NADP-dependent malic enzyme-like isoform X1 |
| [mdm:114823197](https://www.kegg.jp/dbget-bin/www_bget?mdm:114823197) fructose-bisphosphate aldolase 1, chloroplastic-like |
| [mdm:114825363](https://www.kegg.jp/dbget-bin/www_bget?mdm:114825363) glyceraldehyde-3-phosphate dehydrogenase, cytosolic-like |
| [mdm00910](https://www.kegg.jp/kegg-bin/show_pathway?157443610656263/mdm00910.args) Nitrogen metabolism - Malus domestica (apple) ([4](javascript:display('mdm00910'))) |
|  |
| [mdm:103403012](https://www.kegg.jp/dbget-bin/www_bget?mdm:103403012) carbonic anhydrase 2-like |
| [mdm:103430865](https://www.kegg.jp/dbget-bin/www_bget?mdm:103430865) cyanate hydratase |
| [mdm:103452065](https://www.kegg.jp/dbget-bin/www_bget?mdm:103452065) glutamate dehydrogenase 1-like |
| [mdm:114824344](https://www.kegg.jp/dbget-bin/www_bget?mdm:114824344) LOW QUALITY PROTEIN: glutamate dehydrogenase 2-like |
| [mdm00920](https://www.kegg.jp/kegg-bin/show_pathway?157443610656263/mdm00920.args) Sulfur metabolism - Malus domestica (apple) ([8](javascript:display('mdm00920'))) |
|  |
| [mdm:103404032](https://www.kegg.jp/dbget-bin/www_bget?mdm:103404032) adenylyl-sulfate kinase 3 isoform X1 |
| [mdm:103408835](https://www.kegg.jp/dbget-bin/www_bget?mdm:103408835) PAP-specific phosphatase HAL2-like |
| [mdm:103409471](https://www.kegg.jp/dbget-bin/www_bget?mdm:103409471) 5'-adenylylsulfate reductase 3, chloroplastic-like |
| [mdm:103410737](https://www.kegg.jp/dbget-bin/www_bget?mdm:103410737) ATP sulfurylase 1, chloroplastic |
| [mdm:103434138](https://www.kegg.jp/dbget-bin/www_bget?mdm:103434138) cysteine synthase-like isoform X1 |
| [mdm:103435398](https://www.kegg.jp/dbget-bin/www_bget?mdm:103435398) thiosulfate/3-mercaptopyruvate sulfurtransferase 1, mitochondrial-like |
| [mdm:103436725](https://www.kegg.jp/dbget-bin/www_bget?mdm:103436725) cysteine synthase-like |
| [mdm:103445864](https://www.kegg.jp/dbget-bin/www_bget?mdm:103445864) thiosulfate/3-mercaptopyruvate sulfurtransferase 1, mitochondrial-like |
|  |
| [mdm00061](https://www.kegg.jp/kegg-bin/show_pathway?157443610656263/mdm00061.args) Fatty acid biosynthesis - Malus domestica (apple) ([3](javascript:display('mdm00061'))) |
|  |
| [mdm:103411515](https://www.kegg.jp/dbget-bin/www_bget?mdm:103411515) long chain acyl-CoA synthetase 6, peroxisomal-like isoform X1 |
| [mdm:103418786](https://www.kegg.jp/dbget-bin/www_bget?mdm:103418786) uncharacterized protein LOC103418786 |
| [mdm:103453644](https://www.kegg.jp/dbget-bin/www_bget?mdm:103453644) LOW QUALITY PROTEIN: biotin carboxylase 1, chloroplastic |
| [mdm00071](https://www.kegg.jp/kegg-bin/show_pathway?157443610656263/mdm00071.args) Fatty acid degradation - Malus domestica (apple) ([4](javascript:display('mdm00071'))) |
|  |
| [mdm:103400922](https://www.kegg.jp/dbget-bin/www_bget?mdm:103400922) MF-60; aldehyde dehydrogenase family 7 member A1 |
| [mdm:103401714](https://www.kegg.jp/dbget-bin/www_bget?mdm:103401714) aldehyde dehydrogenase family 3 member H1-like isoform X2 |
| [mdm:103411515](https://www.kegg.jp/dbget-bin/www_bget?mdm:103411515) long chain acyl-CoA synthetase 6, peroxisomal-like isoform X1 |
| [mdm:103429192](https://www.kegg.jp/dbget-bin/www_bget?mdm:103429192) acetyl-CoA acetyltransferase, cytosolic 1 |
| [mdm00072](https://www.kegg.jp/kegg-bin/show_pathway?157443610656263/mdm00072.args) Synthesis and degradation of ketone bodies - Malus domestica (apple) ([1](javascript:display('mdm00072'))) |
|  |
| [mdm:103429192](https://www.kegg.jp/dbget-bin/www_bget?mdm:103429192) acetyl-CoA acetyltransferase, cytosolic 1 |
| [mdm00100](https://www.kegg.jp/kegg-bin/show_pathway?157443610656263/mdm00100.args) Steroid biosynthesis - Malus domestica (apple) ([4](javascript:display('mdm00100'))) |
|  |
| [mdm:103401301](https://www.kegg.jp/dbget-bin/www_bget?mdm:103401301) triacylglycerol lipase 1 |
| [mdm:103411553](https://www.kegg.jp/dbget-bin/www_bget?mdm:103411553) LOW QUALITY PROTEIN: cycloartenol synthase-like |
| [mdm:103416907](https://www.kegg.jp/dbget-bin/www_bget?mdm:103416907) cycloartenol synthase 2 |
| [mdm:114824777](https://www.kegg.jp/dbget-bin/www_bget?mdm:114824777) squalene synthase-like |
| [mdm00561](https://www.kegg.jp/kegg-bin/show_pathway?157443610656263/mdm00561.args) Glycerolipid metabolism - Malus domestica (apple) ([6](javascript:display('mdm00561'))) |
|  |
| [mdm:103400922](https://www.kegg.jp/dbget-bin/www_bget?mdm:103400922) MF-60; aldehyde dehydrogenase family 7 member A1 |
| [mdm:103401714](https://www.kegg.jp/dbget-bin/www_bget?mdm:103401714) aldehyde dehydrogenase family 3 member H1-like isoform X2 |
| [mdm:103414953](https://www.kegg.jp/dbget-bin/www_bget?mdm:103414953) digalactosyldiacylglycerol synthase 1, chloroplastic-like |
| [mdm:103446060](https://www.kegg.jp/dbget-bin/www_bget?mdm:103446060) digalactosyldiacylglycerol synthase 2, chloroplastic isoform X2 |
| [mdm:103446228](https://www.kegg.jp/dbget-bin/www_bget?mdm:103446228) glycerol-3-phosphate acyltransferase 9 |
| [mdm:103455658](https://www.kegg.jp/dbget-bin/www_bget?mdm:103455658) putative 3,4-dihydroxy-2-butanone kinase |
| [mdm00564](https://www.kegg.jp/kegg-bin/show_pathway?157443610656263/mdm00564.args) Glycerophospholipid metabolism - Malus domestica (apple) ([3](javascript:display('mdm00564'))) |
|  |
| [mdm:103437260](https://www.kegg.jp/dbget-bin/www_bget?mdm:103437260) phospholipase D alpha 1 |
| [mdm:103446228](https://www.kegg.jp/dbget-bin/www_bget?mdm:103446228) glycerol-3-phosphate acyltransferase 9 |
| [mdm:114827740](https://www.kegg.jp/dbget-bin/www_bget?mdm:114827740) lecithin-cholesterol acyltransferase-like 4 |
| [mdm00565](https://www.kegg.jp/kegg-bin/show_pathway?157443610656263/mdm00565.args) Ether lipid metabolism - Malus domestica (apple) ([1](javascript:display('mdm00565'))) |
|  |
| [mdm:103437260](https://www.kegg.jp/dbget-bin/www_bget?mdm:103437260) phospholipase D alpha 1 |
| [mdm00600](https://www.kegg.jp/kegg-bin/show_pathway?157443610656263/mdm00600.args) Sphingolipid metabolism - Malus domestica (apple) ([2](javascript:display('mdm00600'))) |
|  |
| [mdm:103441092](https://www.kegg.jp/dbget-bin/www_bget?mdm:103441092) long chain base biosynthesis protein 1-like isoform X1 |
| [mdm:103453083](https://www.kegg.jp/dbget-bin/www_bget?mdm:103453083) 3-dehydrosphinganine reductase TSC10A |
| [mdm00590](https://www.kegg.jp/kegg-bin/show_pathway?157443610656263/mdm00590.args) Arachidonic acid metabolism - Malus domestica (apple) ([3](javascript:display('mdm00590'))) |
|  |
| [mdm:103420529](https://www.kegg.jp/dbget-bin/www_bget?mdm:103420529) (+)-neomenthol dehydrogenase-like |
| [mdm:103446625](https://www.kegg.jp/dbget-bin/www_bget?mdm:103446625) probable phospholipid hydroperoxide glutathione peroxidase |
| [mdm:103446628](https://www.kegg.jp/dbget-bin/www_bget?mdm:103446628) probable phospholipid hydroperoxide glutathione peroxidase |
| [mdm00592](https://www.kegg.jp/kegg-bin/show_pathway?157443610656263/mdm00592.args) alpha-Linolenic acid metabolism - Malus domestica (apple) ([2](javascript:display('mdm00592'))) |
|  |
| [mdm:103450322](https://www.kegg.jp/dbget-bin/www_bget?mdm:103450322) 4-coumarate--CoA ligase-like 5 |
| [mdm:114827740](https://www.kegg.jp/dbget-bin/www_bget?mdm:114827740) lecithin-cholesterol acyltransferase-like 4 |
| [mdm00230](https://www.kegg.jp/kegg-bin/show_pathway?157443610656263/mdm00230.args) Purine metabolism - Malus domestica (apple) ([8](javascript:display('mdm00230'))) |
|  |
| [mdm:103404032](https://www.kegg.jp/dbget-bin/www_bget?mdm:103404032) adenylyl-sulfate kinase 3 isoform X1 |
| [mdm:103410737](https://www.kegg.jp/dbget-bin/www_bget?mdm:103410737) ATP sulfurylase 1, chloroplastic |
| [mdm:103426071](https://www.kegg.jp/dbget-bin/www_bget?mdm:103426071) probable GTP diphosphokinase RSH2, chloroplastic |
| [mdm:103429705](https://www.kegg.jp/dbget-bin/www_bget?mdm:103429705) adenosine kinase 2 |
| [mdm:103432613](https://www.kegg.jp/dbget-bin/www_bget?mdm:103432613) putative GTP diphosphokinase RSH1, chloroplastic |
| [mdm:103434186](https://www.kegg.jp/dbget-bin/www_bget?mdm:103434186) phosphoribosylaminoimidazole carboxylase, chloroplastic-like isoform X1 |
| [mdm:103449161](https://www.kegg.jp/dbget-bin/www_bget?mdm:103449161) putative GTP diphosphokinase RSH1, chloroplastic |
| [mdm:103452756](https://www.kegg.jp/dbget-bin/www_bget?mdm:103452756) adenylosuccinate synthetase 2, chloroplastic-like |
| [mdm00240](https://www.kegg.jp/kegg-bin/show_pathway?157443610656263/mdm00240.args) Pyrimidine metabolism - Malus domestica (apple) ([5](javascript:display('mdm00240'))) |
|  |
| [mdm:103404202](https://www.kegg.jp/dbget-bin/www_bget?mdm:103404202) uridine kinase-like protein 1, chloroplastic |
| [mdm:103404221](https://www.kegg.jp/dbget-bin/www_bget?mdm:103404221) thymidylate kinase isoform X1 |
| [mdm:103413165](https://www.kegg.jp/dbget-bin/www_bget?mdm:103413165) uncharacterized protein LOC103413165 |
| [mdm:103415799](https://www.kegg.jp/dbget-bin/www_bget?mdm:103415799) dCTP pyrophosphatase 1-like |
| [mdm:103419429](https://www.kegg.jp/dbget-bin/www_bget?mdm:103419429) uridine kinase-like protein 3 |
| [mdm00250](https://www.kegg.jp/kegg-bin/show_pathway?157443610656263/mdm00250.args) Alanine, aspartate and glutamate metabolism - Malus domestica (apple) ([7](javascript:display('mdm00250'))) |
|  |
| [mdm:103403075](https://www.kegg.jp/dbget-bin/www_bget?mdm:103403075) glutamate--glyoxylate aminotransferase 2 isoform X1 |
| [mdm:103420331](https://www.kegg.jp/dbget-bin/www_bget?mdm:103420331) delta-1-pyrroline-5-carboxylate dehydrogenase 12A1, mitochondrial-like |
| [mdm:103441178](https://www.kegg.jp/dbget-bin/www_bget?mdm:103441178) aspartate aminotransferase, cytoplasmic |
| [mdm:103452041](https://www.kegg.jp/dbget-bin/www_bget?mdm:103452041) glutamate--glyoxylate aminotransferase 2-like |
| [mdm:103452065](https://www.kegg.jp/dbget-bin/www_bget?mdm:103452065) glutamate dehydrogenase 1-like |
| [mdm:103452756](https://www.kegg.jp/dbget-bin/www_bget?mdm:103452756) adenylosuccinate synthetase 2, chloroplastic-like |
| [mdm:114824344](https://www.kegg.jp/dbget-bin/www_bget?mdm:114824344) LOW QUALITY PROTEIN: glutamate dehydrogenase 2-like |
| [mdm00260](https://www.kegg.jp/kegg-bin/show_pathway?157443610656263/mdm00260.args) Glycine, serine and threonine metabolism - Malus domestica (apple) ([11](javascript:display('mdm00260'))) |
|  |
| [mdm:103400922](https://www.kegg.jp/dbget-bin/www_bget?mdm:103400922) MF-60; aldehyde dehydrogenase family 7 member A1 |
| [mdm:103403075](https://www.kegg.jp/dbget-bin/www_bget?mdm:103403075) glutamate--glyoxylate aminotransferase 2 isoform X1 |
| [mdm:103407292](https://www.kegg.jp/dbget-bin/www_bget?mdm:103407292) primary amine oxidase-like |
| [mdm:103409732](https://www.kegg.jp/dbget-bin/www_bget?mdm:103409732) betaine aldehyde dehydrogenase 1, chloroplastic |
| [mdm:103429307](https://www.kegg.jp/dbget-bin/www_bget?mdm:103429307) serine racemase isoform X1 |
| [mdm:103436439](https://www.kegg.jp/dbget-bin/www_bget?mdm:103436439) D-3-phosphoglycerate dehydrogenase 1, chloroplastic-like |
| [mdm:103443458](https://www.kegg.jp/dbget-bin/www_bget?mdm:103443458) copper methylamine oxidase-like |
| [mdm:103445713](https://www.kegg.jp/dbget-bin/www_bget?mdm:103445713) tryptophan synthase alpha chain-like |
| [mdm:103451064](https://www.kegg.jp/dbget-bin/www_bget?mdm:103451064) probable low-specificity L-threonine aldolase 1 |
| [mdm:103452038](https://www.kegg.jp/dbget-bin/www_bget?mdm:103452038) copper methylamine oxidase-like |
| [mdm:103452041](https://www.kegg.jp/dbget-bin/www_bget?mdm:103452041) glutamate--glyoxylate aminotransferase 2-like |
| [mdm00270](https://www.kegg.jp/kegg-bin/show_pathway?157443610656263/mdm00270.args) Cysteine and methionine metabolism - Malus domestica (apple) ([12](javascript:display('mdm00270'))) |
|  |
| [mdm:103401300](https://www.kegg.jp/dbget-bin/www_bget?mdm:103401300) methylthioribose kinase-like |
| [mdm:103402794](https://www.kegg.jp/dbget-bin/www_bget?mdm:103402794) S-adenosylmethionine decarboxylase proenzyme-like |
| [mdm:103405636](https://www.kegg.jp/dbget-bin/www_bget?mdm:103405636) tyrosine aminotransferase-like |
| [mdm:103424543](https://www.kegg.jp/dbget-bin/www_bget?mdm:103424543) malate dehydrogenase, chloroplastic-like |
| [mdm:103434138](https://www.kegg.jp/dbget-bin/www_bget?mdm:103434138) cysteine synthase-like isoform X1 |
| [mdm:103435398](https://www.kegg.jp/dbget-bin/www_bget?mdm:103435398) thiosulfate/3-mercaptopyruvate sulfurtransferase 1, mitochondrial-like |
| [mdm:103436439](https://www.kegg.jp/dbget-bin/www_bget?mdm:103436439) D-3-phosphoglycerate dehydrogenase 1, chloroplastic-like |
| [mdm:103436725](https://www.kegg.jp/dbget-bin/www_bget?mdm:103436725) cysteine synthase-like |
| [mdm:103441178](https://www.kegg.jp/dbget-bin/www_bget?mdm:103441178) aspartate aminotransferase, cytoplasmic |
| [mdm:103445864](https://www.kegg.jp/dbget-bin/www_bget?mdm:103445864) thiosulfate/3-mercaptopyruvate sulfurtransferase 1, mitochondrial-like |
| [mdm:103450812](https://www.kegg.jp/dbget-bin/www_bget?mdm:103450812) probable L-cysteine desulfhydrase, chloroplastic |
| [mdm:103452551](https://www.kegg.jp/dbget-bin/www_bget?mdm:103452551) methionine gamma-lyase-like |
| [mdm00280](https://www.kegg.jp/kegg-bin/show_pathway?157443610656263/mdm00280.args) Valine, leucine and isoleucine degradation - Malus domestica (apple) ([7](javascript:display('mdm00280'))) |
|  |
| [mdm:103400579](https://www.kegg.jp/dbget-bin/www_bget?mdm:103400579) 3-hydroxyisobutyryl-CoA hydrolase-like protein 3, mitochondrial |
| [mdm:103400922](https://www.kegg.jp/dbget-bin/www_bget?mdm:103400922) MF-60; aldehyde dehydrogenase family 7 member A1 |
| [mdm:103401538](https://www.kegg.jp/dbget-bin/www_bget?mdm:103401538) uncharacterized protein LOC103401538 |
| [mdm:103401714](https://www.kegg.jp/dbget-bin/www_bget?mdm:103401714) aldehyde dehydrogenase family 3 member H1-like isoform X2 |
| [mdm:103429192](https://www.kegg.jp/dbget-bin/www_bget?mdm:103429192) acetyl-CoA acetyltransferase, cytosolic 1 |
| [mdm:103436482](https://www.kegg.jp/dbget-bin/www_bget?mdm:103436482) methylcrotonoyl-CoA carboxylase beta chain, mitochondrial |
| [mdm:103448970](https://www.kegg.jp/dbget-bin/www_bget?mdm:103448970) methylcrotonoyl-CoA carboxylase subunit alpha, mitochondrial |
| [mdm00290](https://www.kegg.jp/kegg-bin/show_pathway?157443610656263/mdm00290.args) Valine, leucine and isoleucine biosynthesis - Malus domestica (apple) ([2](javascript:display('mdm00290'))) |
|  |
| [mdm:103403601](https://www.kegg.jp/dbget-bin/www_bget?mdm:103403601) 3-isopropylmalate dehydratase large subunit, chloroplastic |
| [mdm:103435112](https://www.kegg.jp/dbget-bin/www_bget?mdm:103435112) acetolactate synthase small subunit 1, chloroplastic-like |
| [mdm00300](https://www.kegg.jp/kegg-bin/show_pathway?157443610656263/mdm00300.args) Lysine biosynthesis - Malus domestica (apple) ([3](javascript:display('mdm00300'))) |
|  |
| [mdm:103436467](https://www.kegg.jp/dbget-bin/www_bget?mdm:103436467) LL-diaminopimelate aminotransferase, chloroplastic-like |
| [mdm:103445180](https://www.kegg.jp/dbget-bin/www_bget?mdm:103445180) LOW QUALITY PROTEIN: LL-diaminopimelate aminotransferase, chloroplastic-like |
| [mdm:103450933](https://www.kegg.jp/dbget-bin/www_bget?mdm:103450933) diaminopimelate epimerase, chloroplastic-like isoform X1 |
| [mdm00310](https://www.kegg.jp/kegg-bin/show_pathway?157443610656263/mdm00310.args) Lysine degradation - Malus domestica (apple) ([7](javascript:display('mdm00310'))) |
|  |
| [mdm:103400922](https://www.kegg.jp/dbget-bin/www_bget?mdm:103400922) MF-60; aldehyde dehydrogenase family 7 member A1 |
| [mdm:103401714](https://www.kegg.jp/dbget-bin/www_bget?mdm:103401714) aldehyde dehydrogenase family 3 member H1-like isoform X2 |
| [mdm:103429192](https://www.kegg.jp/dbget-bin/www_bget?mdm:103429192) acetyl-CoA acetyltransferase, cytosolic 1 |
| [mdm:103438644](https://www.kegg.jp/dbget-bin/www_bget?mdm:103438644) histone-lysine N-methyltransferase, H3 lysine-9 specific SUVH1-like |
| [mdm:103442704](https://www.kegg.jp/dbget-bin/www_bget?mdm:103442704) histone-lysine N-methyltransferase, H3 lysine-9 specific SUVH4 |
| [mdm:103456183](https://www.kegg.jp/dbget-bin/www_bget?mdm:103456183) calmodulin-lysine N-methyltransferase isoform X2 |
| [mdm:114826950](https://www.kegg.jp/dbget-bin/www_bget?mdm:114826950) dihydrolipoyllysine-residue succinyltransferase component of 2-oxoglutarate dehydrogenase complex 1, mitochondrial-like |
| [mdm00220](https://www.kegg.jp/kegg-bin/show_pathway?157443610656263/mdm00220.args) Arginine biosynthesis - Malus domestica (apple) ([6](javascript:display('mdm00220'))) |
|  |
| [mdm:103402280](https://www.kegg.jp/dbget-bin/www_bget?mdm:103402280) probable N-acetyl-gamma-glutamyl-phosphate reductase, chloroplastic |
| [mdm:103403075](https://www.kegg.jp/dbget-bin/www_bget?mdm:103403075) glutamate--glyoxylate aminotransferase 2 isoform X1 |
| [mdm:103441178](https://www.kegg.jp/dbget-bin/www_bget?mdm:103441178) aspartate aminotransferase, cytoplasmic |
| [mdm:103452041](https://www.kegg.jp/dbget-bin/www_bget?mdm:103452041) glutamate--glyoxylate aminotransferase 2-like |
| [mdm:103452065](https://www.kegg.jp/dbget-bin/www_bget?mdm:103452065) glutamate dehydrogenase 1-like |
| [mdm:114824344](https://www.kegg.jp/dbget-bin/www_bget?mdm:114824344) LOW QUALITY PROTEIN: glutamate dehydrogenase 2-like |
| [mdm00330](https://www.kegg.jp/kegg-bin/show_pathway?157443610656263/mdm00330.args) Arginine and proline metabolism - Malus domestica (apple) ([10](javascript:display('mdm00330'))) |
|  |
| [mdm:103400922](https://www.kegg.jp/dbget-bin/www_bget?mdm:103400922) MF-60; aldehyde dehydrogenase family 7 member A1 |
| [mdm:103401040](https://www.kegg.jp/dbget-bin/www_bget?mdm:103401040) probable prolyl 4-hydroxylase 10 |
| [mdm:103401714](https://www.kegg.jp/dbget-bin/www_bget?mdm:103401714) aldehyde dehydrogenase family 3 member H1-like isoform X2 |
| [mdm:103402794](https://www.kegg.jp/dbget-bin/www_bget?mdm:103402794) S-adenosylmethionine decarboxylase proenzyme-like |
| [mdm:103420331](https://www.kegg.jp/dbget-bin/www_bget?mdm:103420331) delta-1-pyrroline-5-carboxylate dehydrogenase 12A1, mitochondrial-like |
| [mdm:103423287](https://www.kegg.jp/dbget-bin/www_bget?mdm:103423287) pyrroline-5-carboxylate reductase-like |
| [mdm:103438425](https://www.kegg.jp/dbget-bin/www_bget?mdm:103438425) polyamine oxidase 1 |
| [mdm:103439769](https://www.kegg.jp/dbget-bin/www_bget?mdm:103439769) probable prolyl 4-hydroxylase 12 isoform X1 |
| [mdm:103441178](https://www.kegg.jp/dbget-bin/www_bget?mdm:103441178) aspartate aminotransferase, cytoplasmic |
| [mdm:103455898](https://www.kegg.jp/dbget-bin/www_bget?mdm:103455898) probable prolyl 4-hydroxylase 10 |
| [mdm00340](https://www.kegg.jp/kegg-bin/show_pathway?157443610656263/mdm00340.args) Histidine metabolism - Malus domestica (apple) ([3](javascript:display('mdm00340'))) |
|  |
| [mdm:103400922](https://www.kegg.jp/dbget-bin/www_bget?mdm:103400922) MF-60; aldehyde dehydrogenase family 7 member A1 |
| [mdm:103401714](https://www.kegg.jp/dbget-bin/www_bget?mdm:103401714) aldehyde dehydrogenase family 3 member H1-like isoform X2 |
| [mdm:103416043](https://www.kegg.jp/dbget-bin/www_bget?mdm:103416043) histidinol-phosphate aminotransferase, chloroplastic-like |
| [mdm00350](https://www.kegg.jp/kegg-bin/show_pathway?157443610656263/mdm00350.args) Tyrosine metabolism - Malus domestica (apple) ([6](javascript:display('mdm00350'))) |
|  |
| [mdm:103405636](https://www.kegg.jp/dbget-bin/www_bget?mdm:103405636) tyrosine aminotransferase-like |
| [mdm:103407292](https://www.kegg.jp/dbget-bin/www_bget?mdm:103407292) primary amine oxidase-like |
| [mdm:103416043](https://www.kegg.jp/dbget-bin/www_bget?mdm:103416043) histidinol-phosphate aminotransferase, chloroplastic-like |
| [mdm:103441178](https://www.kegg.jp/dbget-bin/www_bget?mdm:103441178) aspartate aminotransferase, cytoplasmic |
| [mdm:103443458](https://www.kegg.jp/dbget-bin/www_bget?mdm:103443458) copper methylamine oxidase-like |
| [mdm:103452038](https://www.kegg.jp/dbget-bin/www_bget?mdm:103452038) copper methylamine oxidase-like |
| [mdm00360](https://www.kegg.jp/kegg-bin/show_pathway?157443610656263/mdm00360.args) Phenylalanine metabolism - Malus domestica (apple) ([6](javascript:display('mdm00360'))) |
|  |
| [mdm:103405636](https://www.kegg.jp/dbget-bin/www_bget?mdm:103405636) tyrosine aminotransferase-like |
| [mdm:103407292](https://www.kegg.jp/dbget-bin/www_bget?mdm:103407292) primary amine oxidase-like |
| [mdm:103416043](https://www.kegg.jp/dbget-bin/www_bget?mdm:103416043) histidinol-phosphate aminotransferase, chloroplastic-like |
| [mdm:103441178](https://www.kegg.jp/dbget-bin/www_bget?mdm:103441178) aspartate aminotransferase, cytoplasmic |
| [mdm:103443458](https://www.kegg.jp/dbget-bin/www_bget?mdm:103443458) copper methylamine oxidase-like |
| [mdm:103452038](https://www.kegg.jp/dbget-bin/www_bget?mdm:103452038) copper methylamine oxidase-like |
| [mdm00380](https://www.kegg.jp/kegg-bin/show_pathway?157443610656263/mdm00380.args) Tryptophan metabolism - Malus domestica (apple) ([4](javascript:display('mdm00380'))) |
|  |
| [mdm:103400922](https://www.kegg.jp/dbget-bin/www_bget?mdm:103400922) MF-60; aldehyde dehydrogenase family 7 member A1 |
| [mdm:103401714](https://www.kegg.jp/dbget-bin/www_bget?mdm:103401714) aldehyde dehydrogenase family 3 member H1-like isoform X2 |
| [mdm:103429192](https://www.kegg.jp/dbget-bin/www_bget?mdm:103429192) acetyl-CoA acetyltransferase, cytosolic 1 |
| [mdm:114826950](https://www.kegg.jp/dbget-bin/www_bget?mdm:114826950) dihydrolipoyllysine-residue succinyltransferase component of 2-oxoglutarate dehydrogenase complex 1, mitochondrial-like |
| [mdm00400](https://www.kegg.jp/kegg-bin/show_pathway?157443610656263/mdm00400.args) Phenylalanine, tyrosine and tryptophan biosynthesis - Malus domestica (apple) ([8](javascript:display('mdm00400'))) |
|  |
| [mdm:103404478](https://www.kegg.jp/dbget-bin/www_bget?mdm:103404478) chorismate mutase 1, chloroplastic |
| [mdm:103405636](https://www.kegg.jp/dbget-bin/www_bget?mdm:103405636) tyrosine aminotransferase-like |
| [mdm:103416043](https://www.kegg.jp/dbget-bin/www_bget?mdm:103416043) histidinol-phosphate aminotransferase, chloroplastic-like |
| [mdm:103436360](https://www.kegg.jp/dbget-bin/www_bget?mdm:103436360) phospho-2-dehydro-3-deoxyheptonate aldolase 1, chloroplastic-like |
| [mdm:103437705](https://www.kegg.jp/dbget-bin/www_bget?mdm:103437705) anthranilate phosphoribosyltransferase, chloroplastic |
| [mdm:103441178](https://www.kegg.jp/dbget-bin/www_bget?mdm:103441178) aspartate aminotransferase, cytoplasmic |
| [mdm:103445713](https://www.kegg.jp/dbget-bin/www_bget?mdm:103445713) tryptophan synthase alpha chain-like |
| [mdm:103454765](https://www.kegg.jp/dbget-bin/www_bget?mdm:103454765) shikimate kinase, chloroplastic-like |
| [mdm00410](https://www.kegg.jp/kegg-bin/show_pathway?157443610656263/mdm00410.args) beta-Alanine metabolism - Malus domestica (apple) ([8](javascript:display('mdm00410'))) |
|  |
| [mdm:103400579](https://www.kegg.jp/dbget-bin/www_bget?mdm:103400579) 3-hydroxyisobutyryl-CoA hydrolase-like protein 3, mitochondrial |
| [mdm:103400922](https://www.kegg.jp/dbget-bin/www_bget?mdm:103400922) MF-60; aldehyde dehydrogenase family 7 member A1 |
| [mdm:103401538](https://www.kegg.jp/dbget-bin/www_bget?mdm:103401538) uncharacterized protein LOC103401538 |
| [mdm:103401714](https://www.kegg.jp/dbget-bin/www_bget?mdm:103401714) aldehyde dehydrogenase family 3 member H1-like isoform X2 |
| [mdm:103407292](https://www.kegg.jp/dbget-bin/www_bget?mdm:103407292) primary amine oxidase-like |
| [mdm:103438425](https://www.kegg.jp/dbget-bin/www_bget?mdm:103438425) polyamine oxidase 1 |
| [mdm:103443458](https://www.kegg.jp/dbget-bin/www_bget?mdm:103443458) copper methylamine oxidase-like |
| [mdm:103452038](https://www.kegg.jp/dbget-bin/www_bget?mdm:103452038) copper methylamine oxidase-like |
| [mdm00450](https://www.kegg.jp/kegg-bin/show_pathway?157443610656263/mdm00450.args) Selenocompound metabolism - Malus domestica (apple) ([2](javascript:display('mdm00450'))) |
|  |
| [mdm:103410737](https://www.kegg.jp/dbget-bin/www_bget?mdm:103410737) ATP sulfurylase 1, chloroplastic |
| [mdm:103452551](https://www.kegg.jp/dbget-bin/www_bget?mdm:103452551) methionine gamma-lyase-like |
| [mdm00460](https://www.kegg.jp/kegg-bin/show_pathway?157443610656263/mdm00460.args) Cyanoamino acid metabolism - Malus domestica (apple) ([1](javascript:display('mdm00460'))) |
|  |
| [mdm:103446691](https://www.kegg.jp/dbget-bin/www_bget?mdm:103446691) beta-glucosidase 46-like |
| [mdm00480](https://www.kegg.jp/kegg-bin/show_pathway?157443610656263/mdm00480.args) Glutathione metabolism - Malus domestica (apple) ([10](javascript:display('mdm00480'))) |
|  |
| [mdm:103410608](https://www.kegg.jp/dbget-bin/www_bget?mdm:103410608) L-ascorbate peroxidase 3-like |
| [mdm:103423404](https://www.kegg.jp/dbget-bin/www_bget?mdm:103423404) glutathione S-transferase L3-like |
| [mdm:103429189](https://www.kegg.jp/dbget-bin/www_bget?mdm:103429189) glucose-6-phosphate 1-dehydrogenase, chloroplastic-like |
| [mdm:103433463](https://www.kegg.jp/dbget-bin/www_bget?mdm:103433463) DHAR; glutathione S-transferase DHAR2-like |
| [mdm:103441211](https://www.kegg.jp/dbget-bin/www_bget?mdm:103441211) gamma-glutamylcyclotransferase 2-1-like |
| [mdm:103444197](https://www.kegg.jp/dbget-bin/www_bget?mdm:103444197) glutathione S-transferase DHAR3, chloroplastic |
| [mdm:103446625](https://www.kegg.jp/dbget-bin/www_bget?mdm:103446625) probable phospholipid hydroperoxide glutathione peroxidase |
| [mdm:103446628](https://www.kegg.jp/dbget-bin/www_bget?mdm:103446628) probable phospholipid hydroperoxide glutathione peroxidase |
| [mdm:103451886](https://www.kegg.jp/dbget-bin/www_bget?mdm:103451886) glutathione S-transferase U17-like |
| [mdm:114825652](https://www.kegg.jp/dbget-bin/www_bget?mdm:114825652) puromycin-sensitive aminopeptidase-like isoform X1 |
| [mdm00510](https://www.kegg.jp/kegg-bin/show_pathway?157443610656263/mdm00510.args) N-Glycan biosynthesis - Malus domestica (apple) ([2](javascript:display('mdm00510'))) |
|  |
| [mdm:103431468](https://www.kegg.jp/dbget-bin/www_bget?mdm:103431468) beta-1,4-mannosyl-glycoprotein 4-beta-N-acetylglucosaminyltransferase |
| [mdm:103440661](https://www.kegg.jp/dbget-bin/www_bget?mdm:103440661) LOW QUALITY PROTEIN: dolichyl-diphosphooligosaccharide--protein glycosyltransferase subunit 1B-like |
| [mdm00513](https://www.kegg.jp/kegg-bin/show_pathway?157443610656263/mdm00513.args) Various types of N-glycan biosynthesis - Malus domestica (apple) ([2](javascript:display('mdm00513'))) |
|  |
| [mdm:103434574](https://www.kegg.jp/dbget-bin/www_bget?mdm:103434574) beta-hexosaminidase 1-like |
| [mdm:103440661](https://www.kegg.jp/dbget-bin/www_bget?mdm:103440661) LOW QUALITY PROTEIN: dolichyl-diphosphooligosaccharide--protein glycosyltransferase subunit 1B-like |
| [mdm00531](https://www.kegg.jp/kegg-bin/show_pathway?157443610656263/mdm00531.args) Glycosaminoglycan degradation - Malus domestica (apple) ([1](javascript:display('mdm00531'))) |
|  |
| [mdm:103434574](https://www.kegg.jp/dbget-bin/www_bget?mdm:103434574) beta-hexosaminidase 1-like |
| [mdm00603](https://www.kegg.jp/kegg-bin/show_pathway?157443610656263/mdm00603.args) Glycosphingolipid biosynthesis - globo and isoglobo series - Malus domestica (apple) ([1](javascript:display('mdm00603'))) |
|  |
| [mdm:103434574](https://www.kegg.jp/dbget-bin/www_bget?mdm:103434574) beta-hexosaminidase 1-like |
| [mdm00604](https://www.kegg.jp/kegg-bin/show_pathway?157443610656263/mdm00604.args) Glycosphingolipid biosynthesis - ganglio series - Malus domestica (apple) ([1](javascript:display('mdm00604'))) |
|  |
| [mdm:103434574](https://www.kegg.jp/dbget-bin/www_bget?mdm:103434574) beta-hexosaminidase 1-like |
| [mdm00511](https://www.kegg.jp/kegg-bin/show_pathway?157443610656263/mdm00511.args) Other glycan degradation - Malus domestica (apple) ([1](javascript:display('mdm00511'))) |
|  |
| [mdm:103434574](https://www.kegg.jp/dbget-bin/www_bget?mdm:103434574) beta-hexosaminidase 1-like |
| [mdm00730](https://www.kegg.jp/kegg-bin/show_pathway?157443610656263/mdm00730.args) Thiamine metabolism - Malus domestica (apple) ([2](javascript:display('mdm00730'))) |
|  |
| [mdm:103432577](https://www.kegg.jp/dbget-bin/www_bget?mdm:103432577) thiamine pyrophosphokinase 1-like isoform X1 |
| [mdm:103447435](https://www.kegg.jp/dbget-bin/www_bget?mdm:103447435) LOW QUALITY PROTEIN: phosphomethylpyrimidine synthase, chloroplastic |
| [mdm00740](https://www.kegg.jp/kegg-bin/show_pathway?157443610656263/mdm00740.args) Riboflavin metabolism - Malus domestica (apple) ([1](javascript:display('mdm00740'))) |
|  |
| [mdm:103403391](https://www.kegg.jp/dbget-bin/www_bget?mdm:103403391) FAD synthetase 2, chloroplastic-like isoform X2 |
| [mdm00770](https://www.kegg.jp/kegg-bin/show_pathway?157443610656263/mdm00770.args) Pantothenate and CoA biosynthesis - Malus domestica (apple) ([2](javascript:display('mdm00770'))) |
|  |
| [mdm:103435112](https://www.kegg.jp/dbget-bin/www_bget?mdm:103435112) acetolactate synthase small subunit 1, chloroplastic-like |
| [mdm:103455216](https://www.kegg.jp/dbget-bin/www_bget?mdm:103455216) pantothenate kinase 2 |
| [mdm00790](https://www.kegg.jp/kegg-bin/show_pathway?157443610656263/mdm00790.args) Folate biosynthesis - Malus domestica (apple) ([2](javascript:display('mdm00790'))) |
|  |
| [mdm:103408080](https://www.kegg.jp/dbget-bin/www_bget?mdm:103408080) gamma-glutamyl hydrolase 2-like |
| [mdm:103420529](https://www.kegg.jp/dbget-bin/www_bget?mdm:103420529) (+)-neomenthol dehydrogenase-like |
| [mdm00670](https://www.kegg.jp/kegg-bin/show_pathway?157443610656263/mdm00670.args) One carbon pool by folate - Malus domestica (apple) ([2](javascript:display('mdm00670'))) |
|  |
| [mdm:103437095](https://www.kegg.jp/dbget-bin/www_bget?mdm:103437095) formyltetrahydrofolate deformylase 1, mitochondrial-like isoform X1 |
| [mdm:103437782](https://www.kegg.jp/dbget-bin/www_bget?mdm:103437782) 5-formyltetrahydrofolate cyclo-ligase, mitochondrial-like |
| [mdm00860](https://www.kegg.jp/kegg-bin/show_pathway?157443610656263/mdm00860.args) Porphyrin and chlorophyll metabolism - Malus domestica (apple) ([4](javascript:display('mdm00860'))) |
|  |
| [mdm:103401375](https://www.kegg.jp/dbget-bin/www_bget?mdm:103401375) red chlorophyll catabolite reductase, chloroplastic |
| [mdm:103404567](https://www.kegg.jp/dbget-bin/www_bget?mdm:103404567) sirohydrochlorin ferrochelatase, chloroplastic |
| [mdm:103446341](https://www.kegg.jp/dbget-bin/www_bget?mdm:103446341) magnesium-chelatase subunit ChlI, chloroplastic |
| [mdm:103456287](https://www.kegg.jp/dbget-bin/www_bget?mdm:103456287) magnesium-chelatase subunit ChlH, chloroplastic |
| [mdm00130](https://www.kegg.jp/kegg-bin/show_pathway?157443610656263/mdm00130.args) Ubiquinone and other terpenoid-quinone biosynthesis - Malus domestica (apple) ([3](javascript:display('mdm00130'))) |
|  |
| [mdm:103405636](https://www.kegg.jp/dbget-bin/www_bget?mdm:103405636) tyrosine aminotransferase-like |
| [mdm:103425450](https://www.kegg.jp/dbget-bin/www_bget?mdm:103425450) protein PHYLLO, chloroplastic isoform X1 |
| [mdm:103444873](https://www.kegg.jp/dbget-bin/www_bget?mdm:103444873) uncharacterized protein LOC103444873 isoform X1 |
| [mdm00900](https://www.kegg.jp/kegg-bin/show_pathway?157443610656263/mdm00900.args) Terpenoid backbone biosynthesis - Malus domestica (apple) ([3](javascript:display('mdm00900'))) |
|  |
| [mdm:103403394](https://www.kegg.jp/dbget-bin/www_bget?mdm:103403394) protein-S-isoprenylcysteine O-methyltransferase A-like isoform X1 |
| [mdm:103420733](https://www.kegg.jp/dbget-bin/www_bget?mdm:103420733) 4-hydroxy-3-methylbut-2-enyl diphosphate reductase, chloroplastic |
| [mdm:103429192](https://www.kegg.jp/dbget-bin/www_bget?mdm:103429192) acetyl-CoA acetyltransferase, cytosolic 1 |
| [mdm00902](https://www.kegg.jp/kegg-bin/show_pathway?157443610656263/mdm00902.args) Monoterpenoid biosynthesis - Malus domestica (apple) ([1](javascript:display('mdm00902'))) |
|  |
| [mdm:103437348](https://www.kegg.jp/dbget-bin/www_bget?mdm:103437348) salutaridine reductase-like isoform X2 |
| [mdm00909](https://www.kegg.jp/kegg-bin/show_pathway?157443610656263/mdm00909.args) Sesquiterpenoid and triterpenoid biosynthesis - Malus domestica (apple) ([1](javascript:display('mdm00909'))) |
|  |
| [mdm:114824777](https://www.kegg.jp/dbget-bin/www_bget?mdm:114824777) squalene synthase-like |
| [mdm00904](https://www.kegg.jp/kegg-bin/show_pathway?157443610656263/mdm00904.args) Diterpenoid biosynthesis - Malus domestica (apple) ([1](javascript:display('mdm00904'))) |
|  |
| [mdm:103441571](https://www.kegg.jp/dbget-bin/www_bget?mdm:103441571) ent-kaurene oxidase, chloroplastic-like |
| [mdm00906](https://www.kegg.jp/kegg-bin/show_pathway?157443610656263/mdm00906.args) Carotenoid biosynthesis - Malus domestica (apple) ([1](javascript:display('mdm00906'))) |
|  |
| [mdm:114819149](https://www.kegg.jp/dbget-bin/www_bget?mdm:114819149) xanthoxin dehydrogenase-like |
| [mdm00903](https://www.kegg.jp/kegg-bin/show_pathway?157443610656263/mdm00903.args) Limonene and pinene degradation - Malus domestica (apple) ([1](javascript:display('mdm00903'))) |
|  |
| [mdm:103401714](https://www.kegg.jp/dbget-bin/www_bget?mdm:103401714) aldehyde dehydrogenase family 3 member H1-like isoform X2 |
| [mdm00940](https://www.kegg.jp/kegg-bin/show_pathway?157443610656263/mdm00940.args) Phenylpropanoid biosynthesis - Malus domestica (apple) ([9](javascript:display('mdm00940'))) |
|  |
| [mdm:103403337](https://www.kegg.jp/dbget-bin/www_bget?mdm:103403337) vinorine synthase |
| [mdm:103420099](https://www.kegg.jp/dbget-bin/www_bget?mdm:103420099) cinnamyl alcohol dehydrogenase 1 |
| [mdm:103422058](https://www.kegg.jp/dbget-bin/www_bget?mdm:103422058) peroxidase 12-like |
| [mdm:103422468](https://www.kegg.jp/dbget-bin/www_bget?mdm:103422468) peroxidase 42-like |
| [mdm:103433738](https://www.kegg.jp/dbget-bin/www_bget?mdm:103433738) cationic peroxidase 1-like |
| [mdm:103438384](https://www.kegg.jp/dbget-bin/www_bget?mdm:103438384) UDP-glucose flavonoid 3-O-glucosyltransferase 7-like |
| [mdm:103446691](https://www.kegg.jp/dbget-bin/www_bget?mdm:103446691) beta-glucosidase 46-like |
| [mdm:103448267](https://www.kegg.jp/dbget-bin/www_bget?mdm:103448267) caffeoyl-CoA O-methyltransferase |
| [mdm:114826994](https://www.kegg.jp/dbget-bin/www_bget?mdm:114826994) peroxidase 17-like |
| [mdm00945](https://www.kegg.jp/kegg-bin/show_pathway?157443610656263/mdm00945.args) Stilbenoid, diarylheptanoid and gingerol biosynthesis - Malus domestica (apple) ([2](javascript:display('mdm00945'))) |
|  |
| [mdm:103403337](https://www.kegg.jp/dbget-bin/www_bget?mdm:103403337) vinorine synthase |
| [mdm:103448267](https://www.kegg.jp/dbget-bin/www_bget?mdm:103448267) caffeoyl-CoA O-methyltransferase |
| [mdm00941](https://www.kegg.jp/kegg-bin/show_pathway?157443610656263/mdm00941.args) Flavonoid biosynthesis - Malus domestica (apple) ([5](javascript:display('mdm00941'))) |
|  |
| [mdm:103403337](https://www.kegg.jp/dbget-bin/www_bget?mdm:103403337) vinorine synthase |
| [mdm:103434638](https://www.kegg.jp/dbget-bin/www_bget?mdm:103434638) anthocyanidin reductase |
| [mdm:103443512](https://www.kegg.jp/dbget-bin/www_bget?mdm:103443512) polyketide synthase 5-like |
| [mdm:103448267](https://www.kegg.jp/dbget-bin/www_bget?mdm:103448267) caffeoyl-CoA O-methyltransferase |
| [mdm:103455413](https://www.kegg.jp/dbget-bin/www_bget?mdm:103455413) flavonoid 3'-monooxygenase |
| [mdm00944](https://www.kegg.jp/kegg-bin/show_pathway?157443610656263/mdm00944.args) Flavone and flavonol biosynthesis - Malus domestica (apple) ([1](javascript:display('mdm00944'))) |
|  |
| [mdm:103455413](https://www.kegg.jp/dbget-bin/www_bget?mdm:103455413) flavonoid 3'-monooxygenase |
| [mdm00950](https://www.kegg.jp/kegg-bin/show_pathway?157443610656263/mdm00950.args) Isoquinoline alkaloid biosynthesis - Malus domestica (apple) ([5](javascript:display('mdm00950'))) |
|  |
| [mdm:103405636](https://www.kegg.jp/dbget-bin/www_bget?mdm:103405636) tyrosine aminotransferase-like |
| [mdm:103407292](https://www.kegg.jp/dbget-bin/www_bget?mdm:103407292) primary amine oxidase-like |
| [mdm:103441178](https://www.kegg.jp/dbget-bin/www_bget?mdm:103441178) aspartate aminotransferase, cytoplasmic |
| [mdm:103443458](https://www.kegg.jp/dbget-bin/www_bget?mdm:103443458) copper methylamine oxidase-like |
| [mdm:103452038](https://www.kegg.jp/dbget-bin/www_bget?mdm:103452038) copper methylamine oxidase-like |
|  |
| [mdm00960](https://www.kegg.jp/kegg-bin/show_pathway?157443610656263/mdm00960.args) Tropane, piperidine and pyridine alkaloid biosynthesis - Malus domestica (apple) ([7](javascript:display('mdm00960'))) |
|  |
| [mdm:103405636](https://www.kegg.jp/dbget-bin/www_bget?mdm:103405636) tyrosine aminotransferase-like |
| [mdm:103407292](https://www.kegg.jp/dbget-bin/www_bget?mdm:103407292) primary amine oxidase-like |
| [mdm:103416043](https://www.kegg.jp/dbget-bin/www_bget?mdm:103416043) histidinol-phosphate aminotransferase, chloroplastic-like |
| [mdm:103422717](https://www.kegg.jp/dbget-bin/www_bget?mdm:103422717) LOW QUALITY PROTEIN: tropinone reductase homolog At2g29330-like |
| [mdm:103441178](https://www.kegg.jp/dbget-bin/www_bget?mdm:103441178) aspartate aminotransferase, cytoplasmic |
| [mdm:103443458](https://www.kegg.jp/dbget-bin/www_bget?mdm:103443458) copper methylamine oxidase-like |
| [mdm:103452038](https://www.kegg.jp/dbget-bin/www_bget?mdm:103452038) copper methylamine oxidase-like |
| [mdm00966](https://www.kegg.jp/kegg-bin/show_pathway?157443610656263/mdm00966.args) Glucosinolate biosynthesis - Malus domestica (apple) ([1](javascript:display('mdm00966'))) |
|  |
| [mdm:103403601](https://www.kegg.jp/dbget-bin/www_bget?mdm:103403601) 3-isopropylmalate dehydratase large subunit, chloroplastic |
| [mdm00261](https://www.kegg.jp/kegg-bin/show_pathway?157443610656263/mdm00261.args) Monobactam biosynthesis - Malus domestica (apple) ([1](javascript:display('mdm00261'))) |
|  |
| [mdm:103410737](https://www.kegg.jp/dbget-bin/www_bget?mdm:103410737) ATP sulfurylase 1, chloroplastic |
| [mdm03020](https://www.kegg.jp/kegg-bin/show_pathway?157443610656263/mdm03020.args) RNA polymerase - Malus domestica (apple) ([3](javascript:display('mdm03020'))) |
|  |
| [mdm:103431694](https://www.kegg.jp/dbget-bin/www_bget?mdm:103431694) DNA-directed RNA polymerases II and IV subunit 5A-like isoform X1 |
| [mdm:103446412](https://www.kegg.jp/dbget-bin/www_bget?mdm:103446412) LOW QUALITY PROTEIN: DNA-directed RNA polymerase II subunit RPB2 |
| [mdm:114825051](https://www.kegg.jp/dbget-bin/www_bget?mdm:114825051) DNA-directed RNA polymerases IV and V subunit 4-like isoform X1 |
| [mdm03022](https://www.kegg.jp/kegg-bin/show_pathway?157443610656263/mdm03022.args) Basal transcription factors - Malus domestica (apple) ([3](javascript:display('mdm03022'))) |
|  |
| [mdm:103410922](https://www.kegg.jp/dbget-bin/www_bget?mdm:103410922) transcription initiation factor IIA subunit 2 |
| [mdm:103411408](https://www.kegg.jp/dbget-bin/www_bget?mdm:103411408) transcription initiation factor TFIID subunit 13 |
| [mdm:103423557](https://www.kegg.jp/dbget-bin/www_bget?mdm:103423557) cyclin-H1-1 |
| [mdm03040](https://www.kegg.jp/kegg-bin/show_pathway?157443610656263/mdm03040.args) Spliceosome - Malus domestica (apple) ([20](javascript:display('mdm03040'))) |
|  |
| [mdm:103401169](https://www.kegg.jp/dbget-bin/www_bget?mdm:103401169) U1 small nuclear ribonucleoprotein 70 kDa |
| [mdm:103401225](https://www.kegg.jp/dbget-bin/www_bget?mdm:103401225) probable small nuclear ribonucleoprotein Sm D2 |
| [mdm:103401317](https://www.kegg.jp/dbget-bin/www_bget?mdm:103401317) serine/arginine-rich splicing factor RS41-like isoform X1 |
| [mdm:103401693](https://www.kegg.jp/dbget-bin/www_bget?mdm:103401693) LOW QUALITY PROTEIN: apoptotic chromatin condensation inducer in the nucleus |
| [mdm:103422914](https://www.kegg.jp/dbget-bin/www_bget?mdm:103422914) beta-catenin-like protein 1 |
| [mdm:103423211](https://www.kegg.jp/dbget-bin/www_bget?mdm:103423211) heat shock cognate 70 kDa protein-like |
| [mdm:103426377](https://www.kegg.jp/dbget-bin/www_bget?mdm:103426377) spliceosome-associated protein 130 A-like |
| [mdm:103429221](https://www.kegg.jp/dbget-bin/www_bget?mdm:103429221) pre-mRNA-processing protein 40A-like isoform X1 |
| [mdm:103434172](https://www.kegg.jp/dbget-bin/www_bget?mdm:103434172) heat shock cognate 70 kDa protein 2 |
| [mdm:103434725](https://www.kegg.jp/dbget-bin/www_bget?mdm:103434725) pre-mRNA-splicing factor SYF1-like |
| [mdm:103436782](https://www.kegg.jp/dbget-bin/www_bget?mdm:103436782) protein BUD31 homolog 1 |
| [mdm:103437259](https://www.kegg.jp/dbget-bin/www_bget?mdm:103437259) DEAD-box ATP-dependent RNA helicase 56-like isoform X1 |
| [mdm:103439716](https://www.kegg.jp/dbget-bin/www_bget?mdm:103439716) serine/arginine-rich splicing factor RS41 isoform X1 |
| [mdm:103442265](https://www.kegg.jp/dbget-bin/www_bget?mdm:103442265) protein RRC1-like isoform X1 |
| [mdm:103444156](https://www.kegg.jp/dbget-bin/www_bget?mdm:103444156) LOW QUALITY PROTEIN: THO complex subunit 2 |
| [mdm:103446880](https://www.kegg.jp/dbget-bin/www_bget?mdm:103446880) heat shock 70 kDa protein isoform X1 |
| [mdm:103449635](https://www.kegg.jp/dbget-bin/www_bget?mdm:103449635) SNW/SKI-interacting protein A-like |
| [mdm:103455864](https://www.kegg.jp/dbget-bin/www_bget?mdm:103455864) LOW QUALITY PROTEIN: serine/arginine-rich splicing factor SR45a |
| [mdm:103456194](https://www.kegg.jp/dbget-bin/www_bget?mdm:103456194) DEAD-box ATP-dependent RNA helicase 56 |
| [mdm:114819762](https://www.kegg.jp/dbget-bin/www_bget?mdm:114819762) nuclear cap-binding protein subunit 1-like |
| [mdm03010](https://www.kegg.jp/kegg-bin/show_pathway?157443610656263/mdm03010.args) Ribosome - Malus domestica (apple) ([25](javascript:display('mdm03010'))) |
|  |
| [mdm:103400743](https://www.kegg.jp/dbget-bin/www_bget?mdm:103400743) 60S ribosomal protein L6-like isoform X2 |
| [mdm:103402698](https://www.kegg.jp/dbget-bin/www_bget?mdm:103402698) 50S ribosomal protein L15, chloroplastic |
| [mdm:103404217](https://www.kegg.jp/dbget-bin/www_bget?mdm:103404217) uncharacterized protein LOC103404217 |
| [mdm:103406344](https://www.kegg.jp/dbget-bin/www_bget?mdm:103406344) 40S ribosomal protein S15a-1 |
| [mdm:103408875](https://www.kegg.jp/dbget-bin/www_bget?mdm:103408875) 60S ribosomal protein L11 |
| [mdm:103412799](https://www.kegg.jp/dbget-bin/www_bget?mdm:103412799) 50S ribosomal protein L1, chloroplastic-like |
| [mdm:103414082](https://www.kegg.jp/dbget-bin/www_bget?mdm:103414082) 60S ribosomal protein L10-like |
| [mdm:103417987](https://www.kegg.jp/dbget-bin/www_bget?mdm:103417987) uncharacterized protein LOC103417987 |
| [mdm:103419421](https://www.kegg.jp/dbget-bin/www_bget?mdm:103419421) 50S ribosomal protein L17, chloroplastic-like |
| [mdm:103424477](https://www.kegg.jp/dbget-bin/www_bget?mdm:103424477) 60S acidic ribosomal protein P0 |
| [mdm:103425300](https://www.kegg.jp/dbget-bin/www_bget?mdm:103425300) 40S ribosomal protein S2-4-like |
| [mdm:103425782](https://www.kegg.jp/dbget-bin/www_bget?mdm:103425782) 50S ribosomal protein L27, chloroplastic-like |
| [mdm:103434141](https://www.kegg.jp/dbget-bin/www_bget?mdm:103434141) 40S ribosomal protein S24-1 |
| [mdm:103439656](https://www.kegg.jp/dbget-bin/www_bget?mdm:103439656) 40S ribosomal protein S13-like |
| [mdm:103440981](https://www.kegg.jp/dbget-bin/www_bget?mdm:103440981) 50S ribosomal protein L9, chloroplastic-like |
| [mdm:103441602](https://www.kegg.jp/dbget-bin/www_bget?mdm:103441602) 60S ribosomal protein L30 |
| [mdm:103442136](https://www.kegg.jp/dbget-bin/www_bget?mdm:103442136) 60S ribosomal protein L17-2-like |
| [mdm:103442411](https://www.kegg.jp/dbget-bin/www_bget?mdm:103442411) 30S ribosomal protein S9, chloroplastic-like |
| [mdm:103447327](https://www.kegg.jp/dbget-bin/www_bget?mdm:103447327) 40S ribosomal protein S4-3-like |
| [mdm:103447742](https://www.kegg.jp/dbget-bin/www_bget?mdm:103447742) 40S ribosomal protein S15a-1 |
| [mdm:103450718](https://www.kegg.jp/dbget-bin/www_bget?mdm:103450718) 60S ribosomal protein L36-3-like |
| [mdm:108172127](https://www.kegg.jp/dbget-bin/www_bget?mdm:108172127) uncharacterized protein LOC108172127 |
| [mdm:108172776](https://www.kegg.jp/dbget-bin/www_bget?mdm:108172776) uncharacterized protein LOC108172776 |
| [mdm:114826980](https://www.kegg.jp/dbget-bin/www_bget?mdm:114826980) 60S ribosomal protein L7-2 |
| [mdm:114827017](https://www.kegg.jp/dbget-bin/www_bget?mdm:114827017) 60S ribosomal protein L37-3-like |
| [mdm00970](https://www.kegg.jp/kegg-bin/show_pathway?157443610656263/mdm00970.args) Aminoacyl-tRNA biosynthesis - Malus domestica (apple) ([5](javascript:display('mdm00970'))) |
|  |
| [mdm:103430605](https://www.kegg.jp/dbget-bin/www_bget?mdm:103430605) alanine--tRNA ligase-like |
| [mdm:103440196](https://www.kegg.jp/dbget-bin/www_bget?mdm:103440196) VS; valine--tRNA ligase, mitochondrial 1 isoform X1 |
| [mdm:103444500](https://www.kegg.jp/dbget-bin/www_bget?mdm:103444500) LOW QUALITY PROTEIN: histidine--tRNA ligase, cytoplasmic-like |
| [mdm:103446741](https://www.kegg.jp/dbget-bin/www_bget?mdm:103446741) leucine--tRNA ligase, chloroplastic/mitochondrial-like |
| [mdm:114826512](https://www.kegg.jp/dbget-bin/www_bget?mdm:114826512) glutamyl-tRNA(Gln) amidotransferase subunit C, chloroplastic/mitochondrial |
| [mdm03013](https://www.kegg.jp/kegg-bin/show_pathway?157443610656263/mdm03013.args) RNA transport - Malus domestica (apple) ([24](javascript:display('mdm03013'))) |
|  |
| [mdm:103400227](https://www.kegg.jp/dbget-bin/www_bget?mdm:103400227) protein arginine N-methyltransferase 1.5-like isoform X1 |
| [mdm:103401693](https://www.kegg.jp/dbget-bin/www_bget?mdm:103401693) LOW QUALITY PROTEIN: apoptotic chromatin condensation inducer in the nucleus |
| [mdm:103401870](https://www.kegg.jp/dbget-bin/www_bget?mdm:103401870) probable eukaryotic translation initiation factor 5-1 |
| [mdm:103402313](https://www.kegg.jp/dbget-bin/www_bget?mdm:103402313) partner of Y14 and mago |
| [mdm:103405427](https://www.kegg.jp/dbget-bin/www_bget?mdm:103405427) nuclear pore complex protein NUP35-like |
| [mdm:103406818](https://www.kegg.jp/dbget-bin/www_bget?mdm:103406818) DEAD-box ATP-dependent RNA helicase 12-like |
| [mdm:103408685](https://www.kegg.jp/dbget-bin/www_bget?mdm:103408685) eukaryotic translation initiation factor 3 subunit B-like |
| [mdm:103409468](https://www.kegg.jp/dbget-bin/www_bget?mdm:103409468) regulator of nonsense transcripts 1 homolog isoform X1 |
| [mdm:103434948](https://www.kegg.jp/dbget-bin/www_bget?mdm:103434948) eukaryotic translation initiation factor 4E-1 |
| [mdm:103436857](https://www.kegg.jp/dbget-bin/www_bget?mdm:103436857) eukaryotic translation initiation factor 3 subunit J-like |
| [mdm:103437259](https://www.kegg.jp/dbget-bin/www_bget?mdm:103437259) DEAD-box ATP-dependent RNA helicase 56-like isoform X1 |
| [mdm:103437950](https://www.kegg.jp/dbget-bin/www_bget?mdm:103437950) translation initiation factor eIF-2B subunit delta |
| [mdm:103439440](https://www.kegg.jp/dbget-bin/www_bget?mdm:103439440) 29 kDa ribonucleoprotein A, chloroplastic |
| [mdm:103443828](https://www.kegg.jp/dbget-bin/www_bget?mdm:103443828) protein transport protein SEC13 homolog B-like |
| [mdm:103443831](https://www.kegg.jp/dbget-bin/www_bget?mdm:103443831) nuclear pore complex protein NUP35 |
| [mdm:103444156](https://www.kegg.jp/dbget-bin/www_bget?mdm:103444156) LOW QUALITY PROTEIN: THO complex subunit 2 |
| [mdm:103444634](https://www.kegg.jp/dbget-bin/www_bget?mdm:103444634) THO complex subunit 7A-like |
| [mdm:103444636](https://www.kegg.jp/dbget-bin/www_bget?mdm:103444636) eukaryotic translation initiation factor 5B-like |
| [mdm:103446398](https://www.kegg.jp/dbget-bin/www_bget?mdm:103446398) eukaryotic translation initiation factor 3 subunit D-like |
| [mdm:103447499](https://www.kegg.jp/dbget-bin/www_bget?mdm:103447499) eukaryotic translation initiation factor 3 subunit G-A-like |
| [mdm:103448370](https://www.kegg.jp/dbget-bin/www_bget?mdm:103448370) small ubiquitin-related modifier 1 |
| [mdm:103453883](https://www.kegg.jp/dbget-bin/www_bget?mdm:103453883) eukaryotic translation initiation factor NCBP-like |
| [mdm:103456194](https://www.kegg.jp/dbget-bin/www_bget?mdm:103456194) DEAD-box ATP-dependent RNA helicase 56 |
| [mdm:114819762](https://www.kegg.jp/dbget-bin/www_bget?mdm:114819762) nuclear cap-binding protein subunit 1-like |
| [mdm03015](https://www.kegg.jp/kegg-bin/show_pathway?157443610656263/mdm03015.args) mRNA surveillance pathway - Malus domestica (apple) ([16](javascript:display('mdm03015'))) |
|  |
| [mdm:103401693](https://www.kegg.jp/dbget-bin/www_bget?mdm:103401693) LOW QUALITY PROTEIN: apoptotic chromatin condensation inducer in the nucleus |
| [mdm:103402313](https://www.kegg.jp/dbget-bin/www_bget?mdm:103402313) partner of Y14 and mago |
| [mdm:103409468](https://www.kegg.jp/dbget-bin/www_bget?mdm:103409468) regulator of nonsense transcripts 1 homolog isoform X1 |
| [mdm:103429119](https://www.kegg.jp/dbget-bin/www_bget?mdm:103429119) protein ANTHESIS POMOTING FACTOR 1 |
| [mdm:103431256](https://www.kegg.jp/dbget-bin/www_bget?mdm:103431256) RNA polymerase II subunit A C-terminal domain phosphatase SSU72-like |
| [mdm:103434391](https://www.kegg.jp/dbget-bin/www_bget?mdm:103434391) serine/threonine-protein phosphatase PP2A-4 catalytic subunit |
| [mdm:103435642](https://www.kegg.jp/dbget-bin/www_bget?mdm:103435642) 30-kDa cleavage and polyadenylation specificity factor 30-like |
| [mdm:103437259](https://www.kegg.jp/dbget-bin/www_bget?mdm:103437259) DEAD-box ATP-dependent RNA helicase 56-like isoform X1 |
| [mdm:103439440](https://www.kegg.jp/dbget-bin/www_bget?mdm:103439440) 29 kDa ribonucleoprotein A, chloroplastic |
| [mdm:103439774](https://www.kegg.jp/dbget-bin/www_bget?mdm:103439774) LOW QUALITY PROTEIN: cleavage and polyadenylation specificity factor subunit 2 |
| [mdm:103444822](https://www.kegg.jp/dbget-bin/www_bget?mdm:103444822) polyadenylate-binding protein 2 isoform X1 |
| [mdm:103449447](https://www.kegg.jp/dbget-bin/www_bget?mdm:103449447) polyadenylation and cleavage factor homolog 4 |
| [mdm:103450536](https://www.kegg.jp/dbget-bin/www_bget?mdm:103450536) mRNA-capping enzyme isoform X1 |
| [mdm:103451061](https://www.kegg.jp/dbget-bin/www_bget?mdm:103451061) serine/threonine-protein phosphatase PP1 isozyme 3-like |
| [mdm:103456194](https://www.kegg.jp/dbget-bin/www_bget?mdm:103456194) DEAD-box ATP-dependent RNA helicase 56 |
| [mdm:114819762](https://www.kegg.jp/dbget-bin/www_bget?mdm:114819762) nuclear cap-binding protein subunit 1-like |
| [mdm03008](https://www.kegg.jp/kegg-bin/show_pathway?157443610656263/mdm03008.args) Ribosome biogenesis in eukaryotes - Malus domestica (apple) ([5](javascript:display('mdm03008'))) |
|  |
| [mdm:103401481](https://www.kegg.jp/dbget-bin/www_bget?mdm:103401481) LOW QUALITY PROTEIN: casein kinase II subunit alpha-2-like |
| [mdm:103403915](https://www.kegg.jp/dbget-bin/www_bget?mdm:103403915) casein kinase II subunit alpha isoform X1 |
| [mdm:103429432](https://www.kegg.jp/dbget-bin/www_bget?mdm:103429432) small RNA degrading nuclease 5-like |
| [mdm:103443560](https://www.kegg.jp/dbget-bin/www_bget?mdm:103443560) ribosome biogenesis protein bms1-like |
| [mdm:103454576](https://www.kegg.jp/dbget-bin/www_bget?mdm:103454576) ribosomal RNA small subunit methyltransferase nep-1 |
| [mdm03060](https://www.kegg.jp/kegg-bin/show_pathway?157443610656263/mdm03060.args) Protein export - Malus domestica (apple) ([1](javascript:display('mdm03060'))) |
|  |
| [mdm:103450083](https://www.kegg.jp/dbget-bin/www_bget?mdm:103450083) protein transport protein Sec61 subunit beta-like |
| [mdm04141](https://www.kegg.jp/kegg-bin/show_pathway?157443610656263/mdm04141.args) Protein processing in endoplasmic reticulum - Malus domestica (apple) ([22](javascript:display('mdm04141'))) |
|  |
| [mdm:103404484](https://www.kegg.jp/dbget-bin/www_bget?mdm:103404484) probable ubiquitin conjugation factor E4 |
| [mdm:103405253](https://www.kegg.jp/dbget-bin/www_bget?mdm:103405253) RING-box protein 1a |
| [mdm:103405456](https://www.kegg.jp/dbget-bin/www_bget?mdm:103405456) protein disulfide isomerase-like 1-6 isoform X1 |
| [mdm:103406609](https://www.kegg.jp/dbget-bin/www_bget?mdm:103406609) LOW QUALITY PROTEIN: 18.2 kDa class I heat shock protein |
| [mdm:103412153](https://www.kegg.jp/dbget-bin/www_bget?mdm:103412153) E3 ubiquitin-protein ligase CHIP-like isoform X1 |
| [mdm:103418602](https://www.kegg.jp/dbget-bin/www_bget?mdm:103418602) E3 ubiquitin-protein ligase RNF168-like |
| [mdm:103422465](https://www.kegg.jp/dbget-bin/www_bget?mdm:103422465) derlin-2.2 |
| [mdm:103423211](https://www.kegg.jp/dbget-bin/www_bget?mdm:103423211) heat shock cognate 70 kDa protein-like |
| [mdm:103431755](https://www.kegg.jp/dbget-bin/www_bget?mdm:103431755) protein disulfide-isomerase 5-2-like |
| [mdm:103431823](https://www.kegg.jp/dbget-bin/www_bget?mdm:103431823) ubiquitin receptor RAD23c-like |
| [mdm:103432482](https://www.kegg.jp/dbget-bin/www_bget?mdm:103432482) NPL4-like protein 2 |
| [mdm:103434172](https://www.kegg.jp/dbget-bin/www_bget?mdm:103434172) heat shock cognate 70 kDa protein 2 |
| [mdm:103436223](https://www.kegg.jp/dbget-bin/www_bget?mdm:103436223) SKP1-like protein 1A |
| [mdm:103436955](https://www.kegg.jp/dbget-bin/www_bget?mdm:103436955) 17.1 kDa class II heat shock protein-like |
| [mdm:103438978](https://www.kegg.jp/dbget-bin/www_bget?mdm:103438978) chaperone protein dnaJ A6-like |
| [mdm:103440661](https://www.kegg.jp/dbget-bin/www_bget?mdm:103440661) LOW QUALITY PROTEIN: dolichyl-diphosphooligosaccharide--protein glycosyltransferase subunit 1B-like |
| [mdm:103443828](https://www.kegg.jp/dbget-bin/www_bget?mdm:103443828) protein transport protein SEC13 homolog B-like |
| [mdm:103446712](https://www.kegg.jp/dbget-bin/www_bget?mdm:103446712) 15.4 kDa class V heat shock protein |
| [mdm:103446880](https://www.kegg.jp/dbget-bin/www_bget?mdm:103446880) heat shock 70 kDa protein isoform X1 |
| [mdm:103450083](https://www.kegg.jp/dbget-bin/www_bget?mdm:103450083) protein transport protein Sec61 subunit beta-like |
| [mdm:103451159](https://www.kegg.jp/dbget-bin/www_bget?mdm:103451159) calreticulin-3-like |
| [mdm:114825618](https://www.kegg.jp/dbget-bin/www_bget?mdm:114825618) small heat shock protein, chloroplastic-like |
| [mdm04120](https://www.kegg.jp/kegg-bin/show_pathway?157443610656263/mdm04120.args) Ubiquitin mediated proteolysis - Malus domestica (apple) ([11](javascript:display('mdm04120'))) |
|  |
| [mdm:103400842](https://www.kegg.jp/dbget-bin/www_bget?mdm:103400842) probable ubiquitin-conjugating enzyme E2 18 |
| [mdm:103403897](https://www.kegg.jp/dbget-bin/www_bget?mdm:103403897) NEDD8-activating enzyme E1 catalytic subunit |
| [mdm:103404484](https://www.kegg.jp/dbget-bin/www_bget?mdm:103404484) probable ubiquitin conjugation factor E4 |
| [mdm:103405253](https://www.kegg.jp/dbget-bin/www_bget?mdm:103405253) RING-box protein 1a |
| [mdm:103409644](https://www.kegg.jp/dbget-bin/www_bget?mdm:103409644) SUMO-activating enzyme subunit 1A-like |
| [mdm:103411193](https://www.kegg.jp/dbget-bin/www_bget?mdm:103411193) cullin-3A-like |
| [mdm:103412153](https://www.kegg.jp/dbget-bin/www_bget?mdm:103412153) E3 ubiquitin-protein ligase CHIP-like isoform X1 |
| [mdm:103436223](https://www.kegg.jp/dbget-bin/www_bget?mdm:103436223) SKP1-like protein 1A |
| [mdm:103443714](https://www.kegg.jp/dbget-bin/www_bget?mdm:103443714) E3 ubiquitin-protein ligase SINAT5 |
| [mdm:103452655](https://www.kegg.jp/dbget-bin/www_bget?mdm:103452655) E3 ubiquitin-protein ligase UPL6-like |
| [mdm:103454985](https://www.kegg.jp/dbget-bin/www_bget?mdm:103454985) SUMO-activating enzyme subunit 1B-1-like |
| [mdm04122](https://www.kegg.jp/kegg-bin/show_pathway?157443610656263/mdm04122.args) Sulfur relay system - Malus domestica (apple) ([2](javascript:display('mdm04122'))) |
|  |
| [mdm:103435398](https://www.kegg.jp/dbget-bin/www_bget?mdm:103435398) thiosulfate/3-mercaptopyruvate sulfurtransferase 1, mitochondrial-like |
| [mdm:103445864](https://www.kegg.jp/dbget-bin/www_bget?mdm:103445864) thiosulfate/3-mercaptopyruvate sulfurtransferase 1, mitochondrial-like |
| [mdm03050](https://www.kegg.jp/kegg-bin/show_pathway?157443610656263/mdm03050.args) Proteasome - Malus domestica (apple) ([12](javascript:display('mdm03050'))) |
|  |
| [mdm:103417959](https://www.kegg.jp/dbget-bin/www_bget?mdm:103417959) 26S proteasome regulatory subunit 6B homolog |
| [mdm:103418407](https://www.kegg.jp/dbget-bin/www_bget?mdm:103418407) proteasome subunit beta type-1 |
| [mdm:103428634](https://www.kegg.jp/dbget-bin/www_bget?mdm:103428634) 26S proteasome regulatory subunit 10B homolog A |
| [mdm:103432018](https://www.kegg.jp/dbget-bin/www_bget?mdm:103432018) proteasome subunit beta type-7-B |
| [mdm:103433578](https://www.kegg.jp/dbget-bin/www_bget?mdm:103433578) 26S proteasome non-ATPase regulatory subunit 7 homolog A |
| [mdm:103435285](https://www.kegg.jp/dbget-bin/www_bget?mdm:103435285) proteasome subunit alpha type-5 |
| [mdm:103436261](https://www.kegg.jp/dbget-bin/www_bget?mdm:103436261) 26S proteasome non-ATPase regulatory subunit 4 homolog isoform X1 |
| [mdm:103436270](https://www.kegg.jp/dbget-bin/www_bget?mdm:103436270) 26S proteasome regulatory subunit 7 |
| [mdm:103445819](https://www.kegg.jp/dbget-bin/www_bget?mdm:103445819) proteasome subunit alpha type-2-A-like |
| [mdm:103453143](https://www.kegg.jp/dbget-bin/www_bget?mdm:103453143) proteasome subunit beta type-2-A |
| [mdm:103453636](https://www.kegg.jp/dbget-bin/www_bget?mdm:103453636) proteasome subunit alpha type-6 isoform X1 |
| [mdm:103455036](https://www.kegg.jp/dbget-bin/www_bget?mdm:103455036) 26S proteasome non-ATPase regulatory subunit 6 homolog |
| [mdm03018](https://www.kegg.jp/kegg-bin/show_pathway?157443610656263/mdm03018.args) RNA degradation - Malus domestica (apple) ([8](javascript:display('mdm03018'))) |
|  |
| [mdm:103420714](https://www.kegg.jp/dbget-bin/www_bget?mdm:103420714) uncharacterized protein LOC103420714 |
| [mdm:103430194](https://www.kegg.jp/dbget-bin/www_bget?mdm:103430194) exosome complex component RRP4 homolog |
| [mdm:103439440](https://www.kegg.jp/dbget-bin/www_bget?mdm:103439440) 29 kDa ribonucleoprotein A, chloroplastic |
| [mdm:103440663](https://www.kegg.jp/dbget-bin/www_bget?mdm:103440663) mRNA-decapping enzyme subunit 2-like isoform X1 |
| [mdm:103447522](https://www.kegg.jp/dbget-bin/www_bget?mdm:103447522) uncharacterized protein LOC103447522 |
| [mdm:103447830](https://www.kegg.jp/dbget-bin/www_bget?mdm:103447830) uncharacterized protein LOC103447830 |
| [mdm:103453924](https://www.kegg.jp/dbget-bin/www_bget?mdm:103453924) enolase |
| [mdm:103455692](https://www.kegg.jp/dbget-bin/www_bget?mdm:103455692) general negative regulator of transcription subunit 3-like isoform X1 |
| [mdm03030](https://www.kegg.jp/kegg-bin/show_pathway?157443610656263/mdm03030.args) DNA replication - Malus domestica (apple) ([1](javascript:display('mdm03030'))) |
|  |
| [mdm:103421979](https://www.kegg.jp/dbget-bin/www_bget?mdm:103421979) uncharacterized protein At4g28440-like |
| [mdm03410](https://www.kegg.jp/kegg-bin/show_pathway?157443610656263/mdm03410.args) Base excision repair - Malus domestica (apple) ([2](javascript:display('mdm03410'))) |
|  |
| [mdm:103405499](https://www.kegg.jp/dbget-bin/www_bget?mdm:103405499) DNA-repair protein XRCC1 |
| [mdm:103446994](https://www.kegg.jp/dbget-bin/www_bget?mdm:103446994) uncharacterized protein LOC103446994 |
| [mdm03420](https://www.kegg.jp/kegg-bin/show_pathway?157443610656263/mdm03420.args) Nucleotide excision repair - Malus domestica (apple) ([4](javascript:display('mdm03420'))) |
|  |
| [mdm:103405253](https://www.kegg.jp/dbget-bin/www_bget?mdm:103405253) RING-box protein 1a |
| [mdm:103421979](https://www.kegg.jp/dbget-bin/www_bget?mdm:103421979) uncharacterized protein At4g28440-like |
| [mdm:103423557](https://www.kegg.jp/dbget-bin/www_bget?mdm:103423557) cyclin-H1-1 |
| [mdm:103431823](https://www.kegg.jp/dbget-bin/www_bget?mdm:103431823) ubiquitin receptor RAD23c-like |
| [mdm03430](https://www.kegg.jp/kegg-bin/show_pathway?157443610656263/mdm03430.args) Mismatch repair - Malus domestica (apple) ([1](javascript:display('mdm03430'))) |
|  |
| [mdm:103421979](https://www.kegg.jp/dbget-bin/www_bget?mdm:103421979) uncharacterized protein At4g28440-like |
| [mdm03440](https://www.kegg.jp/kegg-bin/show_pathway?157443610656263/mdm03440.args) Homologous recombination - Malus domestica (apple) ([4](javascript:display('mdm03440'))) |
|  |
| [mdm:103413829](https://www.kegg.jp/dbget-bin/www_bget?mdm:103413829) BRISC and BRCA1-A complex member 2-like |
| [mdm:103420862](https://www.kegg.jp/dbget-bin/www_bget?mdm:103420862) BRISC and BRCA1-A complex member 2-like |
| [mdm:103421979](https://www.kegg.jp/dbget-bin/www_bget?mdm:103421979) uncharacterized protein At4g28440-like |
| [mdm:103439414](https://www.kegg.jp/dbget-bin/www_bget?mdm:103439414) nijmegen breakage syndrome 1 protein |
| [mdm02010](https://www.kegg.jp/kegg-bin/show_pathway?157443610656263/mdm02010.args) ABC transporters - Malus domestica (apple) ([1](javascript:display('mdm02010'))) |
|  |
| [mdm:103433112](https://www.kegg.jp/dbget-bin/www_bget?mdm:103433112) ABC transporter B family member 1 |
| [mdm04016](https://www.kegg.jp/kegg-bin/show_pathway?157443610656263/mdm04016.args) MAPK signaling pathway - plant - Malus domestica (apple) ([9](javascript:display('mdm04016'))) |
|  |
| [mdm:103402159](https://www.kegg.jp/dbget-bin/www_bget?mdm:103402159) transcription factor VIP1-like |
| [mdm:103411041](https://www.kegg.jp/dbget-bin/www_bget?mdm:103411041) serine/threonine-protein kinase SRK2A-like |
| [mdm:103421404](https://www.kegg.jp/dbget-bin/www_bget?mdm:103421404) ETHYLENE INSENSITIVE 3-like 1 protein |
| [mdm:103431796](https://www.kegg.jp/dbget-bin/www_bget?mdm:103431796) ETHYLENE INSENSITIVE 3-like 1 protein |
| [mdm:103441525](https://www.kegg.jp/dbget-bin/www_bget?mdm:103441525) respiratory burst oxidase homolog protein D-like |
| [mdm:103444633](https://www.kegg.jp/dbget-bin/www_bget?mdm:103444633) transcription factor VIP1 |
| [mdm:103449156](https://www.kegg.jp/dbget-bin/www_bget?mdm:103449156) ethylene response sensor 1-like |
| [mdm:103456346](https://www.kegg.jp/dbget-bin/www_bget?mdm:103456346) mitogen-activated protein kinase homolog D5 |
| [mdm:114820220](https://www.kegg.jp/dbget-bin/www_bget?mdm:114820220) calmodulin-7 |
| [mdm04070](https://www.kegg.jp/kegg-bin/show_pathway?157443610656263/mdm04070.args) Phosphatidylinositol signaling system - Malus domestica (apple) ([8](javascript:display('mdm04070'))) |
|  |
| [mdm:103406415](https://www.kegg.jp/dbget-bin/www_bget?mdm:103406415) phosphoinositide phospholipase C 2-like |
| [mdm:103414619](https://www.kegg.jp/dbget-bin/www_bget?mdm:103414619) phosphoinositide phosphatase SAC8-like |
| [mdm:103422491](https://www.kegg.jp/dbget-bin/www_bget?mdm:103422491) inositol-phosphate phosphatase-like |
| [mdm:103438670](https://www.kegg.jp/dbget-bin/www_bget?mdm:103438670) LOW QUALITY PROTEIN: inositol polyphosphate multikinase beta-like |
| [mdm:103441898](https://www.kegg.jp/dbget-bin/www_bget?mdm:103441898) phosphatidylinositol 4-phosphate 5-kinase 8-like isoform X1 |
| [mdm:103444921](https://www.kegg.jp/dbget-bin/www_bget?mdm:103444921) inositol-tetrakisphosphate 1-kinase 1-like |
| [mdm:114820220](https://www.kegg.jp/dbget-bin/www_bget?mdm:114820220) calmodulin-7 |
| [mdm:114827525](https://www.kegg.jp/dbget-bin/www_bget?mdm:114827525) putative 1-phosphatidylinositol-3-phosphate 5-kinase FAB1D |
| [mdm04075](https://www.kegg.jp/kegg-bin/show_pathway?157443610656263/mdm04075.args) Plant hormone signal transduction - Malus domestica (apple) ([15](javascript:display('mdm04075'))) |
|  |
| [mdm:103401867](https://www.kegg.jp/dbget-bin/www_bget?mdm:103401867) transcription factor TGA7 |
| [mdm:103403806](https://www.kegg.jp/dbget-bin/www_bget?mdm:103403806) histidine-containing phosphotransfer protein 1 |
| [mdm:103411041](https://www.kegg.jp/dbget-bin/www_bget?mdm:103411041) serine/threonine-protein kinase SRK2A-like |
| [mdm:103420869](https://www.kegg.jp/dbget-bin/www_bget?mdm:103420869) auxin response factor 5-like |
| [mdm:103421404](https://www.kegg.jp/dbget-bin/www_bget?mdm:103421404) ETHYLENE INSENSITIVE 3-like 1 protein |
| [mdm:103424687](https://www.kegg.jp/dbget-bin/www_bget?mdm:103424687) BRI1 kinase inhibitor 1-like |
| [mdm:103430506](https://www.kegg.jp/dbget-bin/www_bget?mdm:103430506) auxin-responsive protein IAA3 |
| [mdm:103431796](https://www.kegg.jp/dbget-bin/www_bget?mdm:103431796) ETHYLENE INSENSITIVE 3-like 1 protein |
| [mdm:103432473](https://www.kegg.jp/dbget-bin/www_bget?mdm:103432473) histidine-containing phosphotransfer protein 1 |
| [mdm:103433823](https://www.kegg.jp/dbget-bin/www_bget?mdm:103433823) transcription factor PIF3 isoform X1 |
| [mdm:103434165](https://www.kegg.jp/dbget-bin/www_bget?mdm:103434165) IAA4; auxin-responsive protein IAA3-like |
| [mdm:103446364](https://www.kegg.jp/dbget-bin/www_bget?mdm:103446364) ARF3; auxin response factor 3 |
| [mdm:103449156](https://www.kegg.jp/dbget-bin/www_bget?mdm:103449156) ethylene response sensor 1-like |
| [mdm:103451320](https://www.kegg.jp/dbget-bin/www_bget?mdm:103451320) protein TIFY 10A |
| [mdm:103456346](https://www.kegg.jp/dbget-bin/www_bget?mdm:103456346) mitogen-activated protein kinase homolog D5 |
| [mdm04144](https://www.kegg.jp/kegg-bin/show_pathway?157443610656263/mdm04144.args) Endocytosis - Malus domestica (apple) ([22](javascript:display('mdm04144'))) |
|  |
| [mdm:103400745](https://www.kegg.jp/dbget-bin/www_bget?mdm:103400745) vacuolar protein sorting-associated protein 32 homolog 2 |
| [mdm:103402341](https://www.kegg.jp/dbget-bin/www_bget?mdm:103402341) actin-related protein 2/3 complex subunit 5A-like |
| [mdm:103403013](https://www.kegg.jp/dbget-bin/www_bget?mdm:103403013) AMSH-like ubiquitin thioesterase 2 isoform X3 |
| [mdm:103404322](https://www.kegg.jp/dbget-bin/www_bget?mdm:103404322) ADP-ribosylation factor 1 |
| [mdm:103406371](https://www.kegg.jp/dbget-bin/www_bget?mdm:103406371) actin-related protein 2/3 complex subunit 5A isoform X1 |
| [mdm:103410813](https://www.kegg.jp/dbget-bin/www_bget?mdm:103410813) protein SUPPRESSOR OF K(+) TRANSPORT GROWTH DEFECT 1 |
| [mdm:103423030](https://www.kegg.jp/dbget-bin/www_bget?mdm:103423030) vacuolar protein sorting-associated protein 25 |
| [mdm:103423211](https://www.kegg.jp/dbget-bin/www_bget?mdm:103423211) heat shock cognate 70 kDa protein-like |
| [mdm:103423743](https://www.kegg.jp/dbget-bin/www_bget?mdm:103423743) LOW QUALITY PROTEIN: IST1 homolog |
| [mdm:103426994](https://www.kegg.jp/dbget-bin/www_bget?mdm:103426994) protein EARLY-RESPONSIVE TO DEHYDRATION 7, chloroplastic-like |
| [mdm:103428437](https://www.kegg.jp/dbget-bin/www_bget?mdm:103428437) vacuolar protein sorting-associated protein 36-like |
| [mdm:103434172](https://www.kegg.jp/dbget-bin/www_bget?mdm:103434172) heat shock cognate 70 kDa protein 2 |
| [mdm:103435723](https://www.kegg.jp/dbget-bin/www_bget?mdm:103435723) vacuolar protein sorting-associated protein 2 homolog 3-like |
| [mdm:103437260](https://www.kegg.jp/dbget-bin/www_bget?mdm:103437260) phospholipase D alpha 1 |
| [mdm:103441898](https://www.kegg.jp/dbget-bin/www_bget?mdm:103441898) phosphatidylinositol 4-phosphate 5-kinase 8-like isoform X1 |
| [mdm:103443905](https://www.kegg.jp/dbget-bin/www_bget?mdm:103443905) IST1-like protein |
| [mdm:103444296](https://www.kegg.jp/dbget-bin/www_bget?mdm:103444296) ARF; ADP-ribosylation factor |
| [mdm:103446880](https://www.kegg.jp/dbget-bin/www_bget?mdm:103446880) heat shock 70 kDa protein isoform X1 |
| [mdm:103447744](https://www.kegg.jp/dbget-bin/www_bget?mdm:103447744) ras-related protein RABE1c-like |
| [mdm:103448507](https://www.kegg.jp/dbget-bin/www_bget?mdm:103448507) vacuolar protein sorting-associated protein 22 homolog 1-like |
| [mdm:103454250](https://www.kegg.jp/dbget-bin/www_bget?mdm:103454250) ras-related protein Rab7 |
| [mdm:103455767](https://www.kegg.jp/dbget-bin/www_bget?mdm:103455767) uncharacterized protein LOC103455767 |
| [mdm04145](https://www.kegg.jp/kegg-bin/show_pathway?157443610656263/mdm04145.args) Phagosome - Malus domestica (apple) ([9](javascript:display('mdm04145'))) |
|  |
| [mdm:103417231](https://www.kegg.jp/dbget-bin/www_bget?mdm:103417231) V-type proton ATPase subunit D-like |
| [mdm:103442089](https://www.kegg.jp/dbget-bin/www_bget?mdm:103442089) V-type proton ATPase subunit C |
| [mdm:103442796](https://www.kegg.jp/dbget-bin/www_bget?mdm:103442796) V-type proton ATPase subunit d2 |
| [mdm:103450083](https://www.kegg.jp/dbget-bin/www_bget?mdm:103450083) protein transport protein Sec61 subunit beta-like |
| [mdm:103450546](https://www.kegg.jp/dbget-bin/www_bget?mdm:103450546) V-type proton ATPase 16 kDa proteolipid subunit |
| [mdm:103451159](https://www.kegg.jp/dbget-bin/www_bget?mdm:103451159) calreticulin-3-like |
| [mdm:103454250](https://www.kegg.jp/dbget-bin/www_bget?mdm:103454250) ras-related protein Rab7 |
| [mdm:103455244](https://www.kegg.jp/dbget-bin/www_bget?mdm:103455244) rac-like GTP-binding protein ARAC8 |
| [mdm:114827525](https://www.kegg.jp/dbget-bin/www_bget?mdm:114827525) putative 1-phosphatidylinositol-3-phosphate 5-kinase FAB1D |
| [mdm04146](https://www.kegg.jp/kegg-bin/show_pathway?157443610656263/mdm04146.args) Peroxisome - Malus domestica (apple) ([9](javascript:display('mdm04146'))) |
|  |
| [mdm:103402584](https://www.kegg.jp/dbget-bin/www_bget?mdm:103402584) phytanoyl-CoA dioxygenase |
| [mdm:103411515](https://www.kegg.jp/dbget-bin/www_bget?mdm:103411515) long chain acyl-CoA synthetase 6, peroxisomal-like isoform X1 |
| [mdm:103421889](https://www.kegg.jp/dbget-bin/www_bget?mdm:103421889) 2-hydroxyacyl-CoA lyase-like |
| [mdm:103426425](https://www.kegg.jp/dbget-bin/www_bget?mdm:103426425) superoxide dismutase [Mn], mitochondrial-like isoform X2 |
| [mdm:103437718](https://www.kegg.jp/dbget-bin/www_bget?mdm:103437718) peroxisomal membrane protein PEX14-like isoform X2 |
| [mdm:103445750](https://www.kegg.jp/dbget-bin/www_bget?mdm:103445750) peroxisomal (S)-2-hydroxy-acid oxidase-like |
| [mdm:103445870](https://www.kegg.jp/dbget-bin/www_bget?mdm:103445870) peroxisomal 2,4-dienoyl-CoA reductase-like |
| [mdm:103450984](https://www.kegg.jp/dbget-bin/www_bget?mdm:103450984) superoxide dismutase [Cu-Zn] 1-like |
| [mdm:103455220](https://www.kegg.jp/dbget-bin/www_bget?mdm:103455220) peroxisomal membrane protein PEX14 isoform X1 |
| [mdm04136](https://www.kegg.jp/kegg-bin/show_pathway?157443610656263/mdm04136.args) Autophagy - other - Malus domestica (apple) ([4](javascript:display('mdm04136'))) |
|  |
| [mdm:103417629](https://www.kegg.jp/dbget-bin/www_bget?mdm:103417629) beclin-1-like protein |
| [mdm:103432776](https://www.kegg.jp/dbget-bin/www_bget?mdm:103432776) autophagy-related protein 11 |
| [mdm:103434391](https://www.kegg.jp/dbget-bin/www_bget?mdm:103434391) serine/threonine-protein phosphatase PP2A-4 catalytic subunit |
| [mdm:103438347](https://www.kegg.jp/dbget-bin/www_bget?mdm:103438347) cysteine protease ATG4 isoform X1 |
| [mdm04712](https://www.kegg.jp/kegg-bin/show_pathway?157443610656263/mdm04712.args) Circadian rhythm - plant - Malus domestica (apple) ([6](javascript:display('mdm04712'))) |
|  |
| [mdm:103401481](https://www.kegg.jp/dbget-bin/www_bget?mdm:103401481) LOW QUALITY PROTEIN: casein kinase II subunit alpha-2-like |
| [mdm:103403915](https://www.kegg.jp/dbget-bin/www_bget?mdm:103403915) casein kinase II subunit alpha isoform X1 |
| [mdm:103433124](https://www.kegg.jp/dbget-bin/www_bget?mdm:103433124) transcription factor HY5-like isoform X1 |
| [mdm:103433823](https://www.kegg.jp/dbget-bin/www_bget?mdm:103433823) transcription factor PIF3 isoform X1 |
| [mdm:103443512](https://www.kegg.jp/dbget-bin/www_bget?mdm:103443512) polyketide synthase 5-like |
| [mdm:103453329](https://www.kegg.jp/dbget-bin/www_bget?mdm:103453329) LOW QUALITY PROTEIN: cryptochrome-1 |
| [mdm04626](https://www.kegg.jp/kegg-bin/show_pathway?157443610656263/mdm04626.args) Plant-pathogen interaction - Malus domestica (apple) ([10](javascript:display('mdm04626'))) |
|  |
| [mdm:103402122](https://www.kegg.jp/dbget-bin/www_bget?mdm:103402122) calcium-dependent protein kinase 8-like isoform X1 |
| [mdm:103405957](https://www.kegg.jp/dbget-bin/www_bget?mdm:103405957) LOW QUALITY PROTEIN: pto-interacting protein 1 |
| [mdm:103409441](https://www.kegg.jp/dbget-bin/www_bget?mdm:103409441) disease resistance protein RPM1-like |
| [mdm:103427684](https://www.kegg.jp/dbget-bin/www_bget?mdm:103427684) probable calcium-binding protein CML49 isoform X3 |
| [mdm:103439883](https://www.kegg.jp/dbget-bin/www_bget?mdm:103439883) cyclic nucleotide-gated ion channel 1-like isoform X4 |
| [mdm:103441525](https://www.kegg.jp/dbget-bin/www_bget?mdm:103441525) respiratory burst oxidase homolog protein D-like |
| [mdm:103445116](https://www.kegg.jp/dbget-bin/www_bget?mdm:103445116) LOW QUALITY PROTEIN: cysteine protease RD19A |
| [mdm:103451420](https://www.kegg.jp/dbget-bin/www_bget?mdm:103451420) WRKY transcription factor SUSIBA2-like |
| [mdm:103456346](https://www.kegg.jp/dbget-bin/www_bget?mdm:103456346) mitogen-activated protein kinase homolog D5 |
| [mdm:114820220](https://www.kegg.jp/dbget-bin/www_bget?mdm:114820220) calmodulin-7 |
| [mdm04933](https://www.kegg.jp/kegg-bin/show_pathway?157443610656263/mdm04933.args) AGE-RAGE signaling pathway in diabetic complications - Malus domestica (apple) ([3](javascript:display('mdm04933'))) |
|  |
| [mdm:103406415](https://www.kegg.jp/dbget-bin/www_bget?mdm:103406415) phosphoinositide phospholipase C 2-like |
| [mdm:103447828](https://www.kegg.jp/dbget-bin/www_bget?mdm:103447828) mitogen-activated protein kinase homolog NTF6-like |
| [mdm:103455244](https://www.kegg.jp/dbget-bin/www_bget?mdm:103455244) rac-like GTP-binding protein ARAC8 |
| [mdm01502](https://www.kegg.jp/kegg-bin/show_pathway?157443610656263/mdm01502.args) Vancomycin resistance - Malus domestica (apple) ([1](javascript:display('mdm01502'))) |
