# Supplementary figures and images for: Fox Hunting in Wild Apples: Searching for Novel Genes in Malus Sieversii
Source: Int J Mol Sci. 2020 Dec 14;21(24):9516. doi: 10.3390/ijms21249516 (PMC7765095; doi:10.3390/ijms21249516)

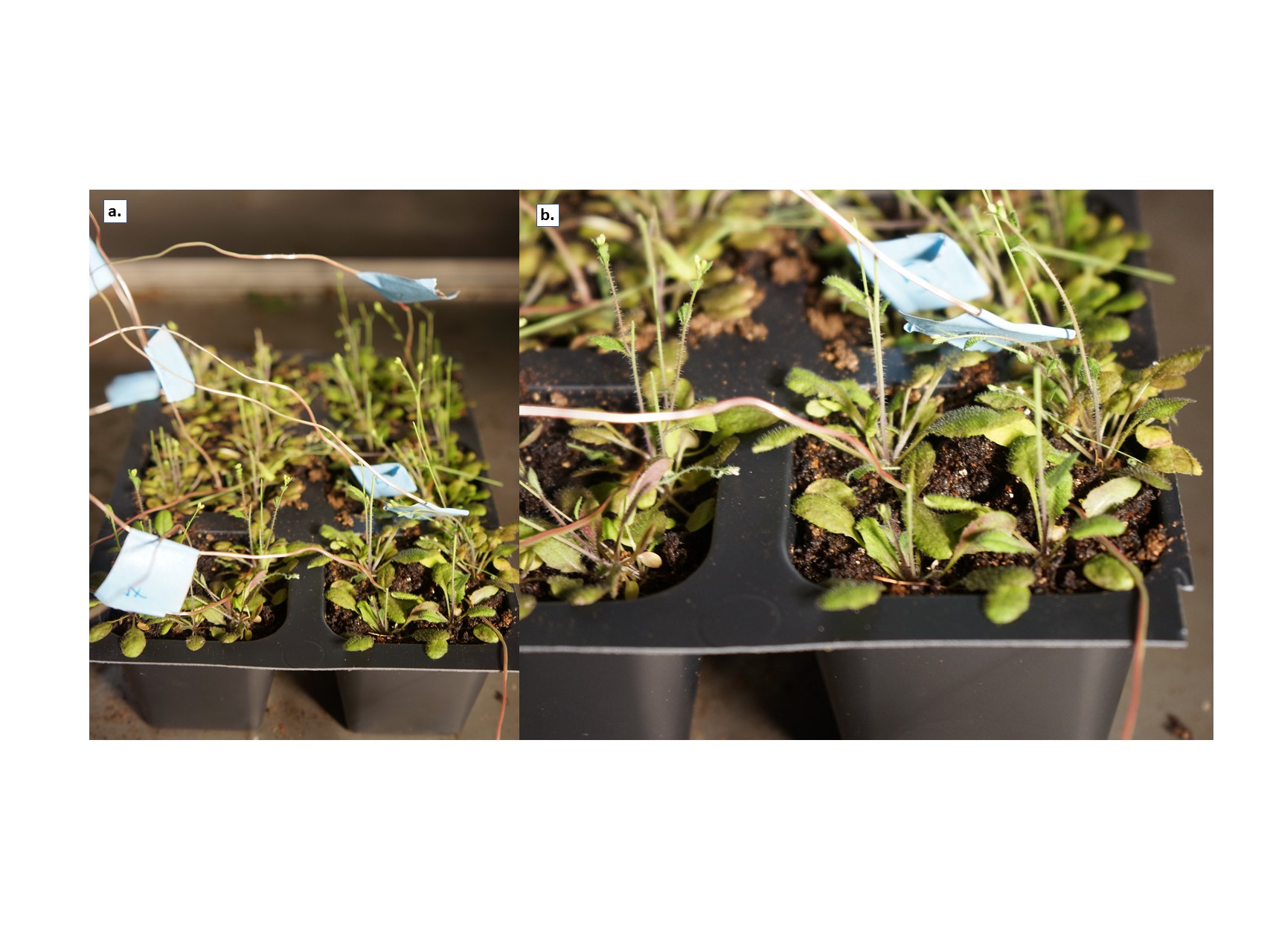

Supplement: Supplementary file 1 [file ijms-21-09516-s001.zip › Supplementary Figure 1.jpg]
